# O-GlcNAcylation of glutaminase isoform KGA inhibits ferroptosis through activation of glutaminolysis in hepatoblastoma

**DOI:** 10.1038/s41420-025-02464-2

**Published:** 2025-04-09

**Authors:** Sijia Fang, Guoqing Zhu, Yi Xie, Miao Ding, Ni Zhen, Jiabei Zhu, Siwei Mao, Xiaochen Tang, Han Wu, Qi Zhang, Aijia Zhang, Xin Ni, Qiuhui Pan, Ji Ma

**Affiliations:** 1 Clinical Laboratory, Shanghai Children’s Medical Center, Shanghai Jiao Tong University School of Medicine, Shanghai, China; 2 Beijing Key Laboratory for Pediatric Diseases of Otolaryngology, Head and Neck Surgery, MOE Key Laboratory of Major Diseases in Children, Beijing Pediatric Research Institute, Beijing Children’s Hospital, Capital Medical University, National Center for Children’s Health (NCCH), Beijing, China; 3 Department of Otolaryngology, Head and Neck Surgery, Beijing Children’s Hospital, Capital Medical University, National Center for Children’s Health (NCCH), Beijing, China; 4 Shanghai Key Laboratory of Clinical Molecular Diagnostics for Pediatrics, Shanghai, China; 5 Faculty of Medical Laboratory Science, College of Health Science and Technology, Shanghai Jiao Tong University School of Medicine, Shanghai, China; 6 Sanya Women and Children’s Hospital Managed by Shanghai Children’s Medical Center, Sanya, China

**Keywords:** Cell biology, Cancer metabolism

## Abstract

Hepatoblastoma (HB), the most common pediatric hepatic malignancy, exhibits an increasing incidence. Metabolism reprogramming represents a pivotal hallmark in the oncogenic transformation process, with glutamine emerging as a critical energy source for neoplastic cells, rivaling glucose. However, the mechanism by which glutamine is involved in the development of HB remains unclear. Our study identified glutamine metabolism as a crucial factor in the development of HB. The key enzyme of glutamine metabolism, kidney-type glutaminase (GLS1), is activated in HB and regulates cell proliferation. Mechanistically, the GLS1 subtype KGA, utilizing glutamate derived from glutaminolysis, enhances glutathione (GSH) synthesis, which in turn inhibits ferroptosis in HB cells. Importantly, the Thr563 residue of KGA undergoes O-GlcNAcylation, enhancing enzyme activity and stability, accelerating glutaminolysis, and promoting the proliferation of HB. This study demonstrated that enhanced glutaminolysis, driven by GLS1, is crucial for the development of HB by inhibiting ferroptosis. The O-GlcNAcylation of KGA isoform ensures its stability and glutaminase function in HB cells, which can serve as a promising therapeutic target for KGA-mediated glutaminolysis in HB.

## Introduction

Hepatoblastoma (HB) arises from the abnormal development of undifferentiated hepatic progenitor cells during embryogenesis [1]. It is the most common primary liver tumor and the third most prevalent abdominal malignancy in children, exhibiting an incremental incidence in recent decades [2]. Although the current clinical treatment approach for HB, including surgical resection and chemotherapy, has significantly improved patients’ outcomes, there are still challenges in managing advanced cases [3, 4]. Hence, it is crucial to investigate the mechanisms underlying the pathogenesis of HB and identify effective biomarkers for the development of effective treatment strategies.

Altered metabolic reprogramming, especially increased consumption of glucose and glutamine, has long been recognized as a hallmark of cancer progression [5, 6]. In addition to glucose, cancer cells rely on glutamine as a major nutrient to fulfill their energy requirement and provide substrate for their biosynthetic pathways, including glutathione biosynthesis [7]. Glutamine can be absorbed by cells via specific transporters, such as alanine-serine-cysteine transporter 2 (ASCT2), and then it can be converted into glutamate through glutaminolysis by the key enzyme glutaminase (GLS) [8]. There are two isozymes of glutaminase in the human body: kidney-type glutaminase (GLS1) and liver-type glutaminase (GLS2), encoded by GLS and GLS2 genes, respectively [9]. They differ in structure and tissue distribution and play opposite roles in tumorigenesis. It has been reported that GLS1 has a carcinogenic effect and is regulated by c-Myc, whereas GLS2 exerts a tumor-suppressive effect and is regulated by p53 [10]. Kidney-type glutaminase (KGA) and glutaminase C (GAC) are two alternative splice variants of GLS1 [11]. However, their exact role and expression patterns in HB remain unclear.

Ferroptosis is an iron-dependent mode of regulatory cell death caused by the accumulation of lipid peroxides (LPO), typically characterized by smaller mitochondria, increased membrane density, reduced ridge, and increased levels of intracellular LPO and ROS [12]. Depletion of cytoplasmic GSH decreases the activity of glutathione peroxidase (GPX4) and impairs the elimination of LPO generated during metabolism, leading to iron-dependent lipid peroxidation of polyunsaturated fatty acids (PUFA) on the cell membrane and inducing cell death [13, 14]. In cancer, glutamine is utilized for energy production and GSH biosynthesis, thereby eliminating excessive ROS without triggering ferroptosis [15]. Considering the glutaminolysis of intracellular glutamine provides an important raw material for GSH synthesis and inhibits ferroptosis, the mechanism underlying redox balance maintenance in HB needs further exploration.

Here, we showed that increased glutaminolysis mediated by GLS1 facilitates the development of HB by inhibiting ferroptosis. Furthermore, the KGA isoform is O-GlcNAcylated to maintain its protein stability, which accounts for the majority of glutaminase activity in HB cells. These findings offer a novel therapeutic strategy targeting KGA-mediated glutaminolysis in HB.

## Results

### Activation of glutamine metabolism is needed for the proliferation of HB cells

**Fig. 1:**
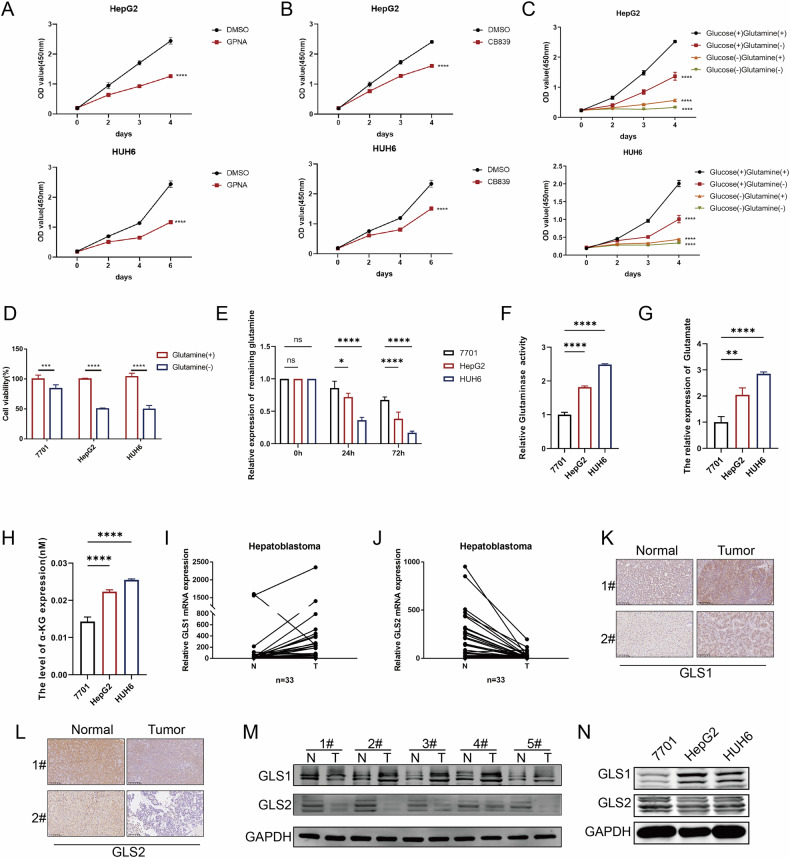
Activation of Gln is needed for the proliferation of HB cells. Using CCK-8 assay the proliferative capacity of HB cells was assessed after treatment with GPNA (250 µM) (**A**) and CB839 (5 µM) (**B**), and glucose (25 mM)/glutamine (4 mM) (**C**). **D** The viability of 7701, HepG2, and HuH6 cells was assessed when cultured in the presence or absence of glutamine. **E** The relative levels of the remaining glutamine in 7701, HepG2, and HuH6 cells were measured after culturing for indicated time points, with an initial glutamine concentration of 4 mM. **F** The relative glutaminase activity of HB cells was compared to that of QSG-7701 hepatocytes. **G** The relative expression of glutamate (%) in HB cell lines was compared to that in QSG-7701 hepatocytes. **H** The level of α-KG (µM) in HB cell lines was compared to that in QSG-7701 hepatocytes. The relative expression levels of GLS1 (**I**) and GLS2 (**J**) in 33 pairs of HB tissues and adjacent tissues were analyzed using qRT-PCR. The expression of GLS1 (**K**) and GLS2 (**L**) proteins in HB tissues and adjacent tissues were detected by IHC, scale bar 100 µm. The expression of GLS1 and GLS2 proteins were measured by WB in 5 pairs of HB tissues and adjacent tissues (**M**) and HB cell lines (**N**), and QSG-7701 hepatocytes; HB hepatoblastoma, CCK-8 cell counting kit-8, IHC immunohistochemistry.

Glucose and glutamine serve as the two most important nutrients needed for the proliferation and growth of cancer cells. However, the exact roles of glutamine in HB have not been investigated so far. We treated HB cells with L-γ-glutamyl-p-nitroanilide (GPNA), an ASCT2 inhibitor, to block glutamine transportation and determine whether HB cells rely on glutamine metabolism. The results indicated that the proliferation of HB cells was significantly decreased after inhibiting glutamine uptake (Fig. 1A). Furthermore, inhibition of GLS1 with CB839 effectively abolished glutaminolysis and dramatically decreased the proliferation of HepG2 and HUH6 cells (Fig. 1B). In addition, by selectively removing either glucose or glutamine from the medium, we confirmed that glutamine serves as a major source of energy and building blocks in cancer cells (Fig. 1C). Compared to normal hepatocyte 7701 cell line, glutamine restriction inhibited the proliferation of HB cells to a greater extent (Fig. 1D) in HepG2 and HUH6 cells. Similarly, we found that glutamine consumption was increased in HepG2 and HUH6 cells compared to 7701 cells (Fig. 1E). Next, we measured glutaminase activity and found that glutaminolysis was significantly enhanced in HB cells compared to 7701 cells (Fig. 1F). Correspondingly, the levels of glutamate and α-ketoglutarate (α-KG) generated from glutaminolysis were also higher in HB cell lines (Fig. 1G, H). Taken together, the results demonstrated that the activation of glutamine metabolism is needed for the proliferation of HB cells.

### Glutamine metabolism facilitates the proliferation of HB cells via GLS1 instead of GLS2

Glutaminase activity was enhanced in HB. We evaluated the expression of two isozymes of glutaminase, including GLS1 and GLS2, in HB. We conducted qRT-PCR in 33 pairs of HB tissues and matched normal tissues, and the results indicated a significant increase in GLS1 and a marked decrease in the expression of GLS2 in HB tissues compared to normal tissues (Fig. 1I, J). Immunohistochemistry (IHC) staining indicated increased expression of GLS1 and decreased expression of GLS2 in HB tissues (Fig. 1K, L and Supplementary Fig. 6L, M). Furthermore, western blotting (WB) validated the expression of GLS1 and GLS2 in 5 paired HB tissues and matched adjacent tissues (Fig. 1M) and HB cell lines HepG2 and HUH6 (Fig. 1N).

**Fig. 2:**
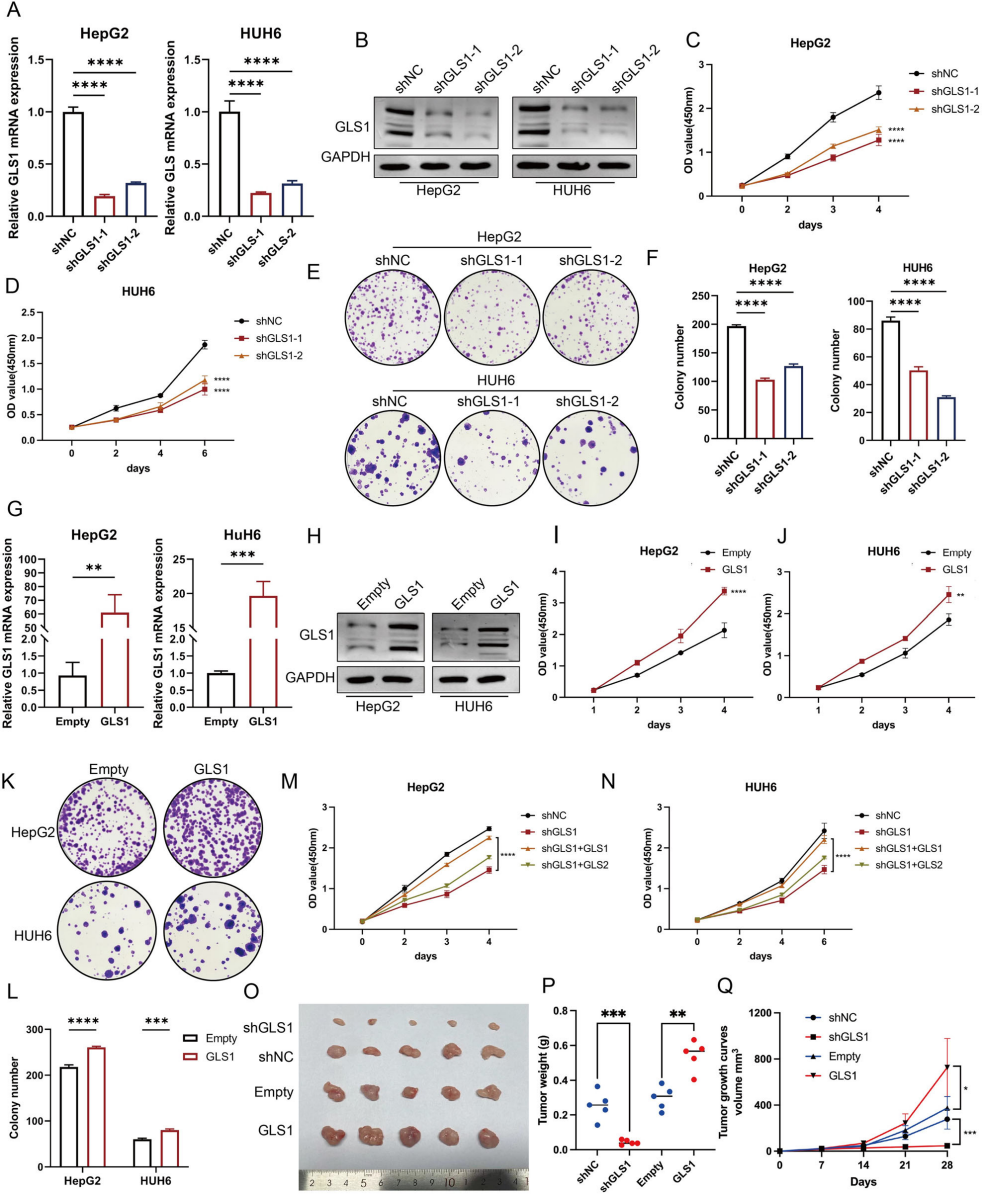
Gln metabolism facilitates the proliferation of HB cells via GLS1 instead of GLS2. The relative mRNA expression of GLS1 (**A**) and protein expression of GLS1 (**B**) were measured with or without GLS1 knockdown. The viability and proliferative activity of HB cells with or without GLS1 knockdown were assessed using CCK-8 assay (**C**, **D**) and colony formation assay (**E**, **F**). The relative mRNA expression of GLS1 (**G**) and the protein expression of GLS1 (**H**) were measured with or without GLS1 overexpression. The viability and proliferative activity of HB cells with GLS1 overexpression were assessed using the CCK8 assay (**I**, **J**) and colony formation assay (**K**, **L**). **M**, **N** CCK8 assays were conducted in HB cells after GLS1 knockdown with or without GLS1 overexpression. **O** Tumors were dissected from five nude mice undergoing subcutaneous injection of HepG2 cell lines. Tumors were dissected after 25 days. **P** Average tumor weight of nude mice (*n* = 5 mice in each group). **P < 0.01, ***P < 0.001, ****P < 0.0001. **Q** Tumor growth curves were created based on tumor volumes (mm^3^) on the specified days.

We generated two independent small hairpin RNAs (shRNAs) to target GLS1 and confirm whether GLS1 stimulates transformed phenotypes in HB cells (Fig. 2A, B). GLS1 knockdown in HepG2 and HUH6 cells significantly reduced cell viability (Fig. 2C, D), accompanied by a decrease in colony formation capacity (Fig. 2E, F). Next, we overexpressed GLS1 to investigate its biological role in HB (Fig. 2G, H). GLS1 overexpression increased cell proliferation and colony formation capacity (Fig. 2I–L). Besides, GLS1-knockdown-mediated inhibition of the transformed phenotypes was reversed by the overexpression of GLS1 instead of GLS2 in HepG2 and HUH6 cells (Fig. 2M, N). Moreover, a xenograft mouse model showed similar circumstances when GLS1 was dysregulated (Fig. 2O–Q). We also generated two shRNA sequences targeting GLS2, and the results indicated that GLS2 had a suppressive effect on HB (Supplementary Fig. 6A–C). Therefore, GLS1, not GLS2, is needed to maintain the transformed phenotype in HB cells.

### Targeting GLS1 expression inhibits the proliferation of HB by inducing ferroptosis

**Fig. 3:**
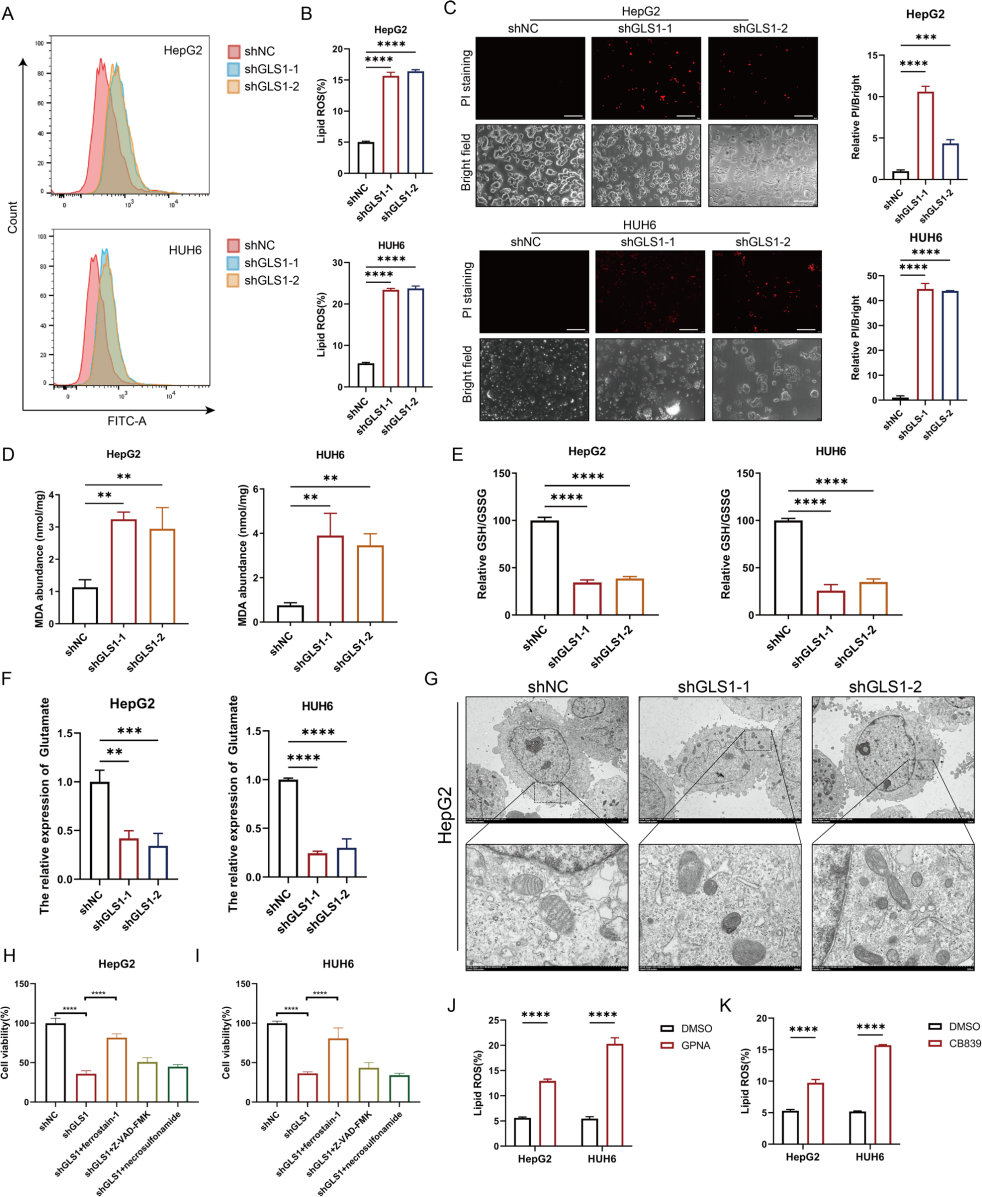
Inhibition of GLS1-mediated glutamine metabolism promoted ferroptosis. **A**, **B** Lipid ROS levels in HB cells with or without GLS1 knockdown were analyzed by flow cytometry using BODIPY C11 staining. **C** PI staining of the HB cells with or without GLS1 knockdown was used to measure cell death, scale bar 200 µm. **D** MDA abundance in HB cells with or without GLS1 knockdown was detected using lipid peroxidation assay kit. **E** The relative GSH/GSSG ratio of HB cells with or without GLS1 knockdown was detected using GSH and GSSG assay kits. **F** The relative expression of glutamate in HB cells with GLS1 knockdown was detected using glutamate assay kits. **G** The morphology of mitochondria in HB cells with or without GLS1 knockdown was measured using an electron microscope, scale bar upper 5 µm, lower ‌500 nm. **H**, **I** The viability of HB cells with or without GLS1 knockdown was measured after treatment with ferrostain-1, VAD-FMK, and necrosulfonamide. Lipid ROS levels of HB cells after treatment with GPNA (250 µM) (**J**) and CB839 (5 µM) (**K**) for 48 h were analyzed using flow cytometry and BODIPY C11 staining.

Since glutamate, generated from glutaminolysis, serves as a crucial precursor for the synthesis of GSH, a key antioxidant against ferroptosis, we investigated the potential role of GLS1 in ferroptosis. As expected, GLS1 knockdown significantly enhanced lipid ROS flux in cell membranes, evidenced by C11-BODIPY staining (Fig. 3A, B). Concurrently, PI staining showed an increase in the number of dead cells (Fig. 3C). The malondialdehyde (MDA) and 4-Hydroxynonenal (4-HNE), which are the final lipid peroxidation products, were upregulated when GLS1 was knocked down in HepG2 and HUH6 cells (Fig. 3D and Supplementary Fig. 6R). Correspondingly, the GSH/GSSG ratio, representing the cellular oxidative balance, was significantly decreased (Fig. 3E). Glutamate was downregulated when GLS1 was knocked down in HepG2 and HUH6 cells (Fig. 3F), while glutamate was slightly downregulated when GLS2 was knocked down in HepG2 and HUH6 cells (Supplementary Fig. 6D). Notably, transmission electron microscopy (TEM) analysis of HepG2 cells indicated distinctive alterations in the mitochondrial ultrastructure of ferroptosis, characterized by swelling, cristae depletion, vacuolation, and increased membrane density (Fig. 3G). We treated the cells separately with ferroptosis inhibitors (ferrostatin-1), apoptosis inhibitors (Z-VAD-FMK), and necrosis inhibitors (necrosulfonamide) to determine whether GLS1 knockdown induces cell apoptosis via ferroptosis or other forms of cell death. The results demonstrated that all the inhibitors could slightly enhanced the cell viability of shNC cells (Supplementary Fig. 6K), by contrast, only ferroptosis inhibitors could markedly reverse GLS1 knockout-mediated death of HepG2 and HUH6 cells (Fig. 3H, I). To validate the effect of glutaminolysis on ferroptosis, we administered the glutamine transporter inhibitor GPNA and the glutaminase activity inhibitor CB839 to block glutamine consumption in HepG2 and HUH6 cells. The results demonstrated a significant increase in lipid ROS (Fig. 3J, K and Supplementary Fig. 1A, E), MDA (Supplementary Fig. 1B, F) and 4-HNE (Supplementary Fig. 6P, Q) levels and a marked decrease in glutaminolysis, accompanied by a substantial inhibition of GSH synthesis (Supplementary Fig. 1C, G), and a significant decrease in glutamate levels (Supplementary Fig. 1D, H) following treatment with GPNA and CB839. Taken together, these results indicated that targeting GLS1 can inhibit HB proliferation by inducing ferroptosis.

### GLS1 regulates the proliferation of HB via KGA isoform instead of GAC

**Fig. 4:**
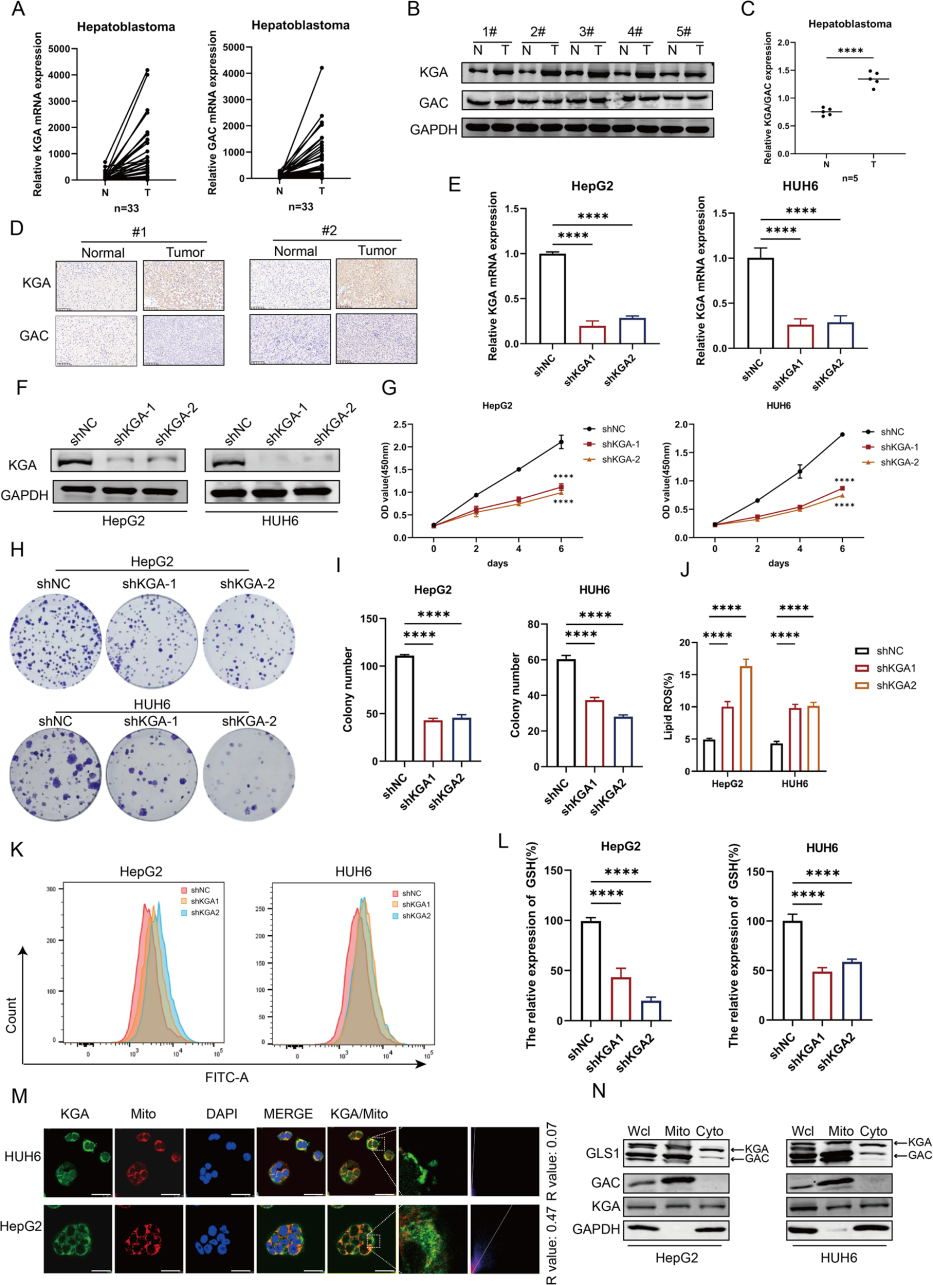
GLS1 regulated the proliferation of HB via the KGA isoform instead of the GAC isoform. **A** The relative expression of KGA and GAC in 33 pairs of HB tissues and adjacent tissues were analyzed using qRT-PCR. **B** The expression of KGA and GAC protein in 5 pairs of HB tissues and adjacent tissues were measured by WB. **C** The relative expression of KGA/GAC was measured in 5 pairs of HB tissues and adjacent tissues. **D** The expression of KGA and GAC proteins in HB tissues and adjacent tissues were detected by IHC, scale bar 100 µm. The mRNA expression of KGA (**E**) and the protein expression of KGA (**F**) were measured in HB cells with or without KGA knockdown. The viability and proliferative activity of HB cells with or without KGA knockdown were measured using CCK-8 assay (**G**) and colony formation assay (**H**, **I**). **J**, **K** Lipid ROS levels in HB cells with or without KGA knockdown were analyzed by flow cytometry with BODIPY C11 staining. **L** The relative expression of GSH in HB cells with or without KGA knockdown was detected using GSH assay kits. **M** The localization of KGA in HB cell lines was measured using confocal microscopy, scale bar 50 µm. **N** The protein expression of KGA and GAC in mitochondria and cytoplasm was measured by WB.

GLS1 possesses two distinct isoforms, namely KGA and GAC, originating from its differential RNA splicing process [7]. Thus, we investigated the differential expression patterns of the two isoforms of GLS1. The transcriptional levels of KGA and GAC were detected by qRT-PCR in 33 paired HB tissues and adjacent normal tissues, and the results revealed a significant increase in the expression levels of both isoforms (Fig. 4A). We verified the differences in protein expression levels between the two isoforms through WB and IHC. The results indicated that KGA was significantly overexpressed at the protein level, while no significant expression difference was observed for GAC (Fig. 4B–D and Supplementary Fig. 6N, O). We generated two independent shRNAs respectively to investigate the biological roles of KGA (Fig. 4E, F). KGA knockdown in HepG2 and HUH6 cells significantly inhibited cell proliferation (Fig. 4G), accompanied by a reduction in colony formation capacity (Fig. 4H, I). Cell counting kit-8 (CCK-8) and colony formation assays revealed that overexpression of KGA (Supplementary Fig. 2A, B) significantly facilitated the proliferation of HB (Supplementary Fig. 2C–F). We subsequently measured the tumorigenic potential of the KGA isoform in a xenograft model employing HepG2 cells. We observed that xenografts of HepG2 cells with KGA knockdown displayed a dramatic decrease in tumor size and weight, whereas KGA overexpression markedly accelerated xenograft tumor growth (Supplementary Fig. 2G–I). We also generated two distinct shRNAs to investigate the biological roles of GAC. GAC knockdown in HepG2 and HUH6 cells led to a modest reduction in cell proliferation. (Supplementary Fig. 6E–G). The results revealed that GLS1 facilitated the proliferation of HB mainly via KGA isoform instead of GAC.

We explored the potential role of KGA in regulating ferroptosis to confirm that GLS1-mediated glutaminolysis relied on KGA isoform in HB cells. C11-BODIPY staining indicated that KGA repression remarkably enhanced lipid ROS (Fig. 4J, K). This was also accompanied by decreased glutamate and GSH levels (Fig. 4L and Supplementary Fig. 2J, K), along with increased MDA levels (Supplementary Fig. 2L, M) and 4-HNE levels (Supplementary Fig. 6S). While KGA overexpression did not affect lipid ROS for HB cells that were not sensitive to ferroptosis (Supplementary Fig. 2N). This was also accompanied by an increase in glutamate and GSH levels (Supplementary Fig. 2O, P) and a decrease in MDA levels (Supplementary Fig. 2Q). Meanwhile, C11-BODIPY staining indicated that the repression of GAC had no effect on lipid ROS levels (Supplementary Fig. 6I, J). Subsequently, due to differences in the functions of KGA and GAC, we investigated their subcellular localization in HB cells. By conducting immunofluorescence assays, we found that the KGA isoform was mainly localized in the cytosol and the subcellular localization of GAC almost completely overlapped with mitochondria (Fig. 4M and Supplementary Fig. 2R). This phenomenon was also validated by mitochondria/cytosol fractionation assay (Fig. 4N).

### O-GlcNAcylation maintained KGA expression level by enhancing its protein stability

**Fig. 5:**
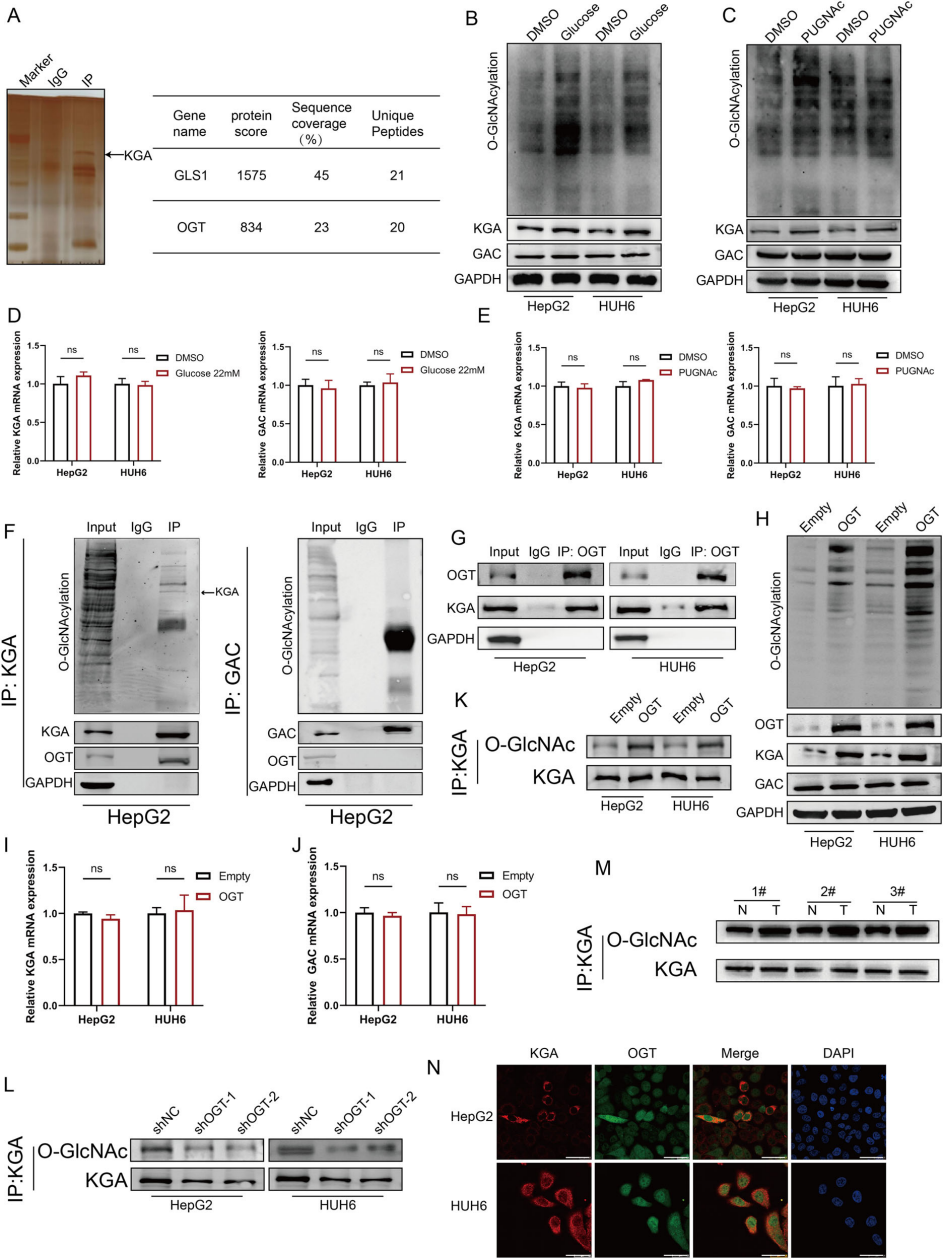
O-GlcNAcylation maintained KGA expression level by enhancing its protein stability. **A** SDS-PAGE separation and silver staining of immunoprecipitated proteins obtained from HepG2 cells overexpressing KGA. The target lane was excised and analyzed using LC–MS/MS analysis. **B** The expression of O-GlcNAcylation, KGA, and GAC proteins in HB cells treated with DMSO or glucose was detected by WB. **C** The expression of O-GlcNAcylation, KGA, and GAC proteins in HB cells treated with DMSO or PUGNAc were detected by WB. **D** The relative expression level of KGA and GAC in HB cells treated with glucose was analyzed by qRT-PCR and compared with that in the DMSO group. **E** The relative expression level of KGA and GAC in HB cells treated with PUGNAc was analyzed by qRT-PCR and compared with that in the DMSO group. **F** Immunoprecipitation (IP) of KGA and GAC with KGA or GAC antibody was conducted to verify the O-GlcNAcylation of KGA or GAC and confirmed the interaction between OGT and KGA or GAC. **G** Immunoprecipitation (IP) of OGT with OGT antibody was conducted to verify the interaction between OGT and KGA. **H** The expression of O-GlcNAcylation, OGT, KGA, and GAC proteins in HB cells overexpressing OGT were measured by WB. The relative expression level of KGA (**I**) and GAC (**J**) in HB cells overexpressing OGT was measured by qRT-PCR. **K** Immunoprecipitation (IP) of KGA with KGA antibody was conducted in HB cells with OGT overexpression to verify the O-GlcNAcylation of KGA. **L** Immunoprecipitation (IP) of KGA with KGA antibody was conducted in HB cells with OGT knockdown to confirm the O-GlcNAcylation of KGA. **M** Immunoprecipitation (IP) of KGA with KGA antibody was conducted in HB and adjacent tissues to confirm the O-GlcNAcylation of KGA. **N** Co-localization of KGA and OGT in HB cells measured by confocal microscopy, scale bar 50 μm.

The results indicated that the difference between the expression of KGA and GAC is mainly reflected at the protein level, rather than at the transcriptional level. We conducted a co-immunoprecipitation and mass spectrometry to identify the potential binding partners of KGA and explore the mechanism behind the high expression of KGA. Surprisingly, mass spectrometry analysis revealed the crucial presence of the enzyme OGT, a pivotal catalyst for O-GlcNAcylation modification, indicating potential O-GlcNAcylation modification of KGA isoform (Fig. 5A). We treated HB cells with glucose and PUGNAc, a well-known stimulator of O-GlcNAcylation, to elevate the entire O-GlcNAcylation levels and validate our hypothesis. The results showed that increased O-GlcNAcylation level significantly enhanced the expression of KGA, whereas the expression of GAC remained unaffected (Fig. 5B, C). Moreover, enhanced O-GlcNAcylation did not affect the mRNA levels of KGA and GAC (Fig. 5D, E). CHX assay indicated that treatment with glucose (Supplementary Fig. 3A–C) and PUGNAc (Supplementary Fig. 3D–F) prolonged the half-life of KGA, suggesting the possibility of post-translational modification in the KGA protein. Furthermore, by analyzing the pulled down of immunoprecipitates (IPs), we detected the presence of O-GlcNAcylated proteins and OGT in the KGA group, whereas no such pattern was observed in the GAC group (Fig. 5F). The presence of endogenous KGA was also observed in the IP specifically targeted and isolated by anti-OGT antibody (Fig. 5G). Similarly, we overexpressed OGT to upregulate the global O-GlcNAcylation, and the results indicated that the KGA isoform was upregulated instead of GAC (Fig. 5H), without affecting the mRNA levels of KGA and GAC (Fig. 5I, J). Correspondingly, overexpression of OGT dramatically increased the O-GlcNAcylation of KGA (Fig. 5K), while OGT knockdown decreased KGA O-GlcNAcylation (Fig. 5L) in HepG2 and HUH6 cells. Furthermore, IP assays validated the increased O-GlcNAcylation levels of KGA in three pairs of HB tissues and their matched adjacent tissues (Fig. 5M). Besides, subcellular colocalization of KGA and OGT was observed by immunofluorescence experiments (Fig. 5N). Taken together, these results indicated that KGA could be O-GlcNAcylated.

### Predicted O-GlcNAcylation of KGA at the Thr563 residue mediates HB ferroptosis via glutaminolysis

**Fig. 6:**
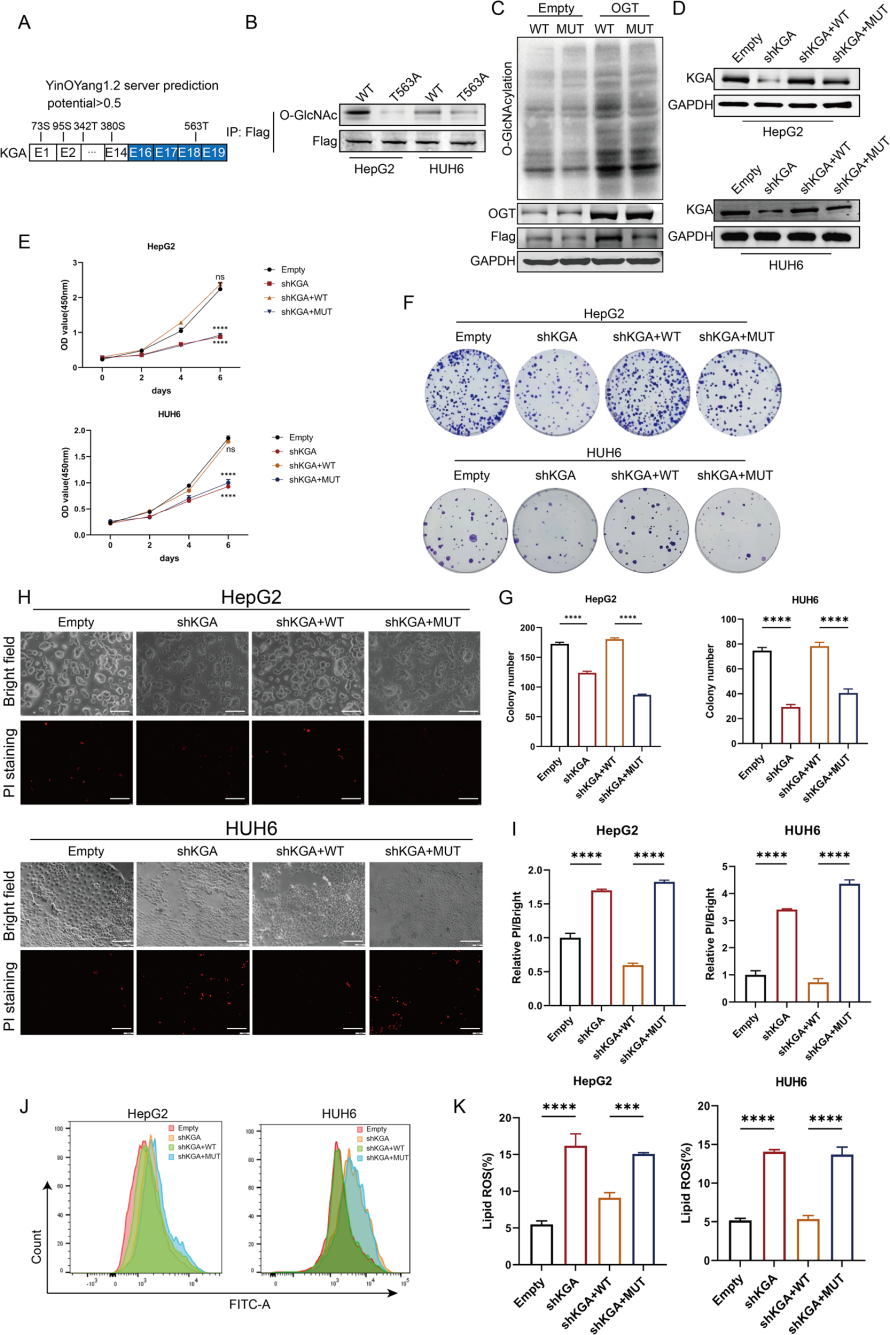
Predicted O-GlcNAcylation of KGA at the Thr563 residue mediates the ferroptosis of HB via glutaminolysis. **A** Prediction of the possible O-GlcNAcylation sites of KGA using the YinOYang 1.2 server online. **B** Immunoprecipitation (IP) of flag with flag antibody was conducted in HB cells with overexpression of the wild-type (WT) or the mutated (T563A) form to confirm the O-GlcNAcylation of flag-tagged KGA. **C** O-GlcNAcylation, OGT, and flag protein levels in HB cells with OGT overexpression were measured by WB. **D** KGA protein levels in shKGA cells overexpressing either the KGA wild-type (WT) or the KGA mutant (T563A) were measured by WB. The viability and proliferative activity of shKGA cells overexpressing either the KGA wild-type (WT) or the KGA mutant (T563A) were assessed using CCK-8 assay (**E**) and colony formation assay (**F**, **G**). **H**, **I** PI staining of shKGA cells overexpressing either the KGA wild-type (WT) or the KGA mutant (T563A) were used to measure cell death, scale bar 200 µm. **J**, **K** Lipid ROS levels of shKGA cells overexpressing either the KGA wild-type (WT) or the KGA mutant (T563A) were analyzed by flow cytometry with BODIPY C11 staining.

We investigated the potential modified sites of KGA. Herein, a bioinformatic website YinOYang version 1.2 (http://www.cbs.dtu.dk/services/YinOYang/) was used to predict the potential O-GlcNAcylated sites of KGA. The prediction sites were Ser73, Ser95, Thr342, Ser380, and Thr563, of which the Thr563 site in exon 18 had the highest predictive score, making it the most likely susceptible to O-GlcNAcylation modification (Fig. 6A). Based on the bioinformatics analysis, we generated all the mutants, including Ser73A (KGA-Ser73A), Ser95A (KGA-Ser95A), Thr342A (KGA-Thr342A), Ser380A (KGA-Ser380A), and Thr563A (KGA-Thr563A), by replacing the indicated amino acid with an alanine (A). As we expected, the mutation of Thr563 in HepG2 and HUH6 cells substantially decreased the O-GlcNAc levels of KGA while other mutation sites exhibited minimal effect on the O-GlcNAc levels of KGA (Fig. 6B and Supplementary Fig. 5A). Furthermore, OGT overexpression notably enhanced the expression of wild-type KGA (KGA-WT), while exhibiting no discernible effect on KGA-Thr563A, referred to as KGA-MUT (Fig. 6C). Similarly, CHX assays revealed that treatment with glucose (Supplementary Fig. 4A, B) and PUGNAc (Supplementary Fig. 4C, D) prolonged the half-life of KGA-WT compared to KGA-Thr543A. These results indicated that Thr563 may be the main putative KGA O-GlcNAc site. Next, we investigated whether the biological function of KGA in HB depends on its O-GlcNAcylated sites. CCK-8 and colony formation assays showed that WT overexpression significantly promoted the proliferation of HB cells, while MUT overexpression did not exert a similar stimulatory effect (Supplementary Fig. 5B–E). Furthermore, after interfering with KGA and concurrently overexpressing either KGA-WT or KGA-MUT (Fig. 6D), WT effectively alleviated cell growth inhibition after KGA interference, whereas MUT failed to demonstrate a similar rescuing effect (Fig. 6E–G). This biological phenotype was validated in the xenograft tumor model (Supplementary Fig. 4E–G). PI staining revealed an increase in the number of dead cells after KGA knockdown, while this phenomenon was reversed by simultaneous overexpression of KGA-WT instead of KGA-MUT (Fig. 6H, I). Consistently, KGA-WT overexpression significantly reduced the elevated levels of lipid ROS triggered by KGA interference, whereas KGA-MUT alone did not offer such an effect (Fig. 6J, K). Comparable outcomes were also observed regarding glutaminase activity, glutamate, GSH, MDA (Supplementary Fig. 4H–K) and 4-HNE (Supplementary Fig. 6T). Thus, KGA-induced malignant behavior of HB primarily depends on its O-GlcNAcylation, and Thr563 may be the putative O-GlcNAc site.

## Discussion

Accumulating evidence indicates that altered metabolism enables cancer cells to produce vast amounts of macromolecules and metabolic intermediates [16–18], which are essential for fueling their rapid growth and division [19, 20]. The metabolism of cancer cells is predominantly composed of carbohydrate metabolism, amino acid metabolism, and lipid metabolism [21, 22]. Within this intricate framework, enzymes and their corresponding substrates can regulate ferroptosis, a newly identified form of iron-dependent cell death, through multiple mechanisms [23]. Hence, targeting ferroptosis has emerged as a novel strategy in the realm of cancer treatment [24].

The close relationship between glutamine metabolism and ferroptosis has been confirmed and garnered significant attention [25, 26]. However, the precise physiological function of glutamine in ferroptosis remains controversial [27]. Gao et al. revealed that glutamine fuels ferroptosis via its distinct metabolic product, α-KG, by engaging in the TCA cycle and leading to lipid peroxidation [28]. Notably, glutaminolysis triggers ferroptosis only after cysteine deprivation [28]. In addition, researchers treated cells with L-γ-glutamyl-p-nitroanilide (GPNA, an inhibitor of the SLC38A1/SLC1A5 complex) or amino-oxyacetic acid (AOA, an inhibitor of pan-transaminases) to hinder glutamine uptake and demonstrated that inhibition of glutaminolysis can block ferroptosis induced by cystine deprivation [25, 26, 28]. Intriguingly, treating cells with high concentrations of extracellular glutamate competitively hinders cystine uptake via the xCT system, resulting in cystine/GSH exhaustion and subsequent accumulation of lipid hydroperoxides [12, 29]; however, intracellular glutamate is a crucial precursor for GSH synthesis. Contrary to previous studies reporting that glutaminolysis can trigger ferroptosis, we proved that glutaminolysis was activated in HB cells to provide essential metabolites, especially glutamate. High intracellular concentrations of glutamate stimulated the production of GSH, and contributed to tumorigenesis by inhibiting ferroptosis.

It has been documented that GLS1 and GLS2, the two enzymes involved in glutaminolysis, exhibit diametrically opposed functions in tumorigenesis [30]. Existing studies have revealed that GLS2, but not GLS1, enhances lipid peroxidation by catalyzing the conversion of glutamine into α-ketoglutarate (α-KG) [31]. This process triggers ferroptosis, which is consistent with the results reported by Gao et al. [28]. In contrast, another study demonstrated that induction of GLS2 can enhance antioxidant capabilities by diminishing intracellular ROS levels through the conversion of glutamine [32]. Nevertheless, despite its antioxidant role, GLS2 overexpression was found to inhibit the proliferation of tumor cells [32]. However, a limited number of studies have focused on elucidating the potential relationship between GLS1 and ferroptosis. Here, we demonstrated that GLS1, not GLS2, inhibits ferroptosis by enhancing the production of glutamate/GSH through glutaminolysis.

Through alternative splicing, GLS1 generates the KGA isoform (comprising exon 1–14, 16–19) alongside its truncated C-terminal counterpart, GAC (consisting of exon 1-15) [33]. The variations in protein structures, specifically differences in their C termini, result in distinct protein expression patterns, functional disparities, and localization differences [34]. By calculating protein genetic ontology, the iLoc-Animal software [35] predicted the subcellular localization of GAC within mitochondria and KGA within the cytoplasm [36]. Moreover, by conducting cell fractionation, WB and IF assays confirmed the localization of KGA/GAC in breast cancer cells (MDA-MB-231 and SKBR3) and prostate tumor cells (PC3 and DU145) [37]. In the present study, we demonstrated that the KGA isoform was mainly localized in the cytosol and GAC was almost completely localized in mitochondria, which is consistent with the existing literature. However, the underlying mechanisms responsible for this discrepancy in subcellular localization have remained elusive to date. Surprisingly, we found that the O-GlcNAcylation modification of KGA occurs in the C-terminal region, which may explain the reasons behind the observed differences in cellular localization. O-GlcNAcylation is a unique type of post-translational modification of proteins, predominantly targeting serine or threonine residues, thereby modulating protein function, stability, and subcellular localization [38]. Numerous oncogenic proteins, including YAP1 [39], c-Myc [40], and AKT [41], have been proven to undergo O-GlcNAcylation, which modulates their stability, activity, and subcellular localization [42].

In conclusion, this study revealed a pivotal role of glutamine metabolism in HB. It is notably activated and orchestrated by GLS1, specifically its kidney-specific isoform KGA, instead of GLS2. Enhanced expression of KGA facilitates the conversion of glutamine into glutamate, effectively suppressing ferroptosis in HB cells and contributing to tumor progression. Notably, KGA undergoes O-GlcNAcylation modification, which amplifies its enzymatic activity and protein abundance. Therefore, KGA is a promising therapeutic target for cancer treatment, presenting a novel and potentially effective strategy for the management of cancer.

## Materials and methods

### Patient and paired tissue samples

In total, 33 pairs of HB (hepatoblastoma) samples and adjacent non-cancerous samples were collected from patients undergoing HB surgery at Shanghai Children’s Medical Center. This study was approved by the Institutional Research Ethics Committee of Shanghai Children’s Medical Center (SCMCIRB-Y2020061). Informed consent was obtained from all participants.

### Cell culture

HepG2, HUH6, and HEK293T cells were purchased from the Cell Bank of the Chinese Academy of Sciences (Shanghai, China), and QSG-7701 from American Type Culture Collection (ATCC) (Maryland, USA). HepG2 cells were grown in Minimum Essential Medium (MEM) (HyClone, Logan, UT, USA). HUH6 and HEK293T cells were cultured in Dulbecco’s Modified Eagle’s Medium (DMEM) (Gibco, Carlsbad, CA, USA). QSG-7701 cells were cultured in RPMI Medium 1640 (Gibco). All Media were supplemented with 10% FBS (F0193, Sigma) and 1% antibiotic (NCM biotech, Suzhou, China). Ferrostatin-1 (2 μM), Z-VAD-FMK (10 μM), and necrosulfonamide (0.5 μM) were used as stimuli in this experiment, all purchased from Selleck Chemicals (Houston, Texas, USA).

### Plasmids and antibodies

Lentiviral-based expressing plasmids for GLS1, OGT, shGLS1-1, shGLS1-2, shGLS2-1, shGLS2-2, shOGT-1, shOGT-2, shGAC-1, shGAC-2, shKGA-1, and shKGA-2 were purchased from Genomeditech (Shanghai, China). Additionally, the KGA-3FLAG, KGA(S73A)-3FLAG, KGA(S95A)-3FLAG, KGA(T342A)-3FLAG, KGA(S380A)-3FLAG, and KGA(T563A)-3FLAG lentiviral-based expressing plasmids were purchased from GeneChem (Shanghai, China). The following antibodies were employed in this study: anti-GLS1 (29519-1-AP, Proteintech), anti-GLS2 (ab113509, Abcam), anti-KGA (20170-1-AP/66265-2-Ig, Proteintech), anti-GAC (19958-1-AP, Proteintech; Invitrogen, MA5-31537), anti-OGT (ab96718, Abcam), anti-O-Linked N-acetylglucosamine (ab2739, Abcam), anti-Flag (ab205606, Abcam; arigo, ARG62342), anti-4-HNE (ab46545, Abcam) and anti-GAPDH antibodies (ab181602, Abcam).

### Western blotting

Cells were collected and lysed in RIPA lysis buffer (P0013B, Beyotime, Jiangsu, China) supplemented with protease and phosphatase inhibitors (P1046, Beyotime) on ice for 10 min. The lysate was centrifuged at 4°C for 10 min, and the supernatant was collected. Protein concentration was determined using the BCA protein quantification kit (23227, Thermo Fisher Scientific, Waltham, MA, USA). Sodium dodecyl sulfate (SDS) loading buffer (P0289, Beyotime) was added to protein samples, and the samples were then boiled at 100°C for 10 min. After electrophoresis on SDS-polyacrylamide gels, the proteins were transferred to nitrocellulose membranes (HATF85R, Millipore, Bedford, MA, USA). The membranes were blocked with 5% BSA (SD6041, Simuwubio, Shanghai, China) for 30 min and incubated with the primary antibodies at 4°C overnight. A fluorescent secondary antibody (Li-COR, USA) was added after washing the membranes three times with PBST. The membranes were then washed with PBST three more times, and the bands on the membranes were detected using Odyssey instruments (Li-COR, USA).

### RNA extraction and quantitative reverse transcription polymerase chain reaction (qRT-PCR) analysis

Total RNA was extracted using TRIzol reagent (15596018, Invitrogen, Waltham, USA). The PrimeScript Reverse Transcription Kit (AG11728, Accurate Biology, Hunan, China) was employed to reverse-transcribe total mRNA into cDNA. The qRT-PCR assay was conducted using the SYBR Green reagent kit (AG11735, Accurate Biology) and QuantStudio 5 (Thermo Fisher Scientific). 18S rRNA was selected as the internal reference gene. The primer sequences used for qRT-PCR assays were as follows:

GLS1 (forward: 5′-GGTGGCCTCAGGTGAAAATAAA-3′, reverse: 5′-AACCTGGGATCAGACGTTCG-3′);

GLS2 (forward: 5′-GCCTGGGTGATTTGCTCTTTT-3′, reverse: 5′-CCTTTAGTGCAGTGGTGAACTT-3′);

KGA (forward: 5′-CATGGATGAAGCACTGCACTTTGG-3′, reverse: 5′-CCTTGAGGTGTGTACTGGACTTGG-3′); GAC (forward: 5′-TTGATCCTCGAAGAGAAGGTGGTG-3′, reverse: 5′-GATGTCCTCATTTGACTCAGGTGAC-3′); 18S (forward: 5′-CAGCCACCCGAGATTGAGCA-3′, reverse: 5′-TAGTAGCGACGGGCGGTGTG-3′). We applied the 2^−ΔΔCT^ method for relative quantitation.

### Lentiviral transduction

HEK293T cells were used for lentiviral packaging. The target plasmid, along with psPAX2 and pMD2G plasmids, were added to HEK293T cells and incubated for 12 h. HB cells were seeded into 6-well plates one day before transfection. Following overnight culture, cell fusion was found to be 30–50% under a microscope. The supernatant was collected after 24 h and 48 h of culture and used along with 2 μg/mL of polybrene (H8761, Solarbio, Beijing, China) to infect HB cells. Infected HB cells were then cultured for an additional 48 h. Finally, 2 mg/L of puromycin was utilized to select stable cell lines.

### Lipid ROS assay

The cells were trypsinized and washed with phosphate-buffered saline (PBS). 1 ml of PBS mixed with 2 μM of BODIPY-C11 (MedChemExpress, NJ, USA) was added to resuspend the cells, which were then dark incubated for 30 min. A CytoFLEX cytometer (Beckman Coulter, California, USA) was used to measure ROS levels, and the CytExpert software (Beckman Coulter) was used to interpret data.

### Co-immunoprecipitation (CO-IP) and LC–MS/MS

Cells in a 10 cm dish were lysed using Western/IP lysis buffer (P0013, Beyotime) containing protease and phosphatase inhibitors. The lysate was collected and incubated on ice for 10 min before centrifugation at 12,000 rpm for 10 min at 4°C. The supernatant was collected, and the protein concentration was determined using the BCA protein quantification method. The protein was mixed with Protein A/G PLUS-Agarose (sc-2003, Santa Cruz, CA, USA), a specific primary antibody (or IgG as a negative control), and IP lysis buffer. This mixture was incubated at 4 °C overnight. The cross-linked beads-protein-antibody complex was washed five times with Western/IP lysis buffer. Subsequently, SDS-PAGE loading buffer was added, and the complexes were boiled at 100 °C for 10 min before Western Blotting and LC-MS/MS analysis.

### Protein stability analysis (CHX)

Cells were treated with DMSO (Sigma, St. Louis, MO, USA), D-glucose (Sigma) or PUGNAc (Sigma) for 12 h. After treatment, cells were incubated with Cycloheximide (CHX, final concentration of 0.1 mg/ml, Sigma) and collected at different time points for lysis and validation by WB.

### Cell proliferation and colony formation assay

The cells seeded in 96-well plates were treated with 90 μl of fresh medium and 10 μl of CCK-8 reagent (C0043, Beyotime). Using a spectrophotometer, the absorbance value was read at 450 nm following incubation at 37 °C for 3 h. The absorbance value was directly proportional to cell viability. Relative cell viability was calculated and normalized using the absorbance values obtained from corresponding dimethyl sulfoxide (DMSO)-treated wells. Next, 1000 cells were added to each well of 12-well plates for colony formation experiments. The cells were cultured in an incubator containing 5% CO2 at 37 °C for 10 days until observing colonies. The colonies were then fixed with methanol and stained with Giemsa.

### Glutaminase, glutamine, glutamate, MDA, α-KG, GSH, and GSSG assay

Glutaminase activity was measured using the glutaminase assay kit (ARG82767, arigo Biolaboratories Corp, Shanghai, China). Glutamine and glutamate levels were measured using the glutamine and glutamate test kit (GLN1-1KT, Sigma, St. Louis, MO, USA). MDA levels were measured using the MDA assay kit (S0131, Beyotime). Alpha-ketoglutarate (α-KG) levels were determined using the alpha-ketoglutarate test kit (ab83431, Abcam, Hong Kong, China). GSH and GSSG levels were quantified using GSH and GSSG assay kits (RK05819, ABclonal, Wuhan, China).

### Immunofluorescence

The slides with cells were prepared in advance by placing them in 24-well plates, and 5000 cells were added to each well. After being cultured overnight, the cells were fixed with 4% paraformaldehyde (PFA) for 15 min. Thereafter, the cells were permeabilized using immunostaining permeabilization solution containing Triton X-100 for 15 min and then blocked with QuickBlock™ blocking buffer (P0260, Beyotime) for 10 min. The primary antibody was added to the cells and incubated at 4°C overnight. The slides were washed three times with PBS, and then dark incubated with the secondary antibody conjugated with a fluorescent dye for 1 h. After being washed three times with PBS, the cells were incubated with DAPI (C1005, Beyotime) for 5 min. Finally, the slides were mounted with an anti-fluorescence quenching agent (P0126, Beyotime) and observed under a Leica SP8 fluorescence microscope.

### Immunohistochemistry (IHC)

The paraffin-embedded sections were deparaffinized using xylene and hydrated using an ethanol gradient. After treatment with 3% methanol-H_2_O_2_, the slices were transferred to a 10 mmol/L sodium citrate solution for antigen retrieval. After blockade with 5% skimmed milk powder solution at 25 °C, the sections were incubated with the primary antibodies, followed by incubation with the secondary antibodies. The sections were then developed with DAB (3,3′-diaminobenzidine tetrahydrochloride) and mounted with neutral gum.

### Xenograft tumor assay

After being approved by Shanghai Children’s Medical Center (SCMC-LAWEC-2021-111), the experiments were conducted strictly following the Guidelines for the Care and Use of Animals for Scientific Research. The xenograft tumor model was established by subcutaneous injection of 1 × 10^7^ cells into 4-week-old male nude mice. Each group comprised five mice, which were individually identified by toe clipping. After 25 days, the mice were euthanized via cervical dislocation, and the tumors were excised and weighed. The tumor volume was calculated using the following formula: tumor volume (mm^3^) = 0.5 × length (mm) × width^2^ (mm^2^).

### PI staining

Propidium Iodide (PI) (C1062M, Beyotime) was used to dark stain the cells plated in 12-well plates at room temperature for 20 min. The cells were then observed under a Leica fluorescence microscope.

### Transmission electron microscope (TEM)

The cells were collected and fixed at 4 °C using a TEM-specific fixative solution (Servicebio, Wuhan, China). Cell masses were embedded in resin. Resin-embedded cells were cut into ultrathin sections with a thickness of 75 nm and mounted on a nickel grid. Finally, a TEM (Hitachi, Tokyo, Japan) was employed to assess the sections. The maximum diameter of the mitochondria was measured using ImageJ software.

### Cell mitochondria isolation

The cell mitochondria isolation kit (C3601, Beyotime) was used to conduct the assay. The cells were digested with trypsin and subsequently collected. The cells were counted after suspension in PBS. Then, 1 ml of mitochondrial separation reagent (with PMSF was added in advance at a concentration of 1 mM) was added to the cells, and the cell suspension was homogenized using a glass homogenizer. Next, the supernatant was collected by centrifugation at 600 × *g* at 4 °C for 10 min. The supernatant was centrifuged again at 11,000 × *g* at 4 °C for 10 min. Mitochondrial proteins were extracted by adding mitochondrial lysate to the precipitate. PMSF was added in advance at a concentration of 1 mM. The resulting mixture was centrifuged at 12,000 × *g* at 4 °C for 10 min. The supernatant and the precipitate were collected separately. The supernatant represents the cytoplasmic protein fraction devoid of mitochondria. The concentration of cytoplasmic protein was measured using the BCA method.

### Statistical analysis

Statistical analyses were conducted using GraphPad Prism 9. Data are presented as mean ± SD. Student’s *t*-tests were used to compare two groups. One-way ANOVA was used to compare more than two groups. A *P*-value < 0.05 was considered significant. *P < 0.05, **P < 0.01, ***P < 0.001, ****P < 0.0001.

## Supplementary information

Supplementary Figure 1

Supplementary Figure 2

Supplementary Figure 3

Supplementary Figure 4

Supplementary Figure 5

Supplementary Figure 6

Supplementary Figure legends

original western blots

qPCR raw data

MS raw data

## Data Availability

All original data and materials will be available by the corresponding author upon request.
