# Supplementary figures and images for: O-GlcNAcylation of glutaminase isoform KGA inhibits ferroptosis through activation of glutaminolysis in hepatoblastoma
Source: Cell Death Discov. 2025 Apr 9;11:160. doi: 10.1038/s41420-025-02464-2 (PMC11982200; doi:10.1038/s41420-025-02464-2)

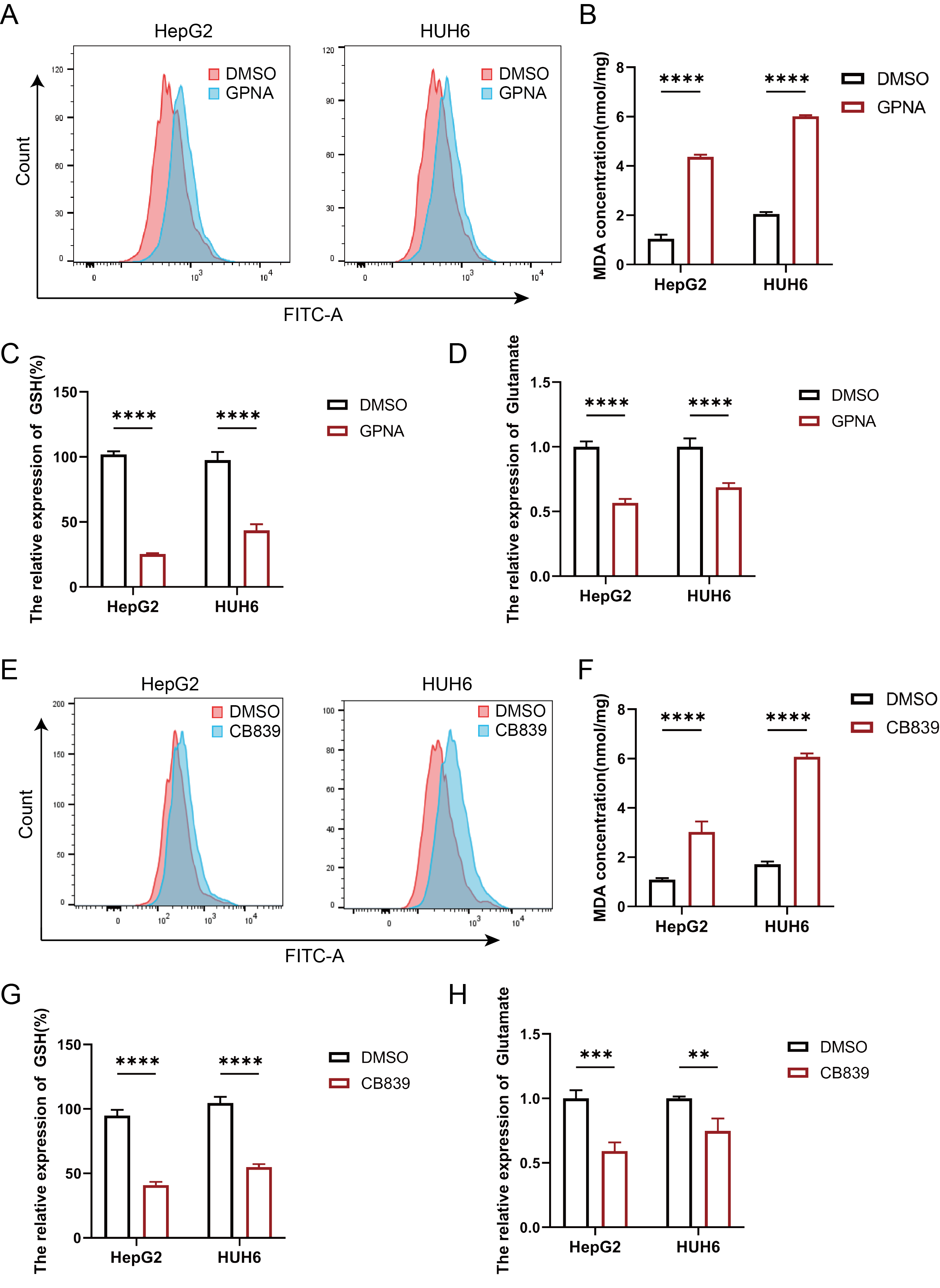

Supplement: Supplementary file 1 — Supplementary Figure 1 [file 41420_2025_2464_MOESM1_ESM.tif]

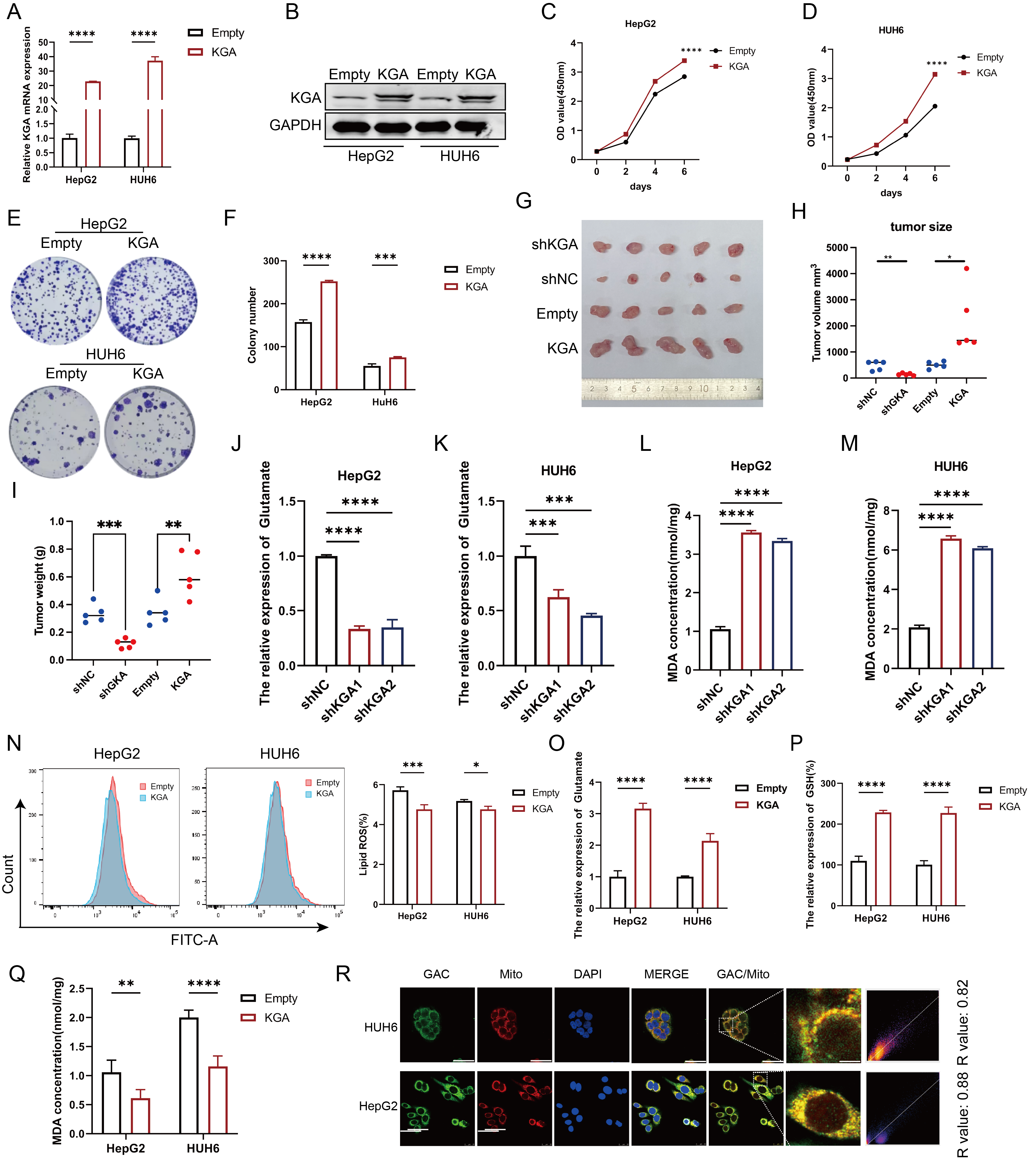

Supplement: Supplementary file 2 — Supplementary Figure 2 [file 41420_2025_2464_MOESM2_ESM.tif]

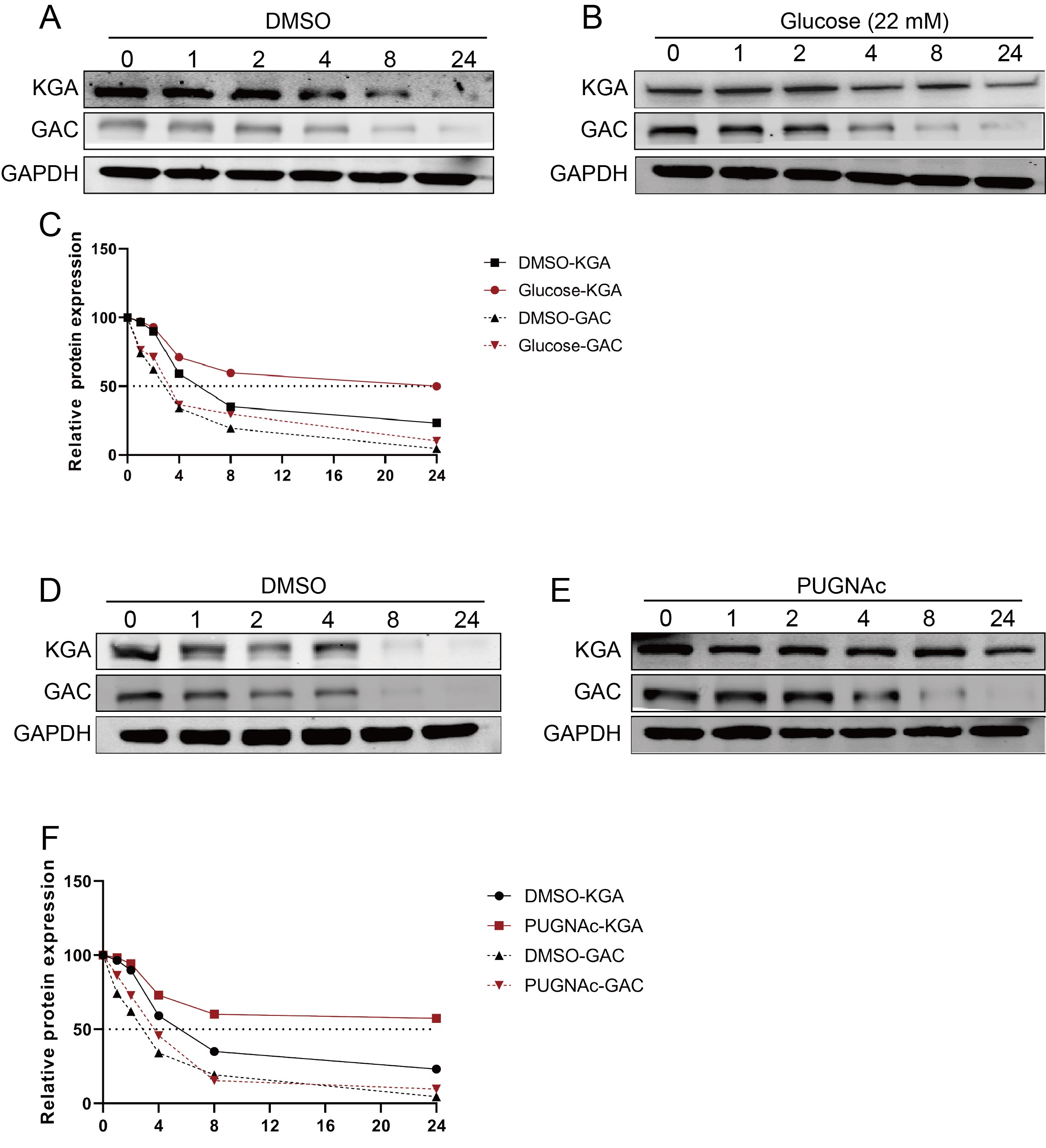

Supplement: Supplementary file 3 — Supplementary Figure 3 [file 41420_2025_2464_MOESM3_ESM.tif]

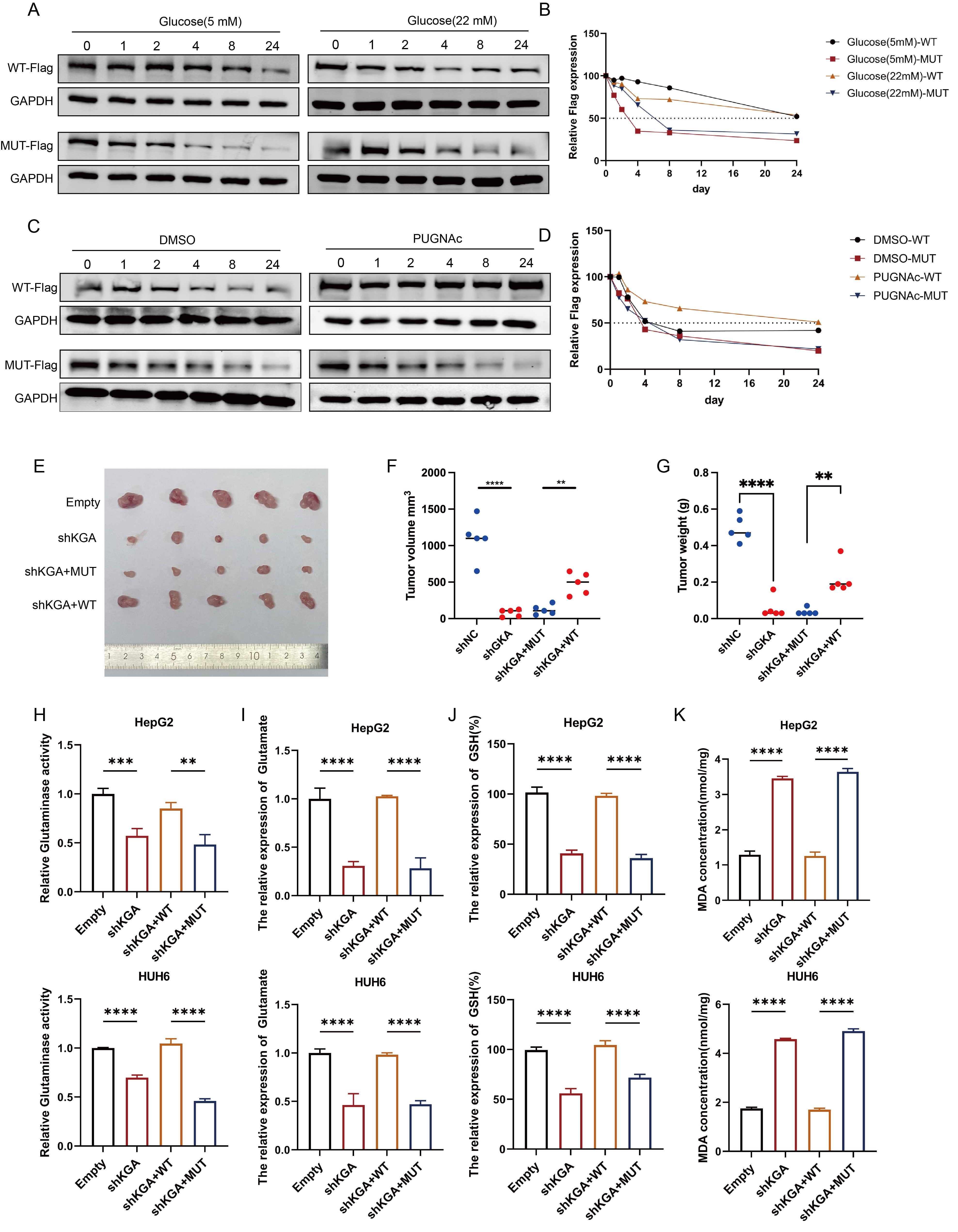

Supplement: Supplementary file 4 — Supplementary Figure 4 [file 41420_2025_2464_MOESM4_ESM.tif]

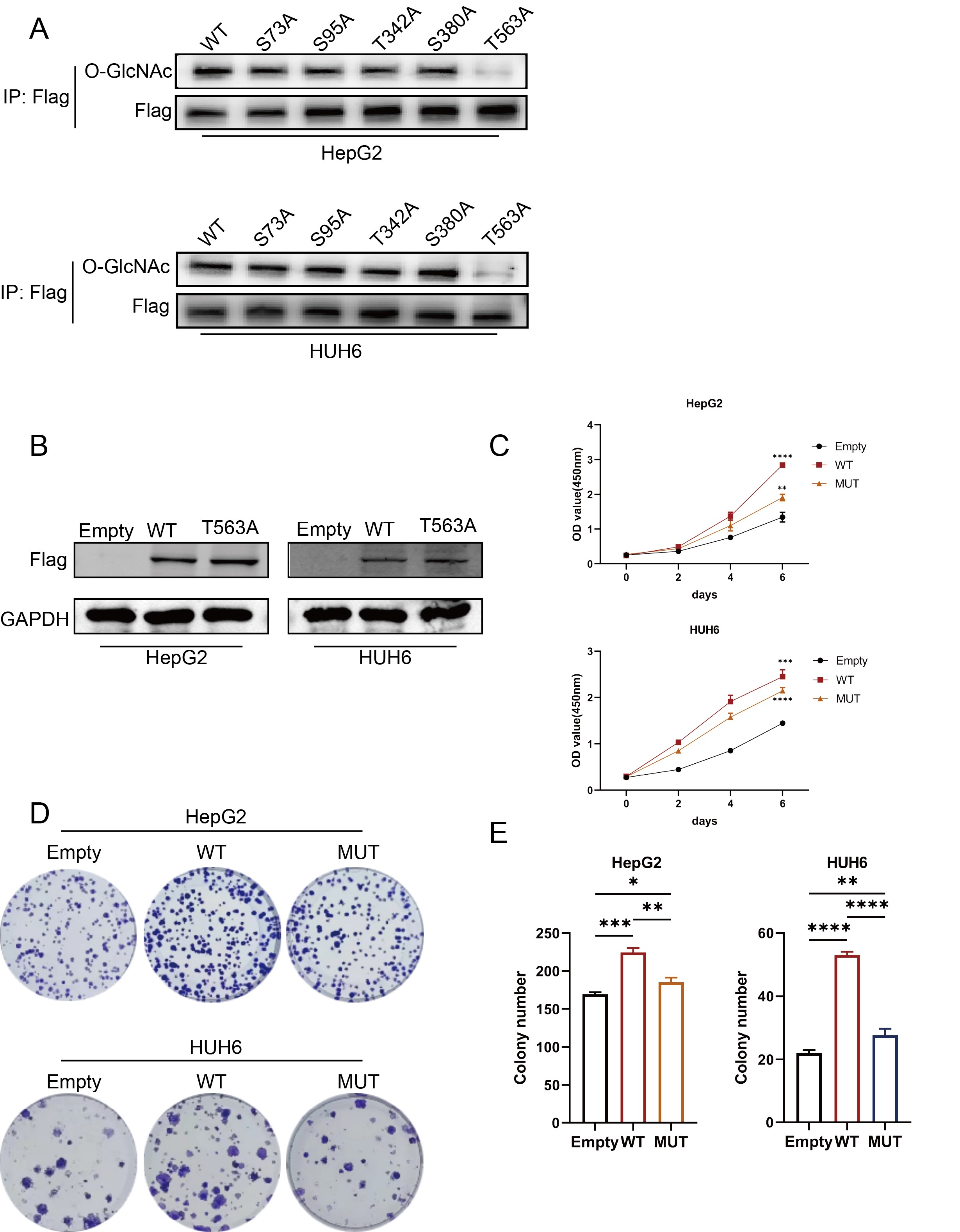

Supplement: Supplementary file 5 — Supplementary Figure 5 [file 41420_2025_2464_MOESM5_ESM.tif]

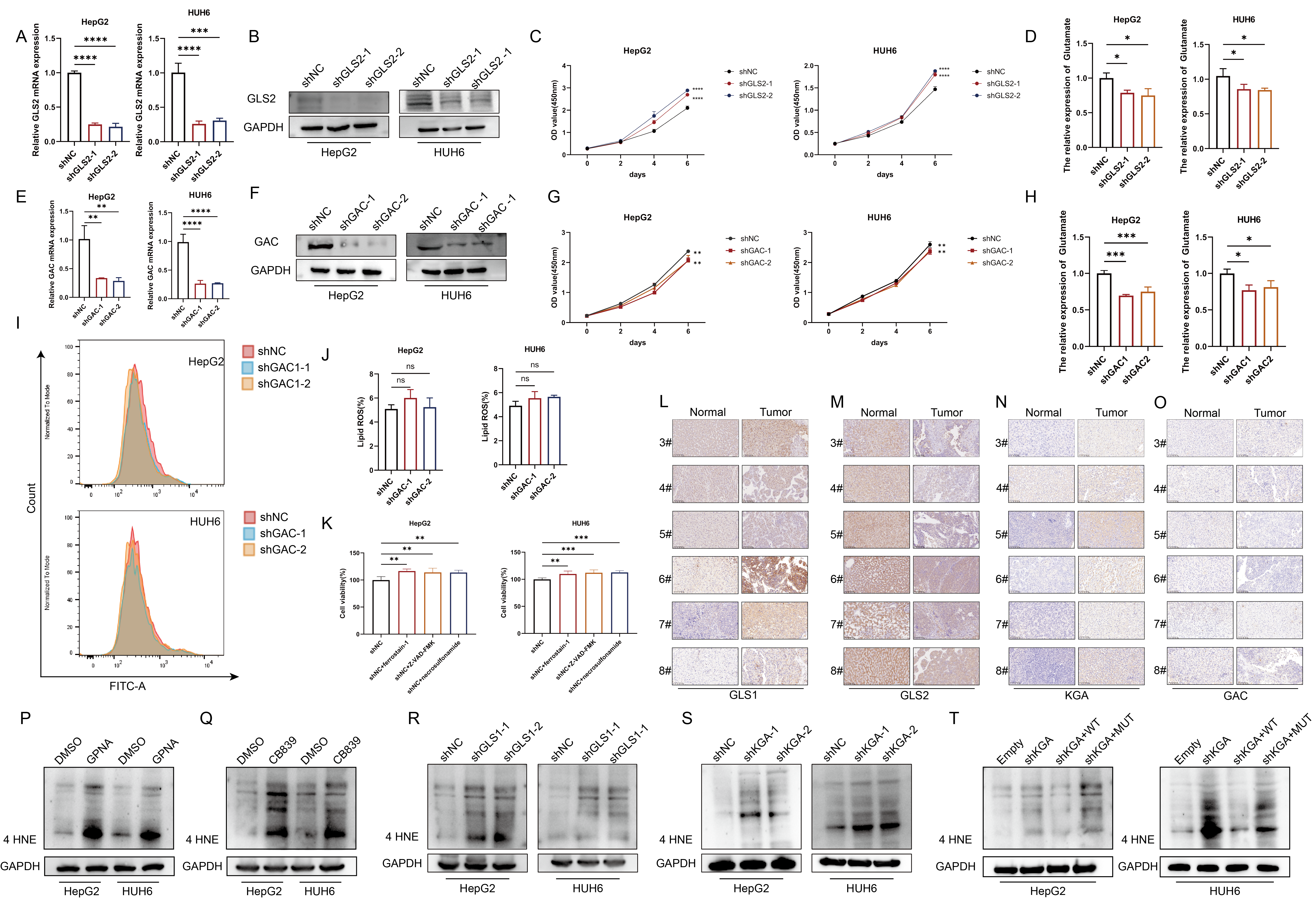

Supplement: Supplementary file 6 — Supplementary Figure 6 [file 41420_2025_2464_MOESM6_ESM.tif]
