# Supplementary material for: O-GlcNAcylation of glutaminase isoform KGA inhibits ferroptosis through activation of glutaminolysis in hepatoblastoma
Source: Cell Death Discov. 2025 Apr 9;11:160. doi: 10.1038/s41420-025-02464-2 (PMC11982200; doi:10.1038/s41420-025-02464-2)
Supplement: Supplementary file 7 — Supplementary Figure legends [file 41420_2025_2464_MOESM7_ESM.docx]

**Supplementary figure 1**

A Lipid ROS levels of HB cells after treatment with GPNA (250µM) for 48h were analyzed by flow cytometry using BODIPY C11 staining. B, C Ferroptotic events were evaluated in HB cells treated with GPNA (250µM) for 48h: MDA concentration (B), and relative GSH levels (C). D The relative expression of glutamate in HB cells after treatment with GPNA (250µM) for 48h was detected using glutamate assay kits. E Lipid ROS levels of HB cells after treatment with CB839 (5µM) for 48h were analyzed by flow cytometry using BODIPY C11 staining. F, G Ferroptotic events were evaluated in HB cells treated with CB839 (5µM) for 48h: MDA concentration (F), and relative GSH levels (G). H The relative expression of glutamate in HB cells after treatment with CB839 (5µM) for 48h was detected using glutamate assay kits.

**Supplementary figure 2**

A, B The relative mRNA expression of KGA (A) and the protein expression of KGA (B) were measured in HB cells with or without KGA overexpression. C-F The viability and proliferative activity of HB cells with or without KGA overexpression were assessed using the CCK8 assay (C, D) and colony formation assay (E, F). G Tumors were dissected from five nude mice undergoing subcutaneous injection of HepG2 cell lines. Tumors were dissected after 25 days. H Average tumor weight of nude mice (n=5 mice in each group). I Tumors growth curves were created based on tumor volumes (mm^3^) on the specified days. J, K The relative expresssion of glutamate in HB cells with KGA knockdown was detected using glutamate assay kits. L, M MDA concentration of HB cells with or without KGA knockdown was detected using lipid peroxidation assay kit. N Lipid ROS levels of HB cells with KGA overexpression were analyzed by flow cytometry using BODIPY C11 staining. O The relative expresssion of glutamate in HB cells with KGA overexpression was detected using glutamate assay kits. P The relative expression of GSH of HB cells with KGA overexpression was detected using GSH assay kits. Q MDA concentration of the HB cells with or without KGA overexpression was detected using lipid peroxidation assay kit. R The localization of GAC in HB cell lines was measured using confocal microscopy, scale bar 50 µm.

**Supplementary figure 3**

1. C The decay rates of KGA and GAC proteins in HB cells were detected via Western blot (A, B) and quantitative analysis (C) at indicated time points in the presence of DMSO or glucose(22mM), both under the condition of cycloheximide (CHX) treatment at a concentration of 100 µM. D-F The decay rates of KGA and GAC proteins in HB cells were detected via Western blot (D, E) and quantitative analysis (F) at indicated time points in the presence of DMSO or PUGNAc (12.5µM), both under the condition of cycloheximide (CHX) treatment at a concentration of 100 µM.

**Supplementary figure 4**

A, B The decay rate of flag protein in HB cells overexpressing either the wild type (WT) or the T563A mutant was detected through Western Blot (A) and quantitative analysis (B) at indicated time points in the presence of glucose (5mM or 22mM) under the treatment of cycloheximide (CHX) (100 µM). C, D The decay rate of flag protein in HB cells overexpressing either the wild type (WT) or the T563A mutant was detected through Western Blot (C) and quantitative analysis (D) at indicated time points in the presence of DMSO or PUGNAc (12.5µM) under the treatment of cycloheximide (CHX) (100 µM). E Tumors were dissected from five nude mice undergoing subcutaneous injection of HepG2 cell lines. Tumors were dissected after 25 days. F Average tumor weight of nude mice (n=5 mice in each group). **, P<0.01, ***, P<0.001, ****, P<0.0001. G Tumors growth curves were created base on tumor volumes (mm^3^) on the specified days. H The relative glutaminase activity of shKGA cells overexpressing either the KGA wild-type (WT) or the KGA mutant (T563A )was detected using glutaminase assay kits. I The relative expression of glutamate in shKGA cells overexpressing either the KGA wild-type (WT) or the KGA mutant (T563A) was detected using glutamate assay kits. J, K Ferroptotic events were evaluated in shKGA cells overexpressing either the KGA wild-type (WT) or the KGA mutant (T563A): relative GSH levels (J), and MDA concentration (K).

**Supplementary figure 5**

A Immunoprecipitation (IP) of flag with flag antibody was conducted in HB cells overexpressing either the wild type (WT) or mutant variants (S73A, S95A, T342A, S380A, T563A) to verify the O-GlcNAcylation of flag-tagged KGA. B Flag protein levels in HB cells overexpressing either the KGA wild-type (WT) or the KGA mutant (T563A) were measured by Western blot. C-E The viability and proliferative activity of HB cells overexpressing either the KGA wild-type (WT) or the KGA mutant (T563A) were assessed using the CCK8 assay (C) and colony formation assay (D, E).

**Supplementary figure 6**

A, B The relative mRNA expression of GLS2 (A) and protein expression of GLS2 (B) were measured with or without GLS2 knockdown. C The viability of HB cells with or without GLS2 knockdown was assessed using CCK-8 assay. D The relative expression of glutamate in HB cells with GLS2 knockdown was detected using glutamate assay kits. E, F The relative mRNA expression of GAC (E) and protein expression of GAC (F) were measured with or without GAC knockdown. G The viability of HB cells with or without GAC knockdown was assessed using CCK-8 assay. H The relative expression of glutamate in HB cells with GAC knockdown was detected using glutamate assay kits. I, J Lipid ROS levels in HB cells with or without GAC knockdown were analyzed by flow cytometry using BODIPY C11 staining. K The viability of shNC HB cells was measured after treatment with ferrostain-1, VAD-FMK, and necrosulfonamide. L-O The expression of GLS1 (L), GLS2 (M), KGA(N) and GAC(O) proteins in 6 paired of HB tissues and adjacent tissues were detected by IHC, scale bar 100 µm. P 4-HNE protein levels in HB cells treated with GPNA (250µM) for 48h. Q 4-HNE protein levels in HB cells treated with CB839 (5µM) for 48h. R 4-HNE protein levels in HB cells with or without GLS1 knockdown. S 4-HNE protein levels in HB cells with or without KGA knockdown. T 4-HNE protein levels in shKGA cells overexpressing either the KGA wild-type (WT) or the KGA mutant (T563A) were measured by WB.
