# Supplementary material for: O-GlcNAcylation of glutaminase isoform KGA inhibits ferroptosis through activation of glutaminolysis in hepatoblastoma
Source: Cell Death Discov. 2025 Apr 9;11:160. doi: 10.1038/s41420-025-02464-2 (PMC11982200; doi:10.1038/s41420-025-02464-2)
Supplement: Supplementary file 8 — original western blots [file 41420_2025_2464_MOESM8_ESM.pptx]

## Slide 1
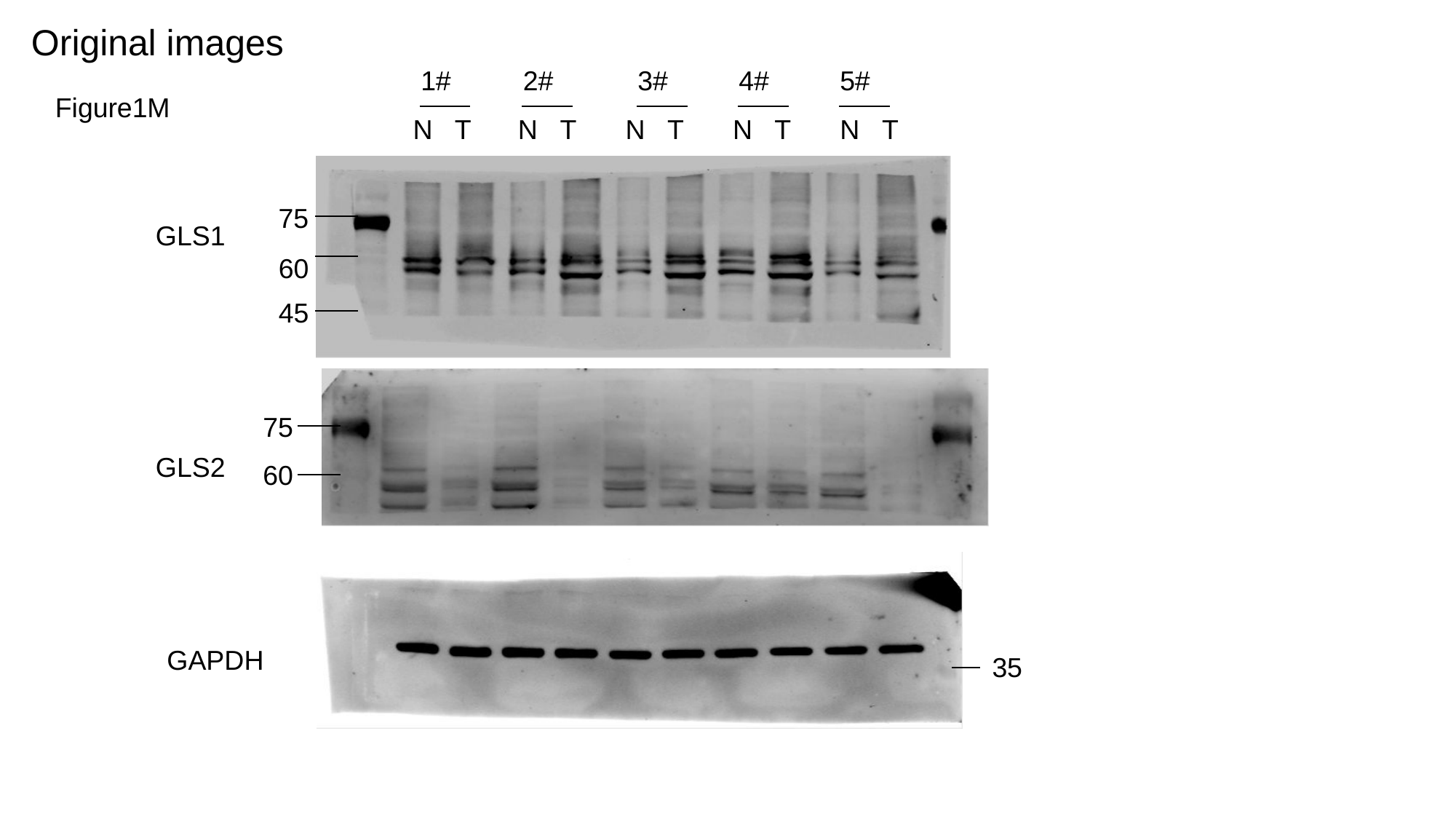

Original images
1#
2#
3#
4#
5#
Figure1M
N
T
N
T
N
T
N
T
N
T
75
GLS1
60
45
75
GLS2
60
GAPDH
35

## Slide 2
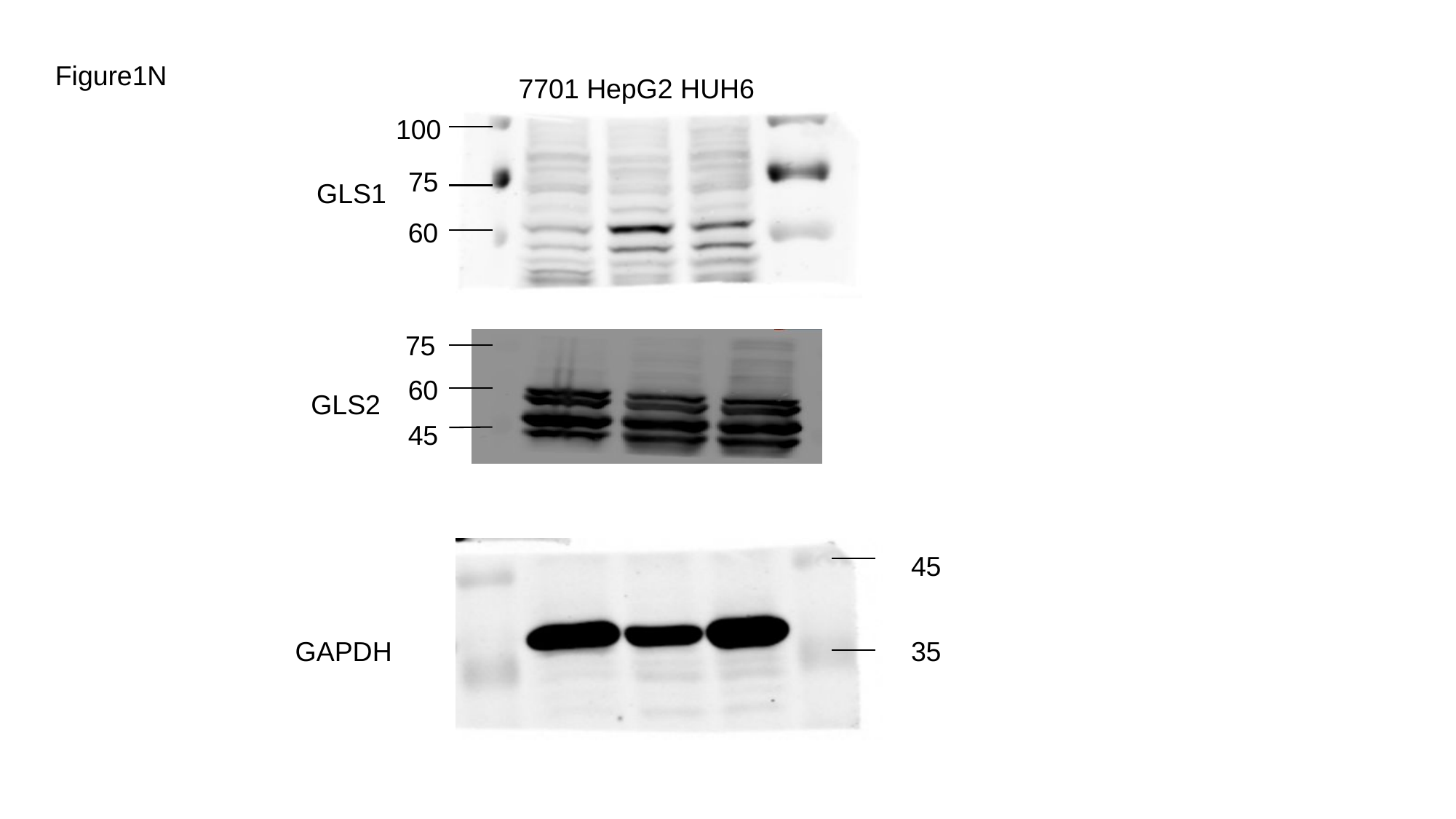

Figure1N
7701 HepG2 HUH6
100
75
GLS1
60
75
60
GLS2
45
45
GAPDH
35

## Slide 3
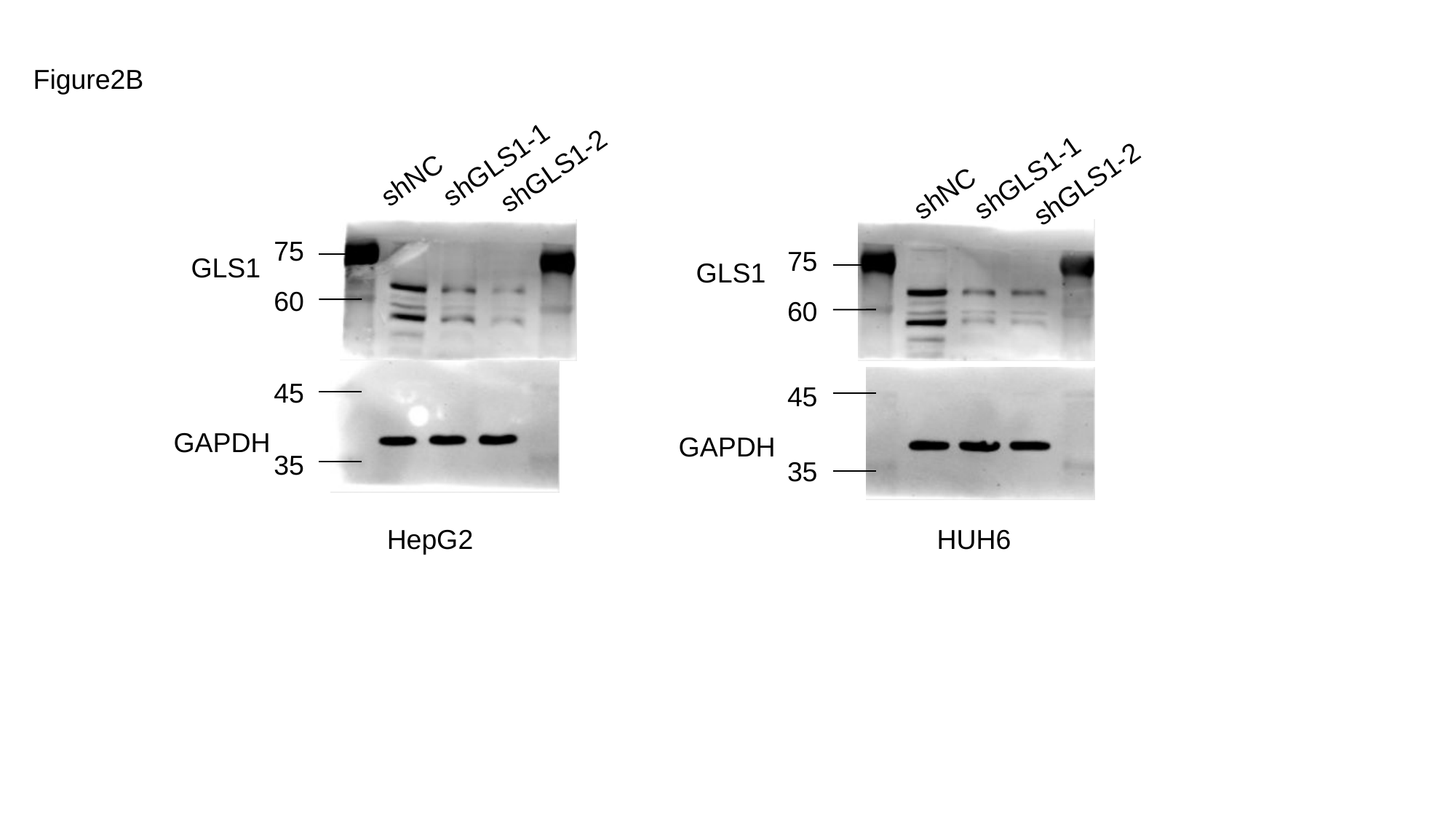

Figure2B
shGLS1-1
shGLS1-2
shGLS1-1
shNC
shGLS1-2
shNC
75
75
GLS1
GLS1
60
60
45
45
GAPDH
GAPDH
35
35
HepG2
HUH6

## Slide 4
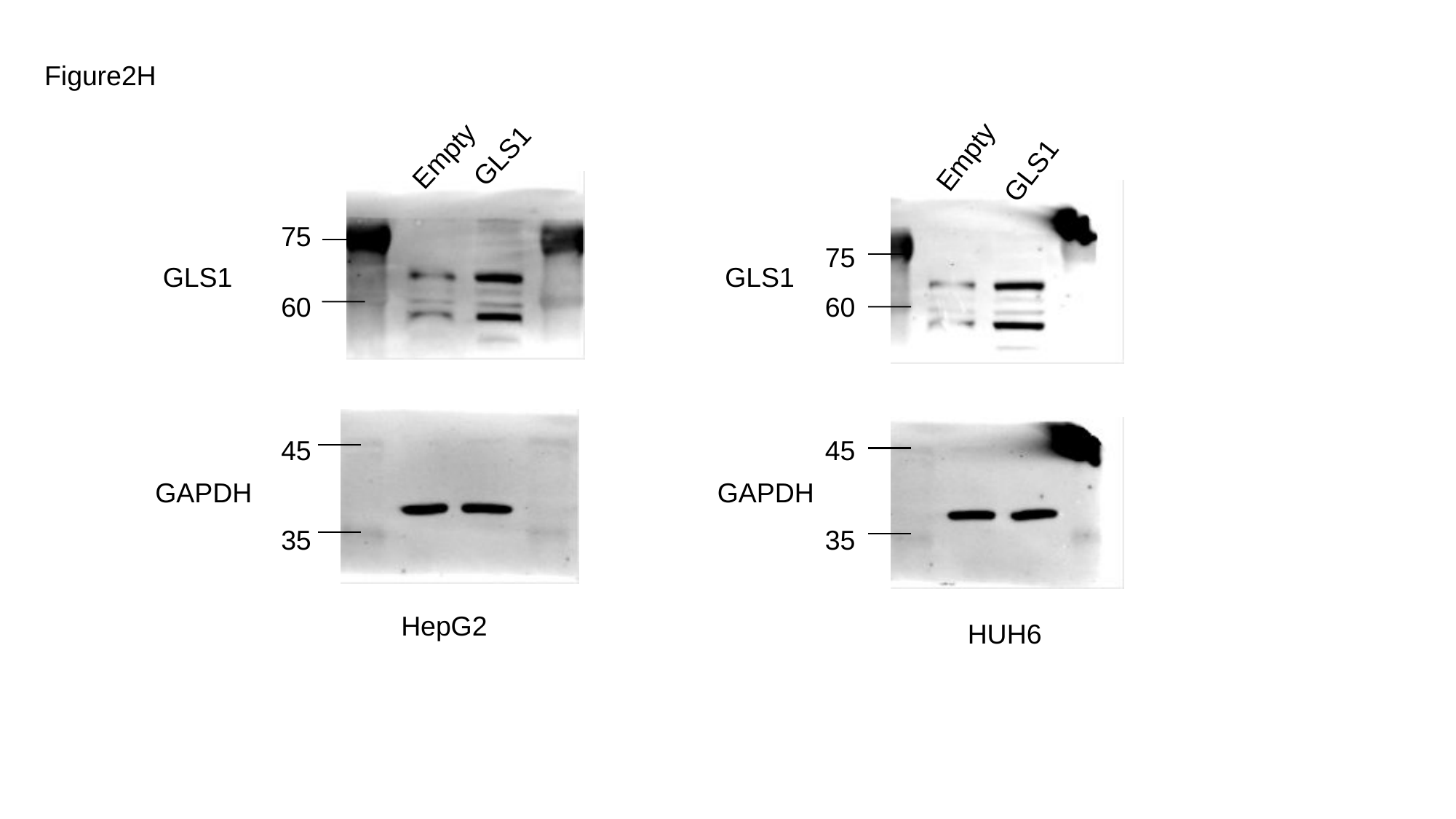

Figure2H
GLS1
Empty
Empty
GLS1
75
75
GLS1
GLS1
60
60
45
45
GAPDH
GAPDH
35
35
HepG2
HUH6

## Slide 5
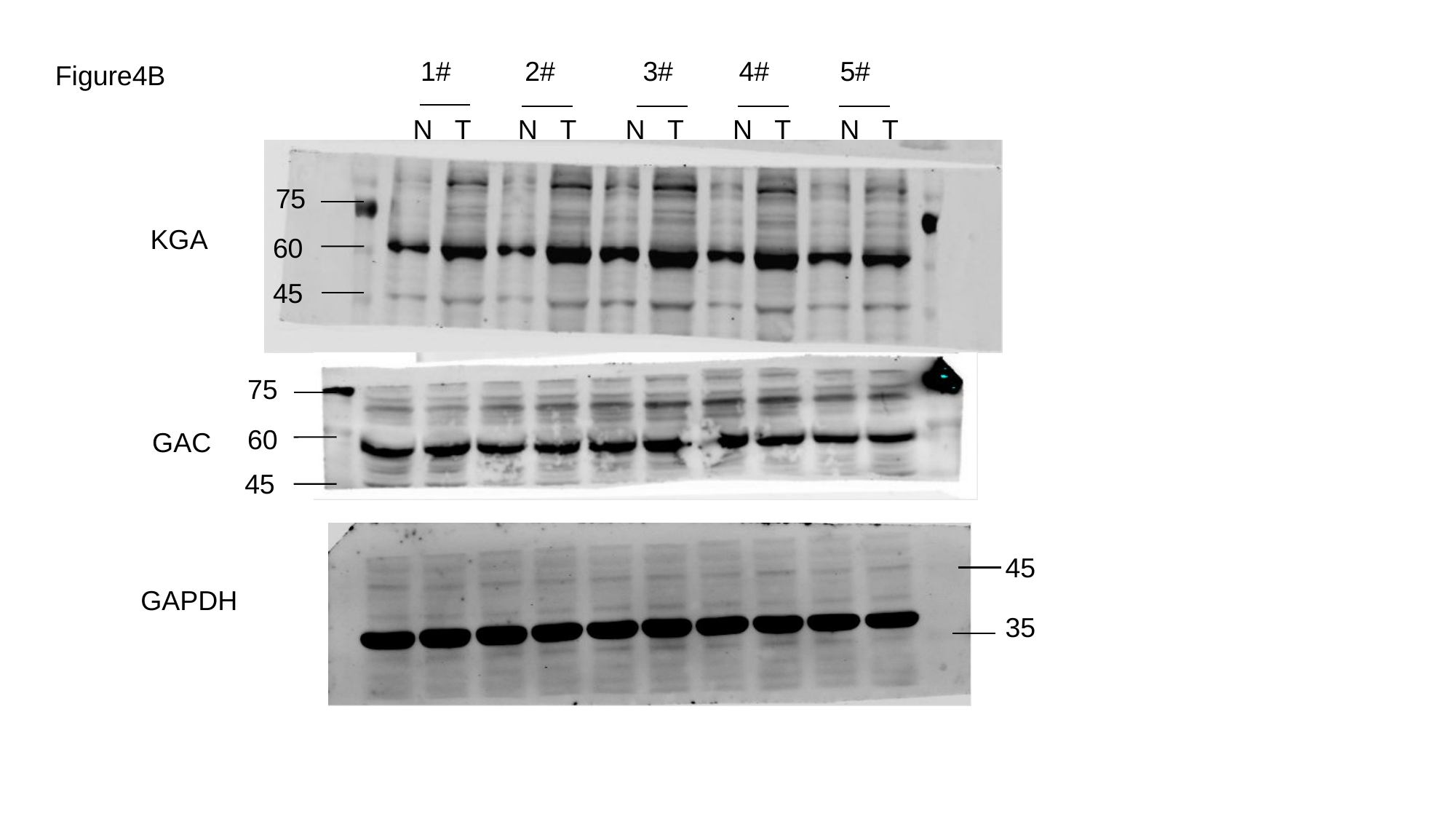

1#
2#
3#
4#
5#
Figure4B
N
T
N
T
N
T
N
T
N
T
75
KGA
60
45
75
60
GAC
45
45
GAPDH
35

## Slide 6
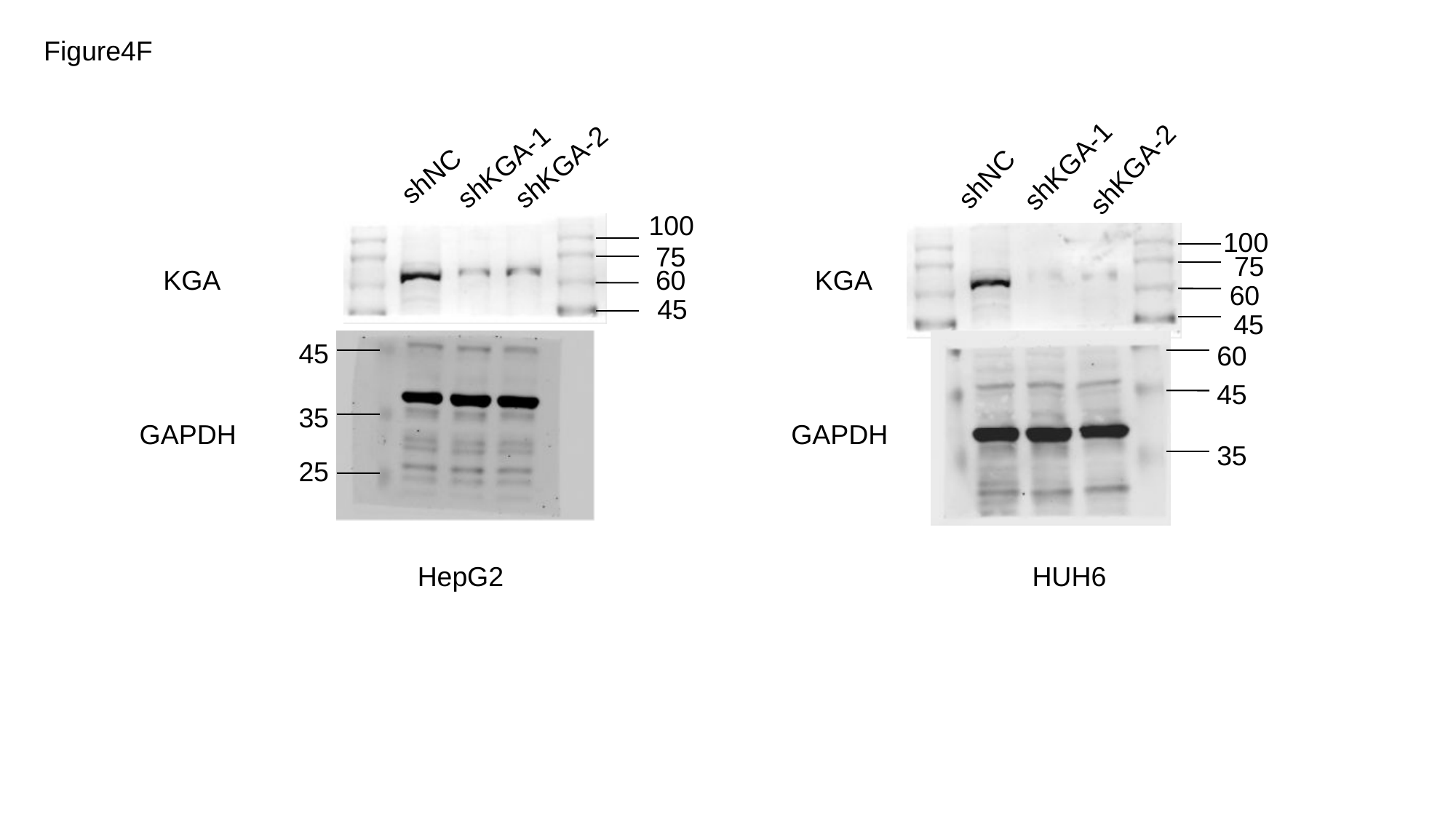

Figure4F
shKGA-1
shKGA-1
shKGA-2
shKGA-2
shNC
shNC
100
100
75
75
60
KGA
KGA
60
45
45
45
60
45
35
GAPDH
GAPDH
35
25
HepG2
HUH6

## Slide 7
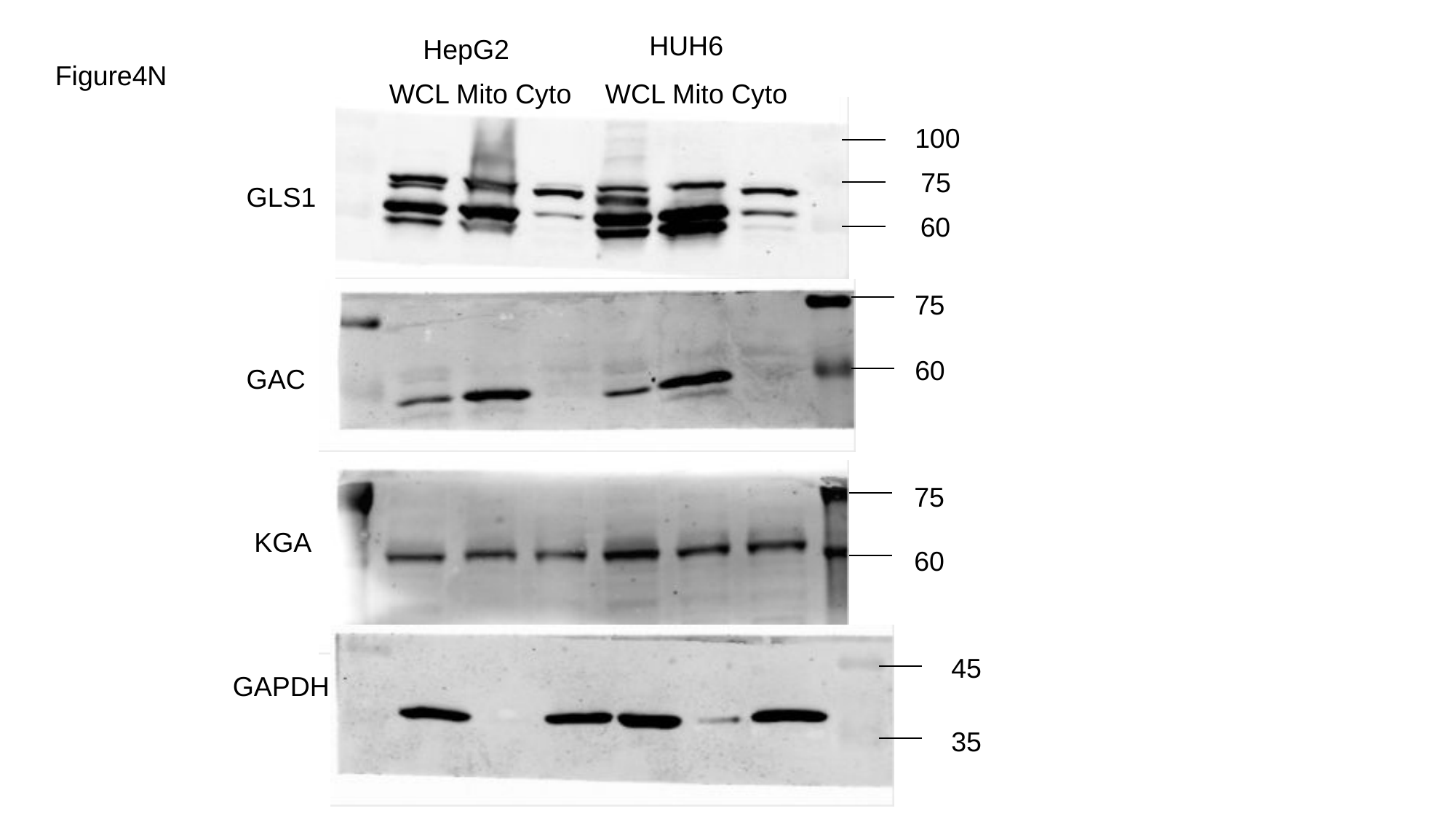

HUH6
HepG2
Figure4N
WCL Mito Cyto
WCL Mito Cyto
100
75
GLS1
60
75
60
GAC
75
KGA
60
45
GAPDH
35

## Slide 8
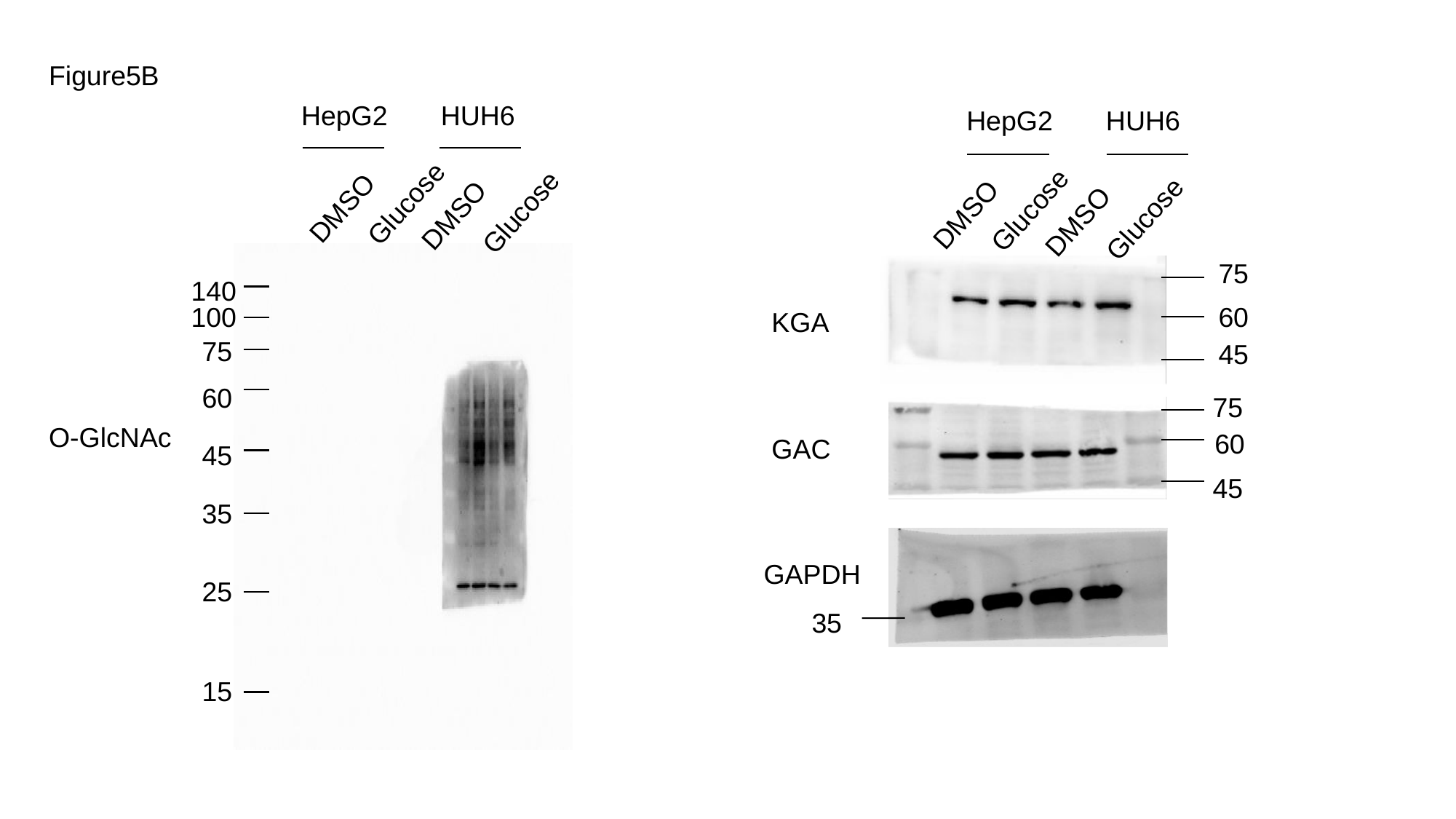

Figure5B
HepG2 HUH6
HepG2 HUH6
 Glucose
DMSO
 Glucose
DMSO
DMSO
 Glucose
DMSO
 Glucose
75
140
100
60
KGA
75
45
60
75
O-GlcNAc
60
GAC
45
45
35
GAPDH
25
35
15

## Slide 9
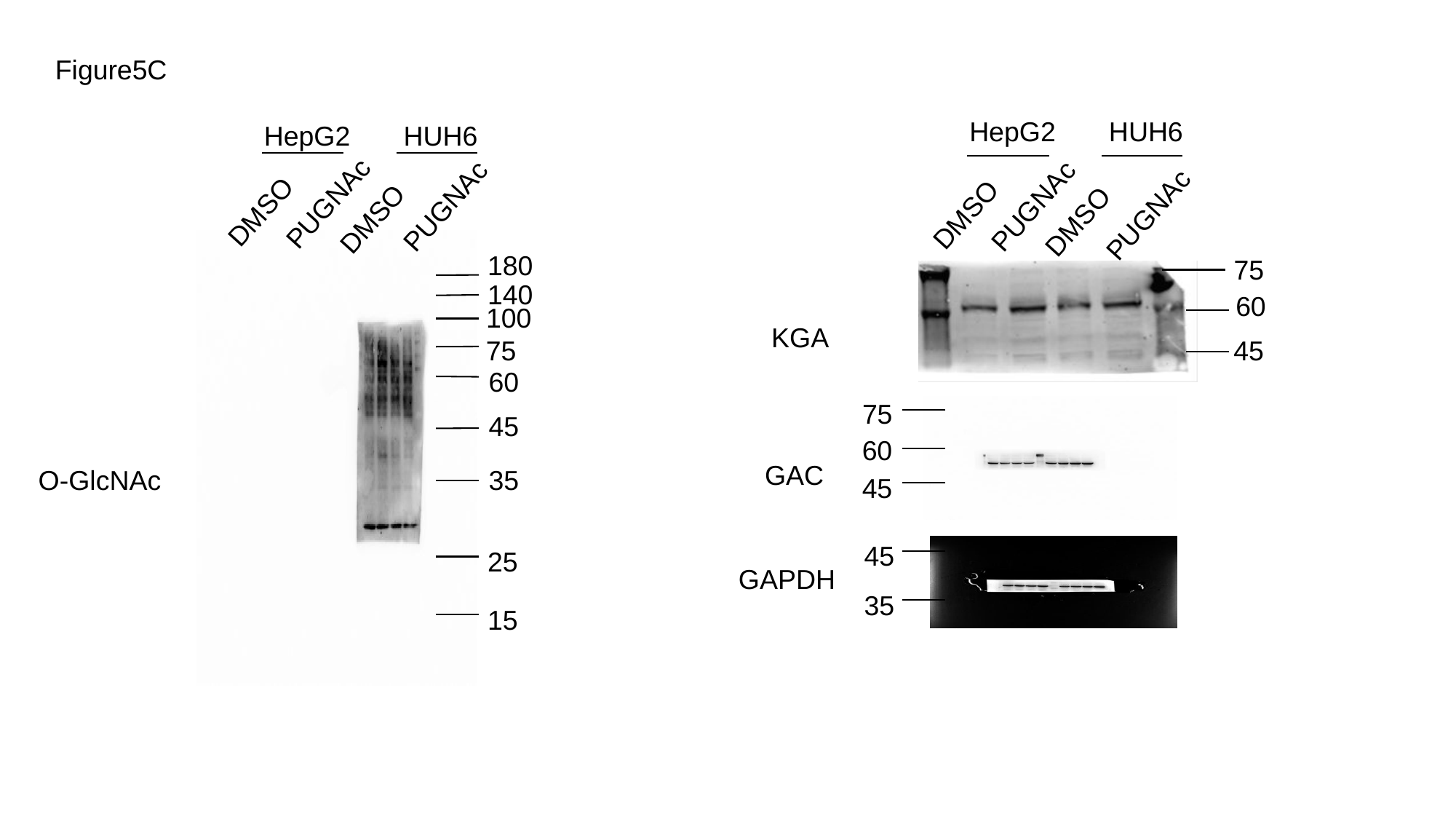

Figure5C
HepG2 HUH6
HepG2 HUH6
 PUGNAc
 PUGNAc
 PUGNAc
 PUGNAc
DMSO
DMSO
DMSO
DMSO
180
75
140
60
100
KGA
75
45
60
75
45
60
GAC
O-GlcNAc
35
45
45
25
GAPDH
35
15

## Slide 10
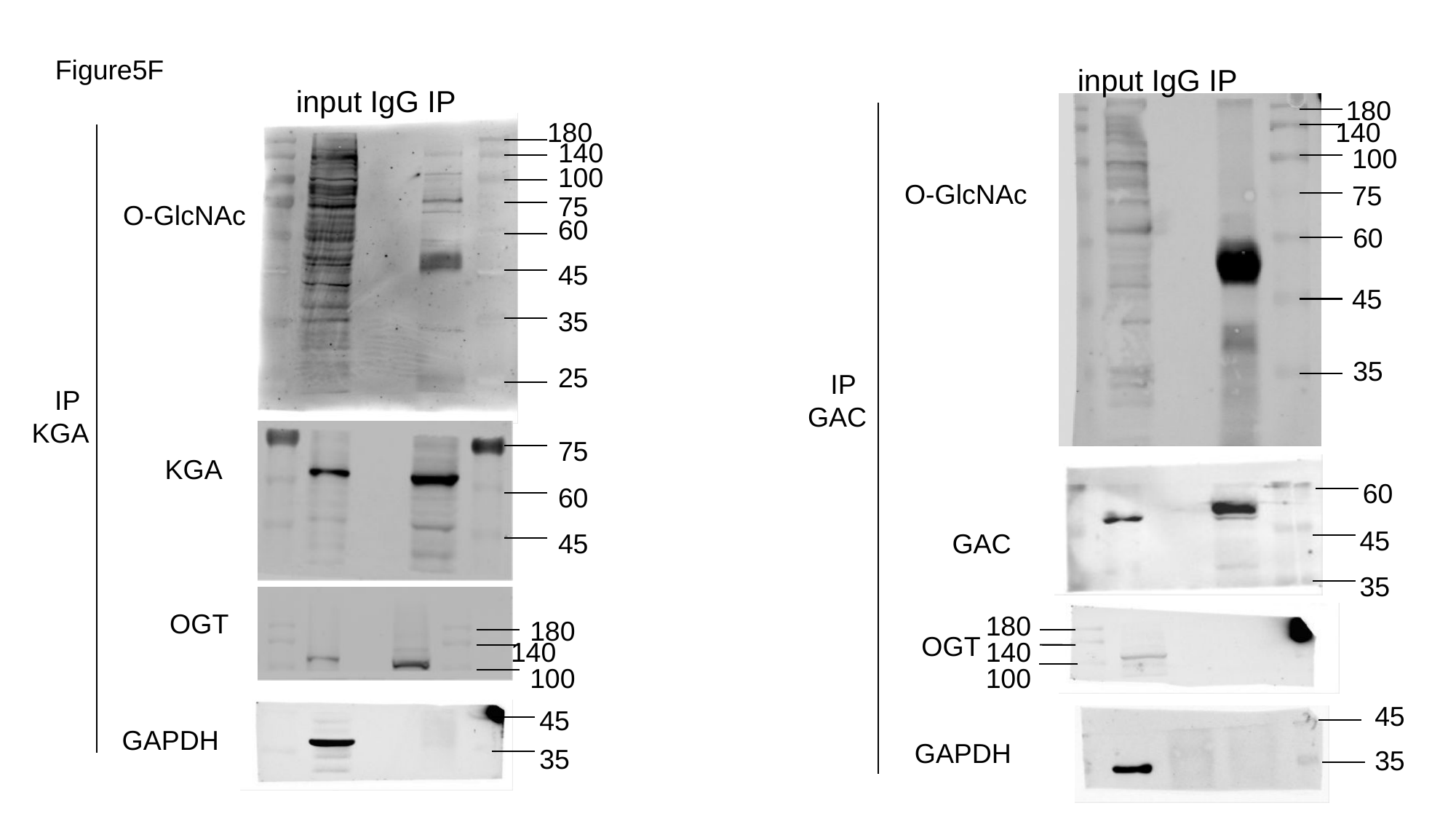

Figure5F
input IgG IP
input IgG IP
180
180
140
140
100
100
O-GlcNAc
75
75
O-GlcNAc
60
60
45
45
35
35
25
 IP
GAC
 IP
KGA
75
KGA
60
60
45
45
GAC
35
OGT
180
180
OGT
140
140
100
100
45
45
GAPDH
GAPDH
35
35

## Slide 11
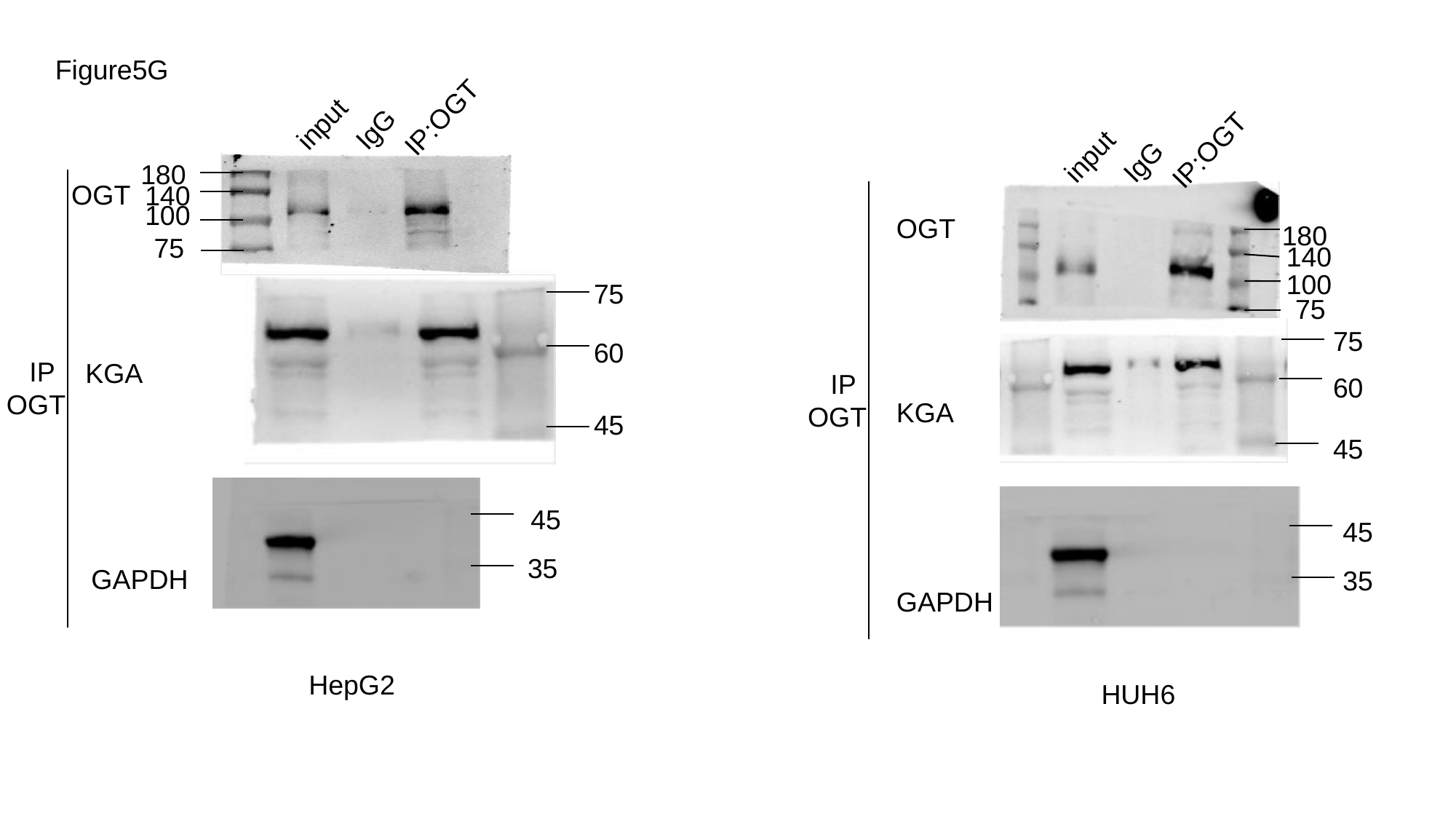

Figure5G
IP:OGT
input
IgG
IP:OGT
input
IgG
180
OGT
140
100
OGT
180
75
140
100
75
75
75
60
 IP
OGT
KGA
 IP
OGT
60
KGA
45
45
45
45
35
GAPDH
35
GAPDH
HepG2
HUH6

## Slide 12
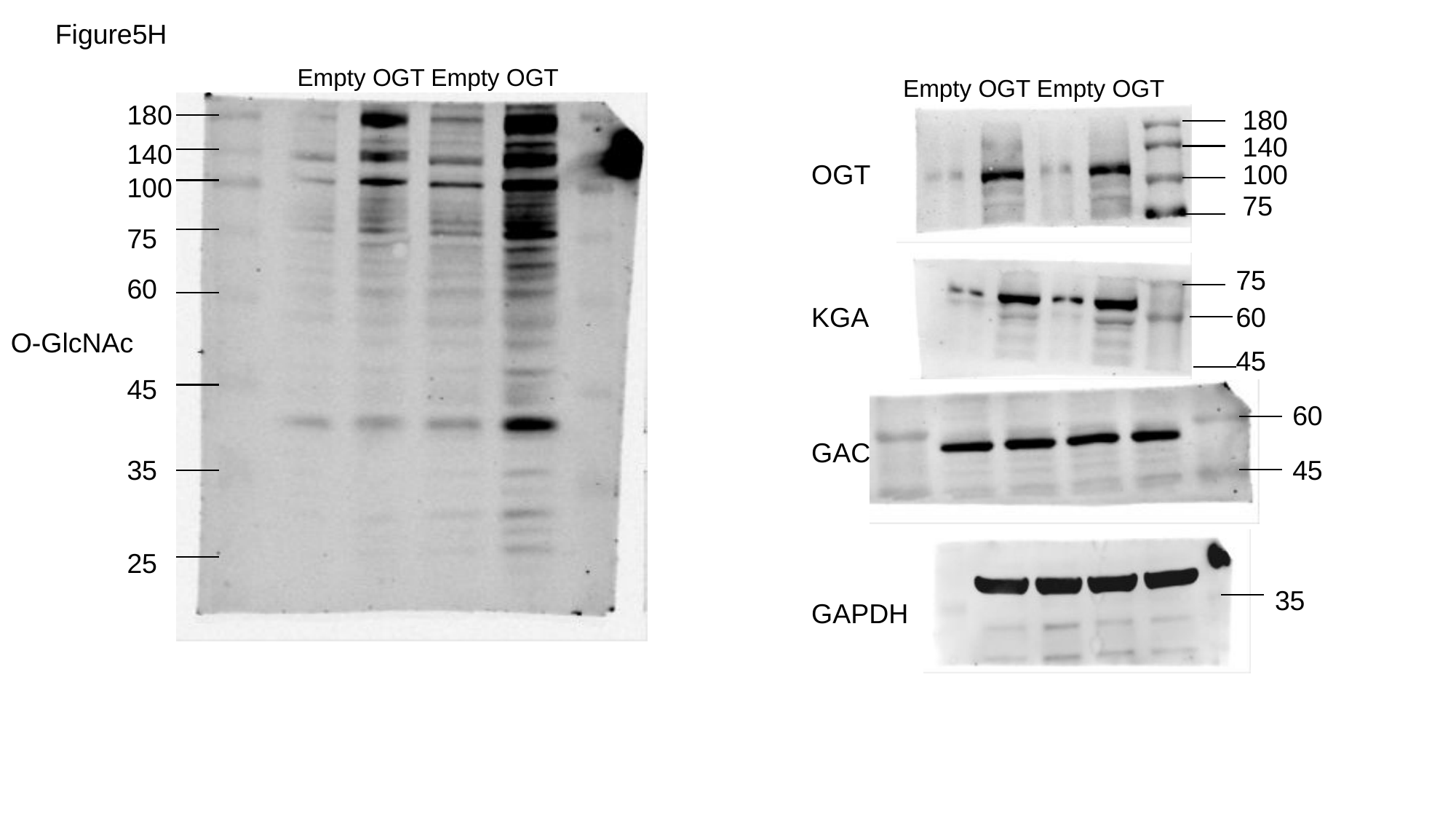

Figure5H
Empty OGT Empty OGT
Empty OGT Empty OGT
180
180
140
140
OGT
100
100
75
75
75
60
KGA
60
O-GlcNAc
45
45
60
GAC
35
45
25
35
GAPDH

## Slide 13
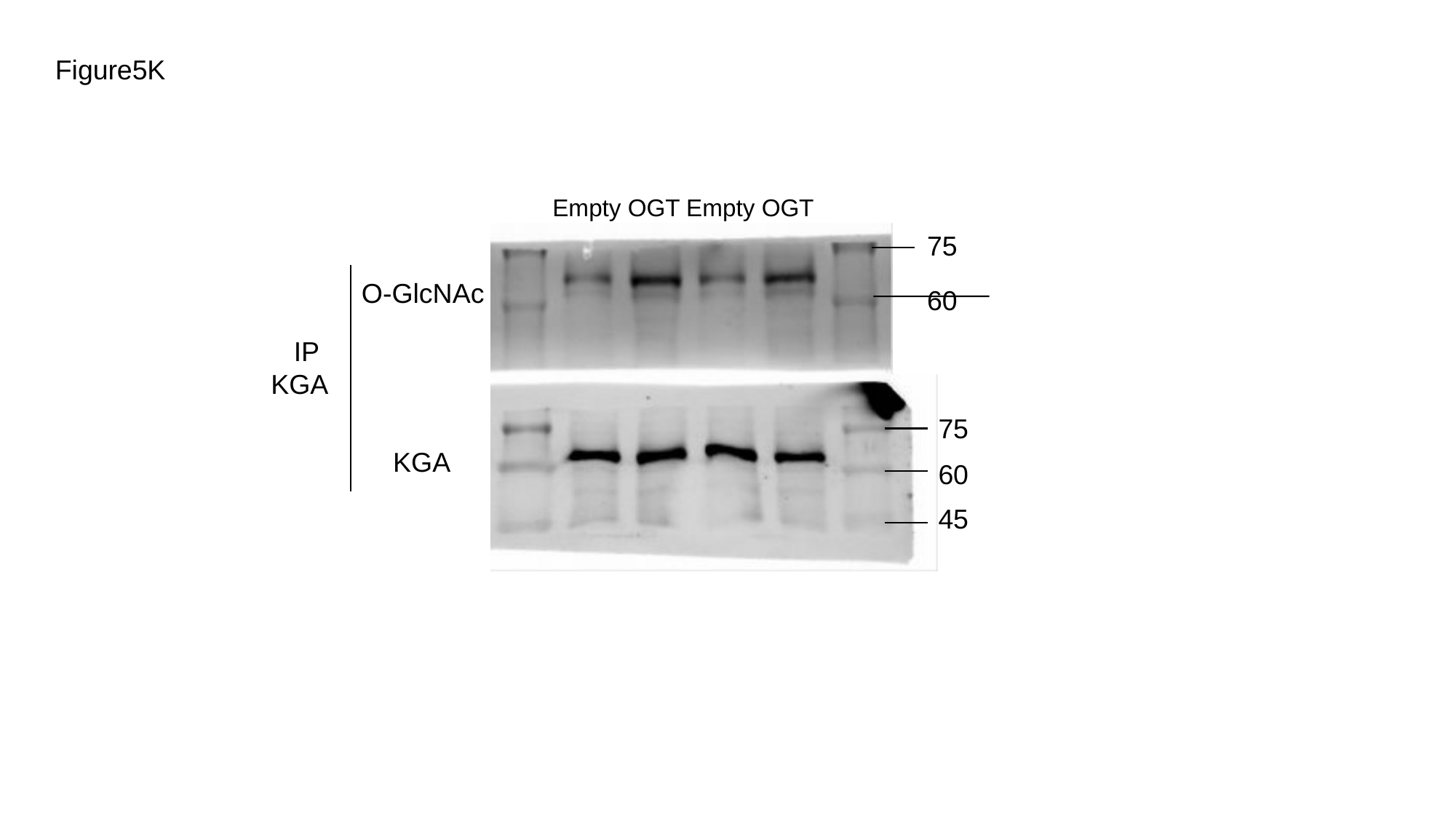

Figure5K
Empty OGT Empty OGT
75
O-GlcNAc
60
 IP
KGA
75
KGA
60
45

## Slide 14
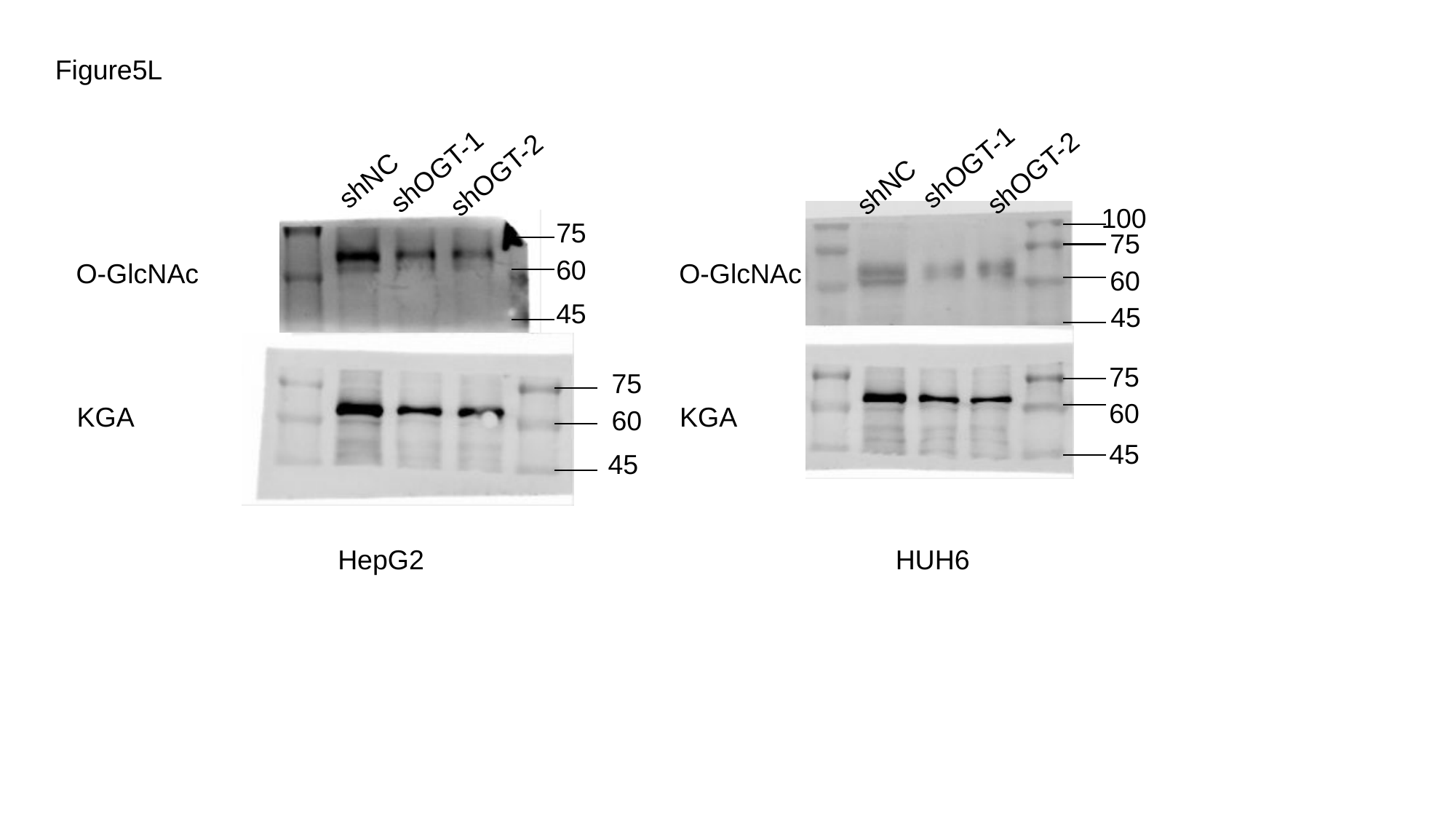

Figure5L
shOGT-1
shOGT-1
shOGT-2
shOGT-2
shNC
shNC
100
75
75
60
O-GlcNAc
O-GlcNAc
60
45
45
75
75
60
KGA
KGA
60
45
45
HepG2
HUH6

## Slide 15
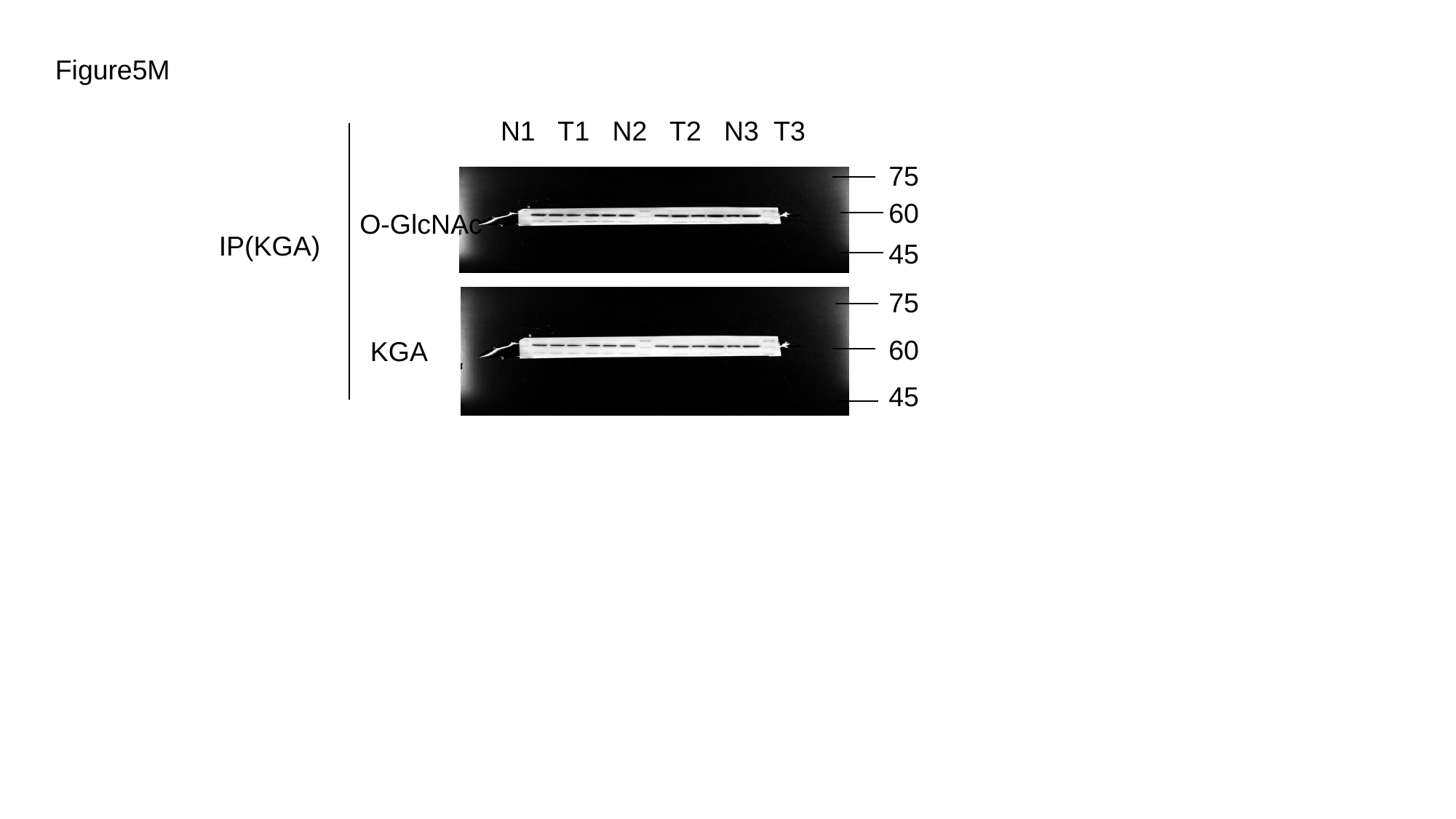

Figure5M
N1 T1 N2 T2 N3 T3
75
60
O-GlcNAc
IP(KGA)
45
75
60
KGA
45

## Slide 16
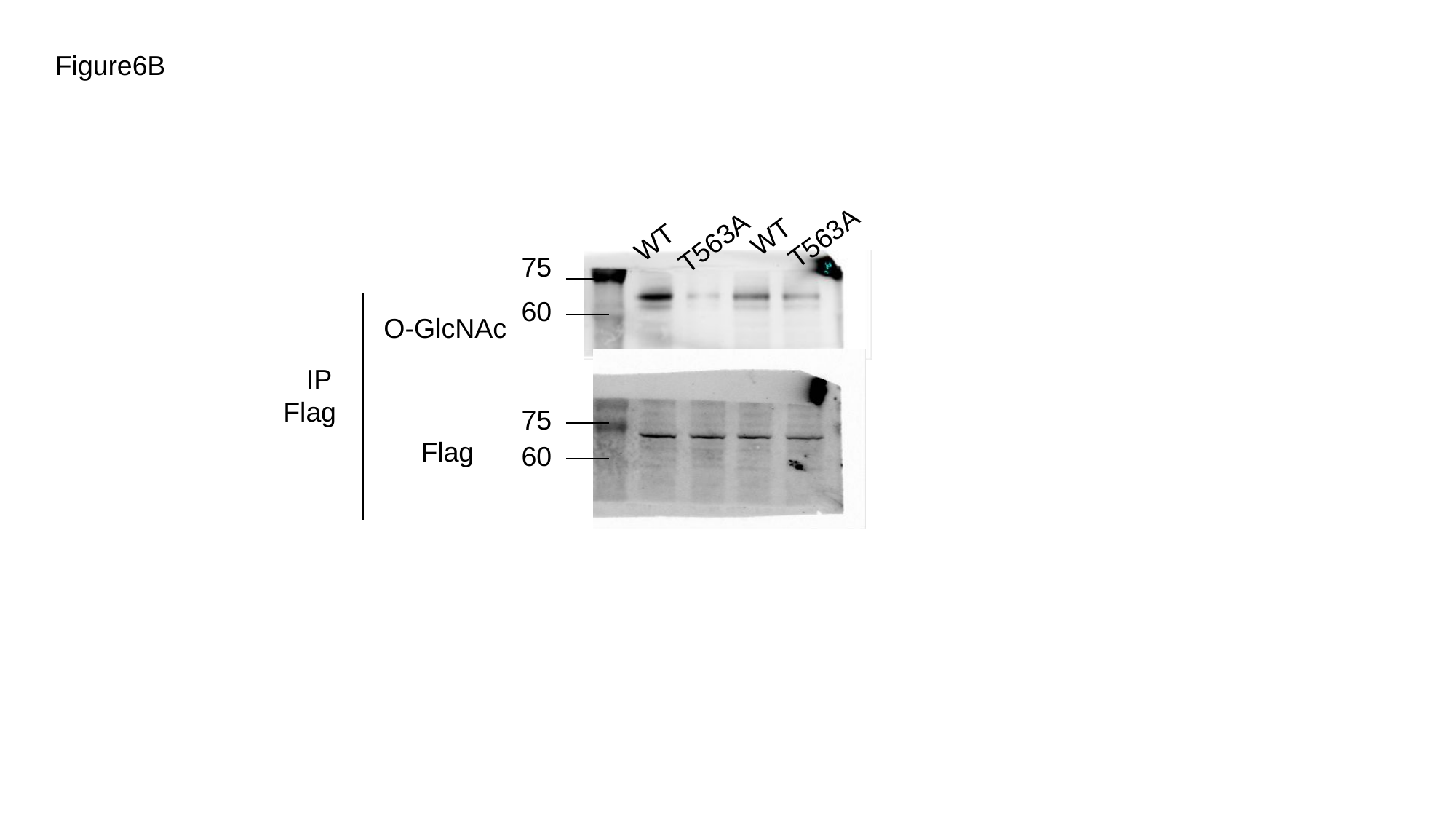

Figure6B
WT
T563A
WT
T563A
75
60
O-GlcNAc
 IP
Flag
75
Flag
60

## Slide 17
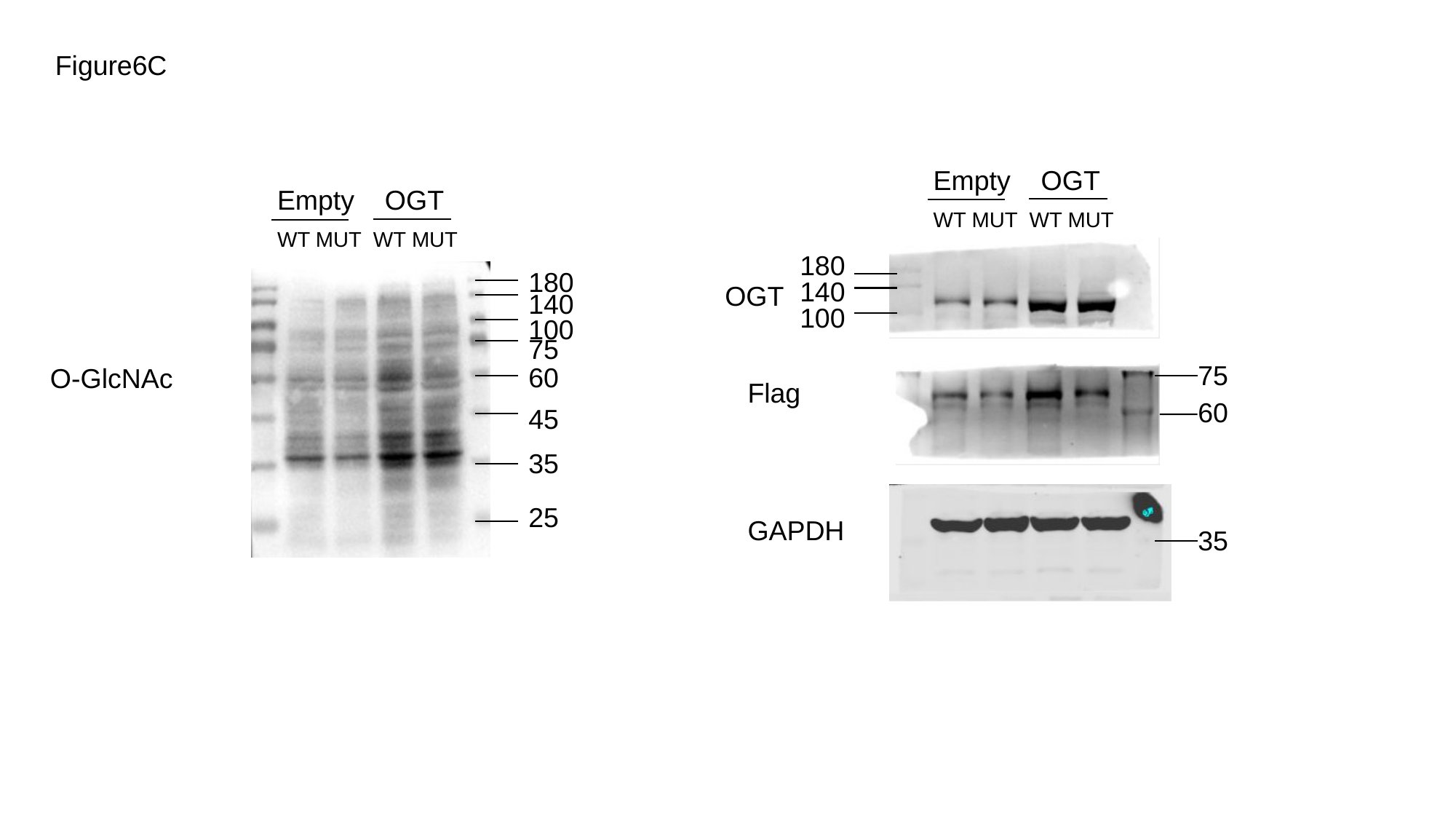

Figure6C
Empty OGT
Empty OGT
WT MUT WT MUT
WT MUT WT MUT
180
180
140
OGT
140
100
100
75
75
60
O-GlcNAc
Flag
60
45
35
25
GAPDH
35

## Slide 18
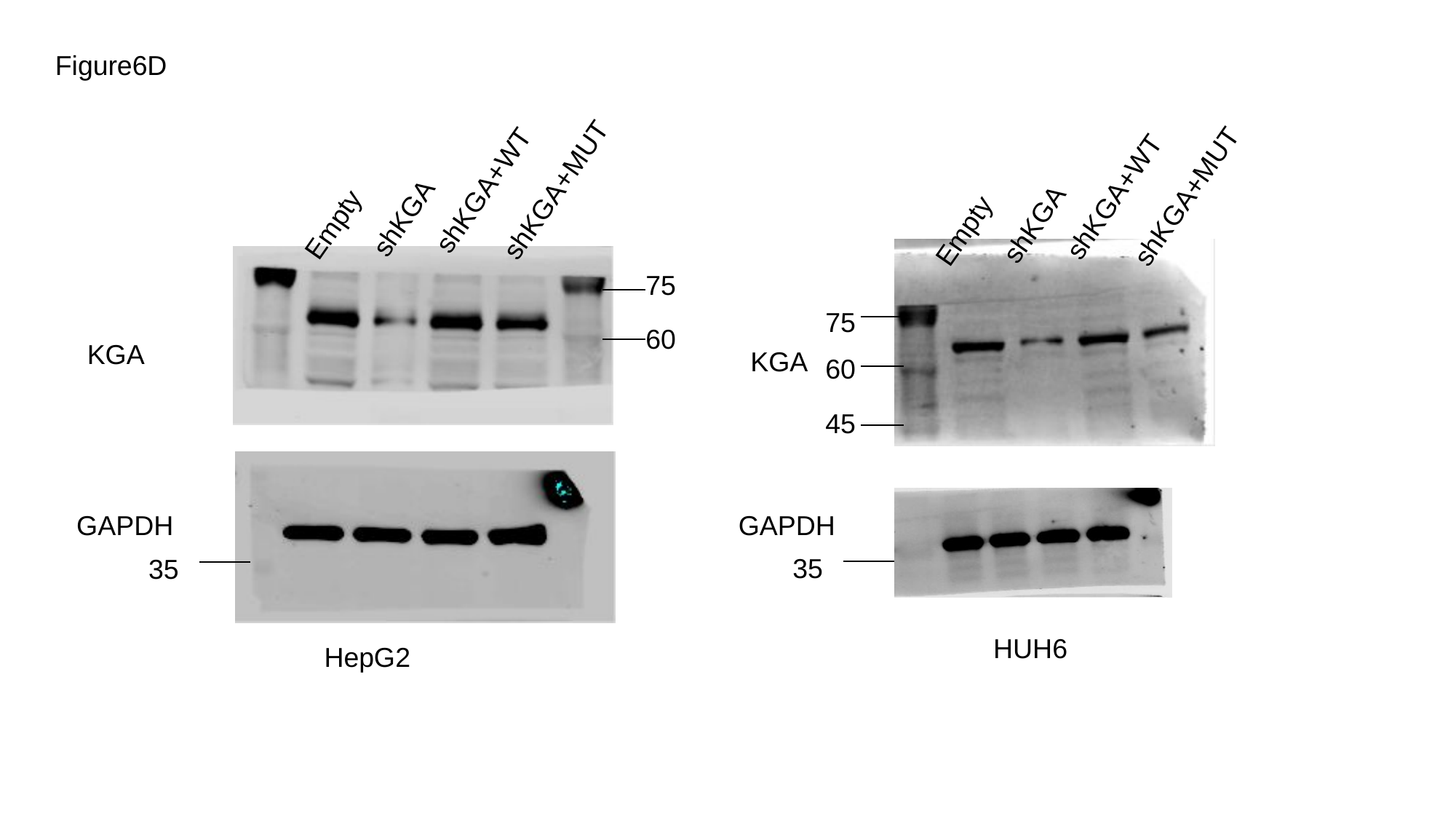

Figure6D
shKGA+MUT
shKGA+MUT
 shKGA+WT
 shKGA+WT
Empty
Empty
 shKGA
 shKGA
75
75
60
KGA
KGA
60
45
GAPDH
GAPDH
35
35
HUH6
HepG2

## Slide 19
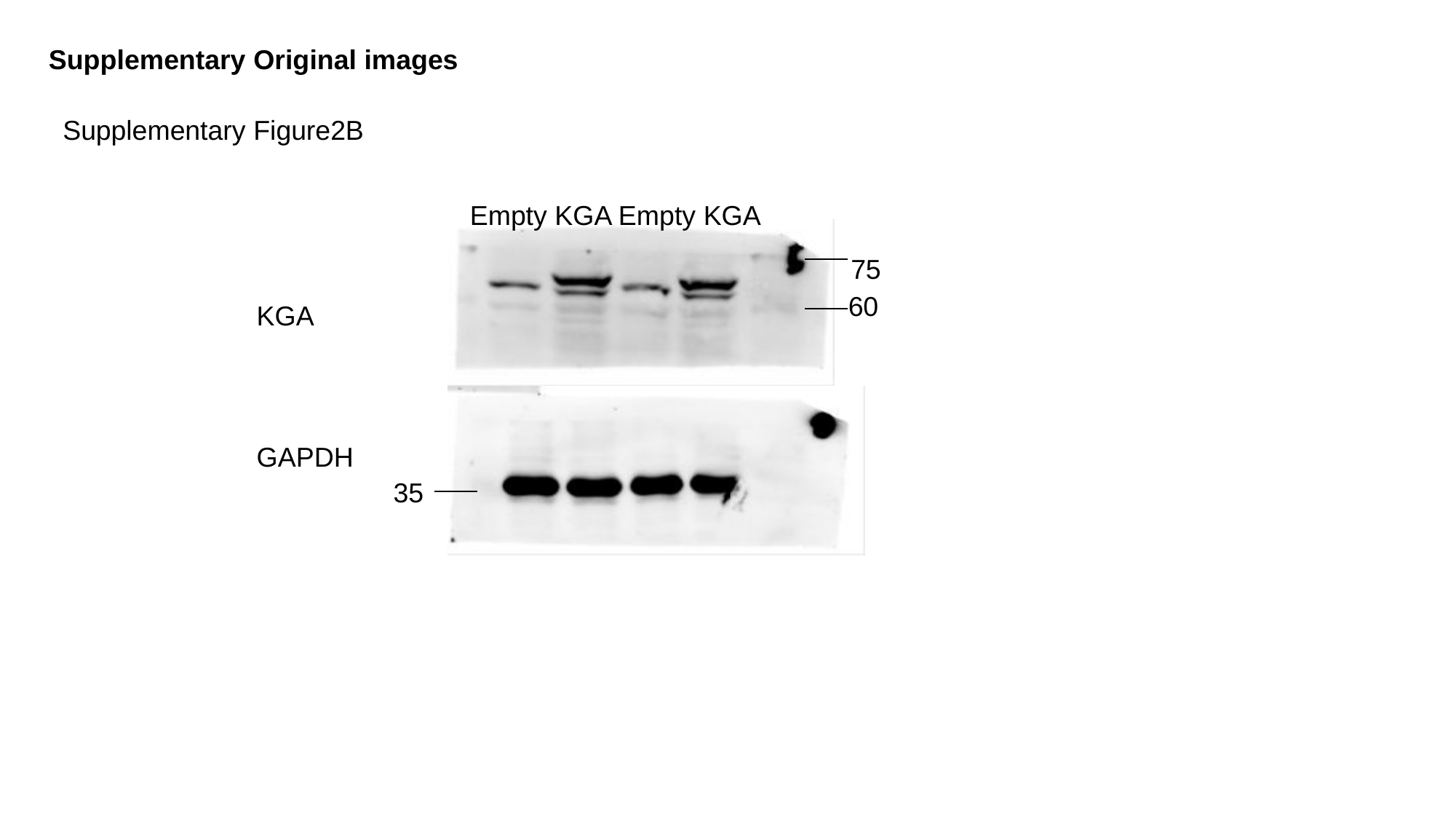

Supplementary Original images
Supplementary Figure2B
Empty KGA Empty KGA
75
60
KGA
GAPDH
35

## Slide 20
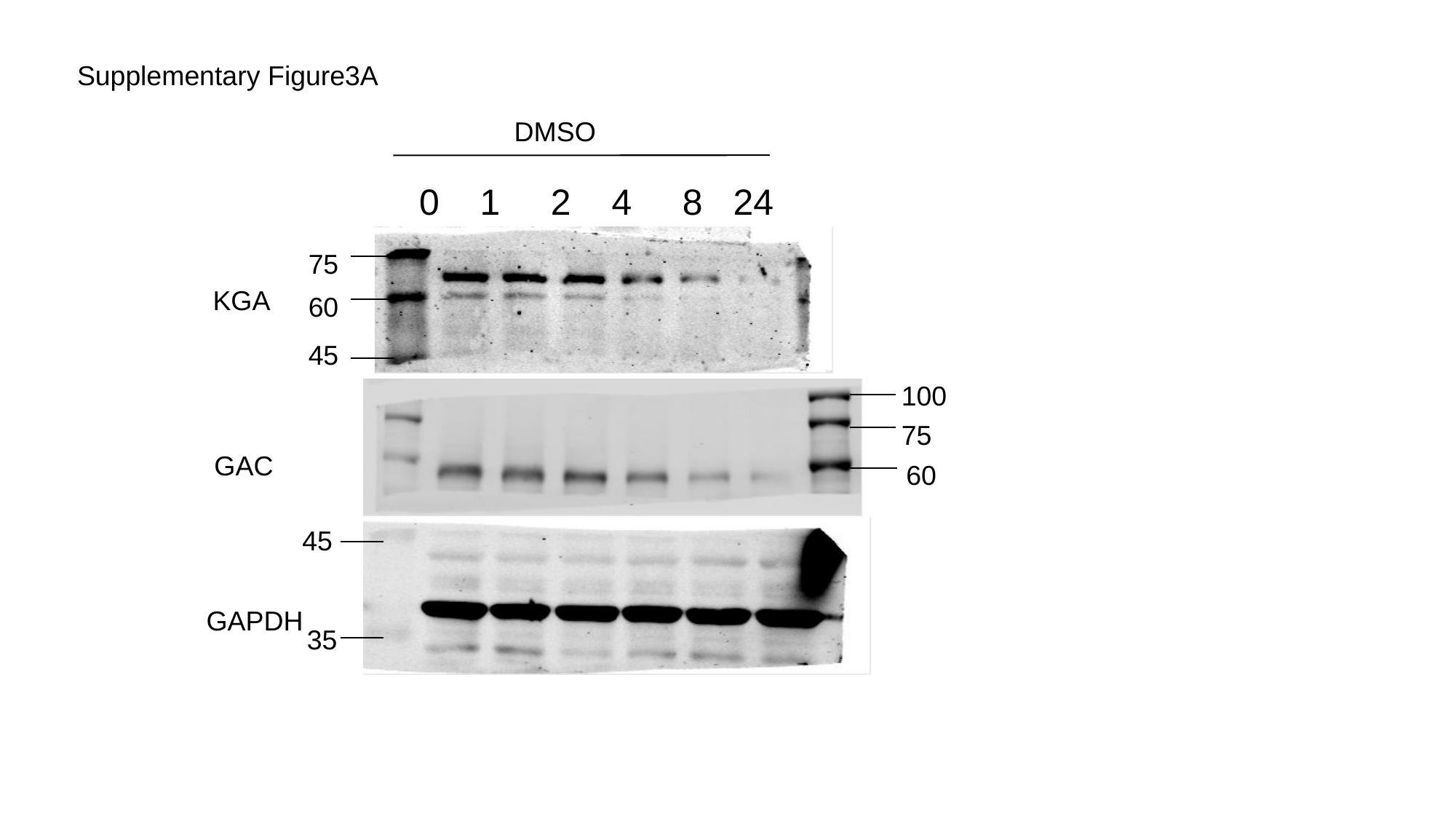

Supplementary Figure3A
DMSO
0 1 2 4 8 24
75
KGA
60
45
100
75
GAC
60
45
GAPDH
35

## Slide 21
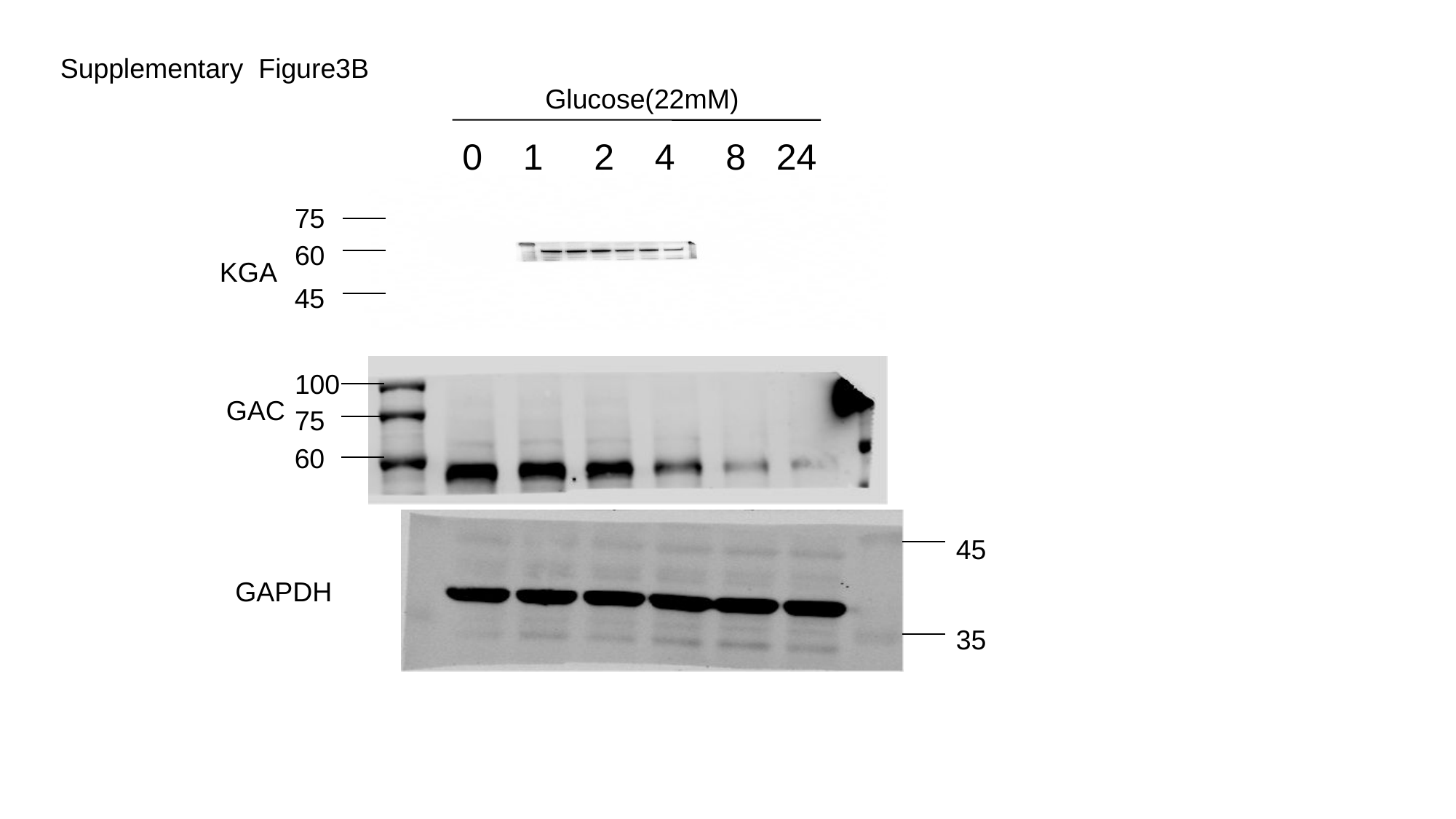

Supplementary Figure3B
Glucose(22mM)
0 1 2 4 8 24
75
60
KGA
45
100
GAC
75
60
45
GAPDH
35

## Slide 22
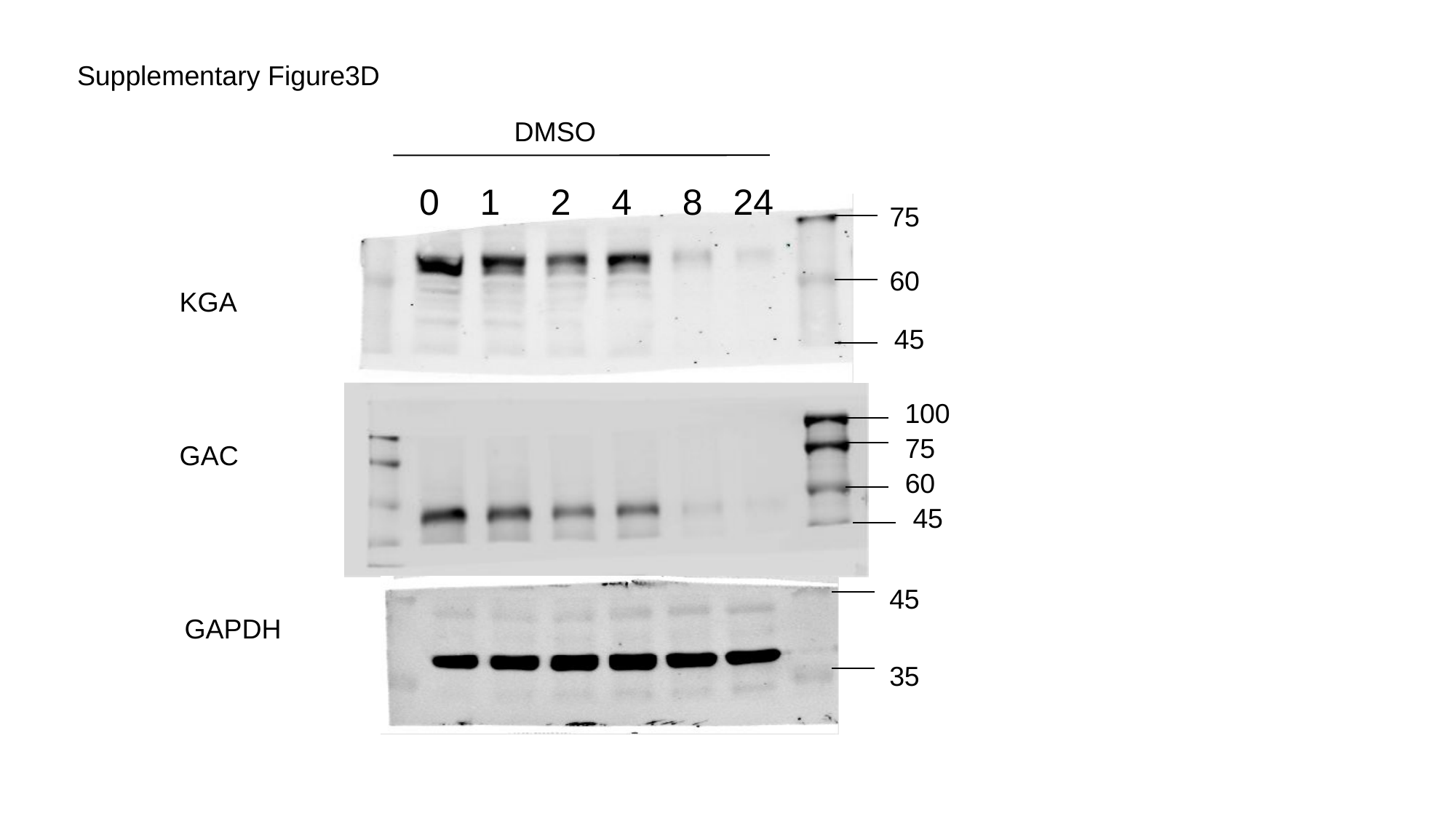

Supplementary Figure3D
DMSO
0 1 2 4 8 24
75
60
KGA
45
100
75
GAC
60
45
45
GAPDH
35

## Slide 23
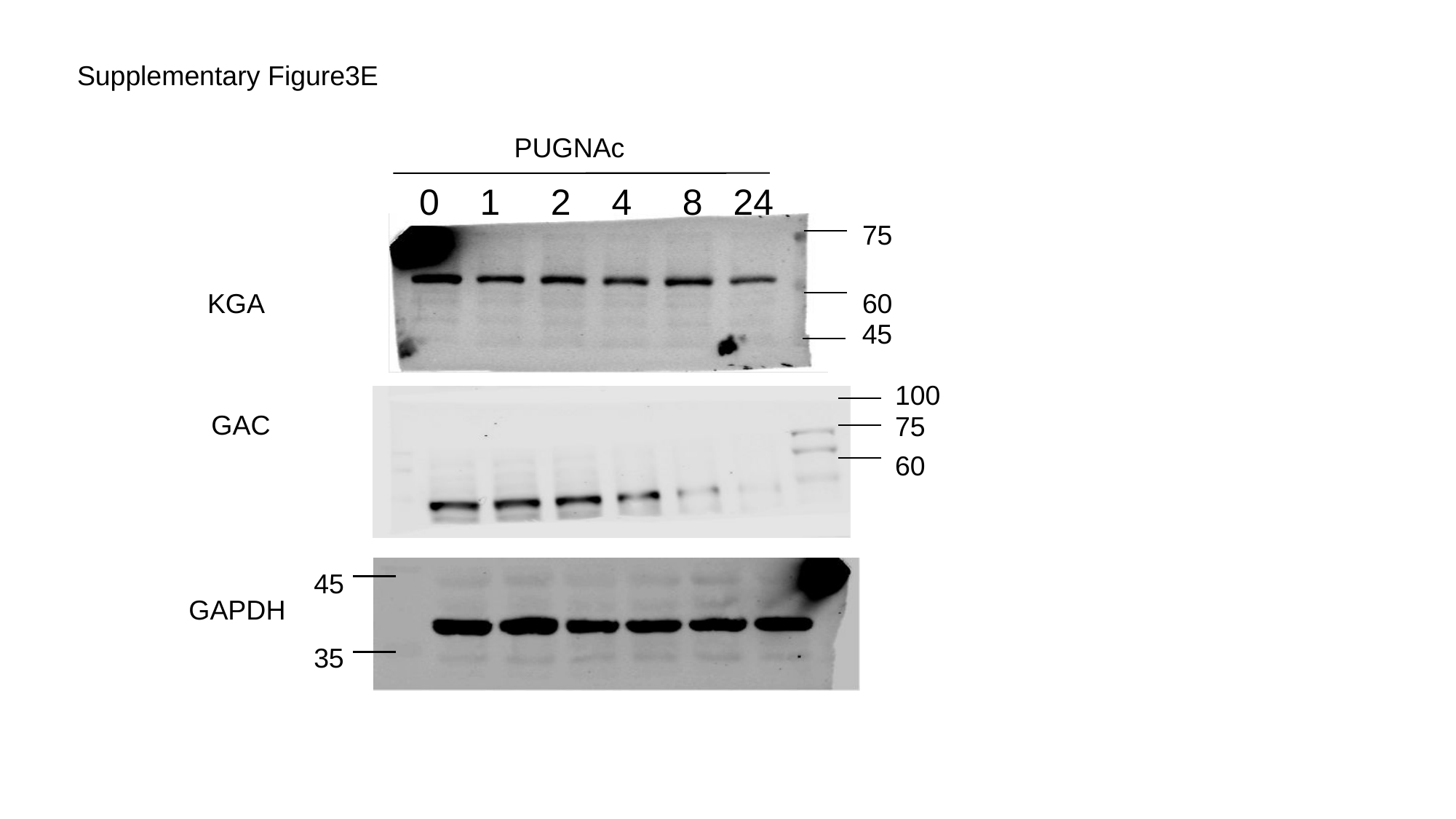

Supplementary Figure3E
PUGNAc
0 1 2 4 8 24
75
KGA
60
45
100
GAC
75
60
45
GAPDH
35

## Slide 24
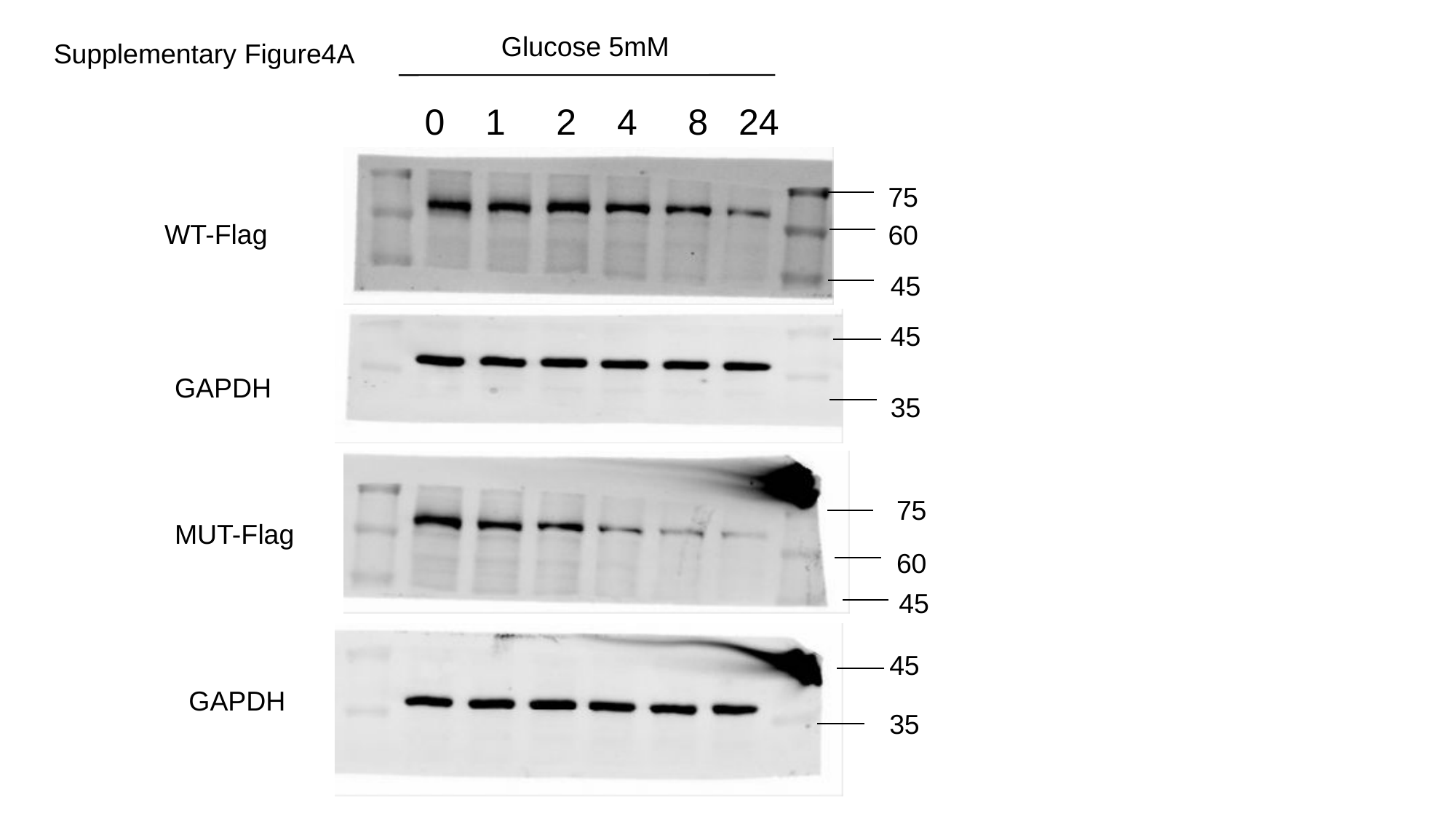

Glucose 5mM
Supplementary Figure4A
0 1 2 4 8 24
75
WT-Flag
60
45
45
GAPDH
35
75
MUT-Flag
60
45
45
GAPDH
35

## Slide 25
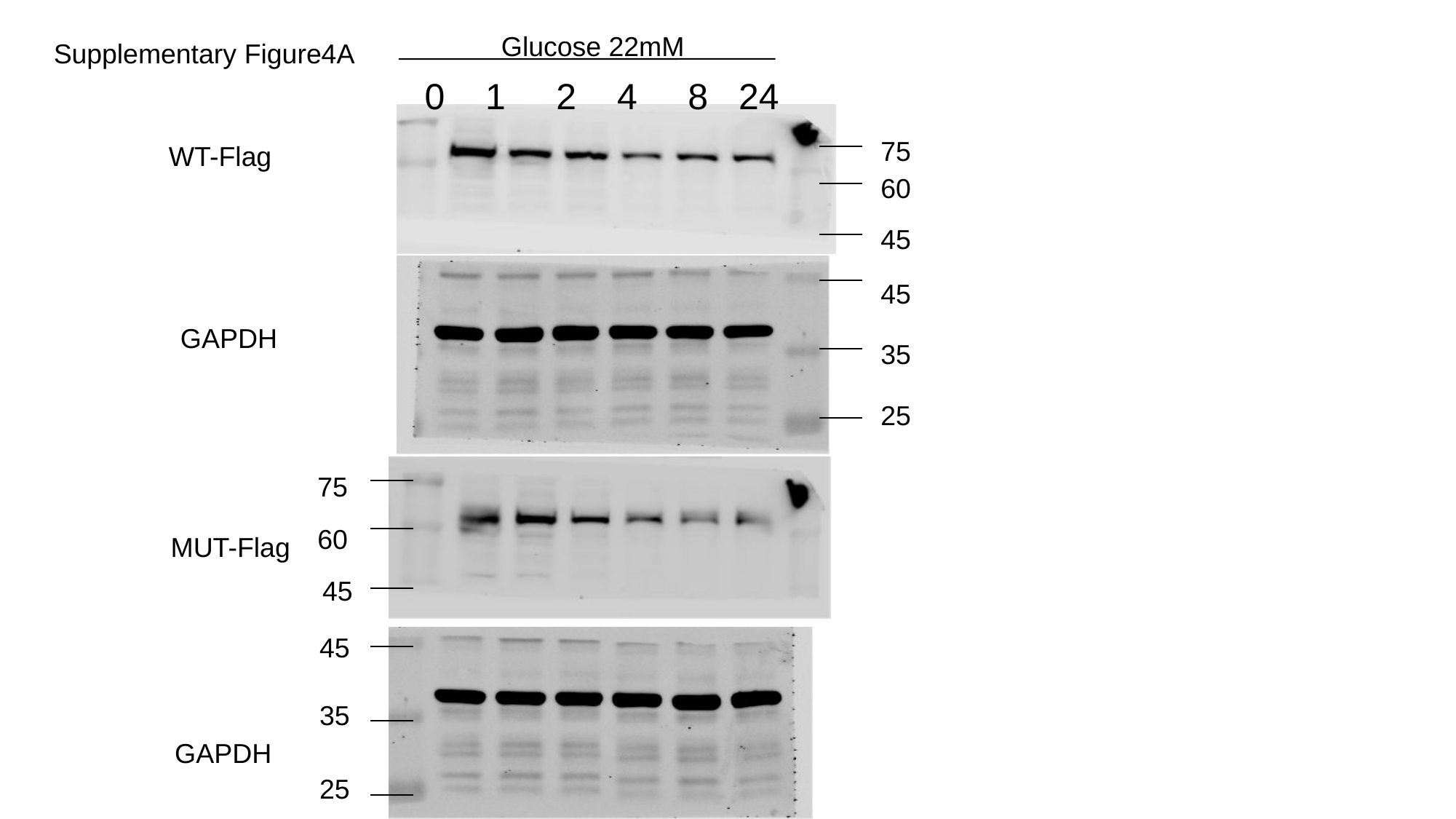

Glucose 22mM
Supplementary Figure4A
0 1 2 4 8 24
75
WT-Flag
60
45
45
GAPDH
35
25
75
60
MUT-Flag
45
45
35
GAPDH
25

## Slide 26
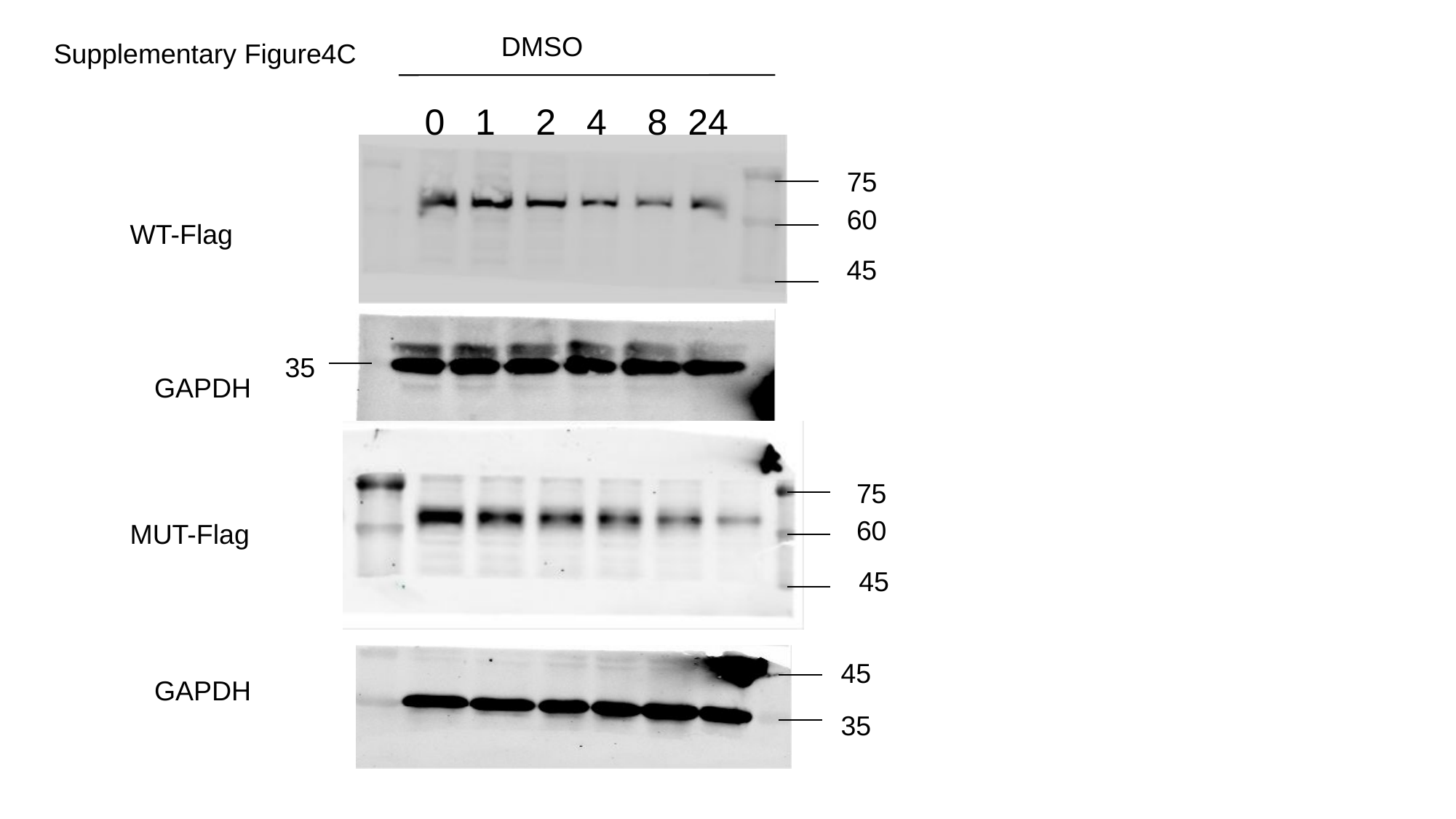

DMSO
Supplementary Figure4C
0 1 2 4 8 24
75
60
WT-Flag
45
35
GAPDH
75
60
MUT-Flag
45
45
GAPDH
35

## Slide 27
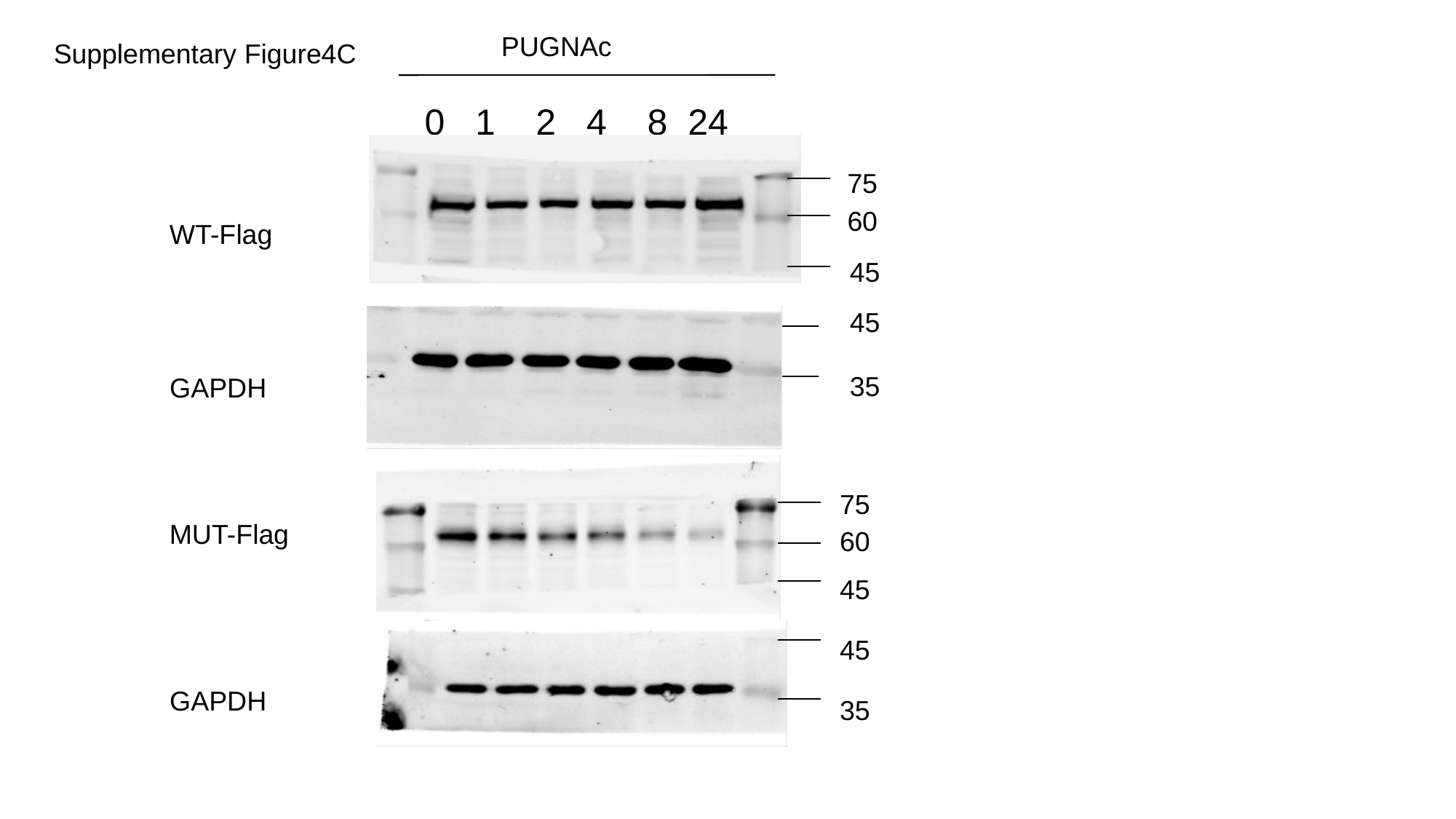

PUGNAc
Supplementary Figure4C
0 1 2 4 8 24
75
60
WT-Flag
45
45
35
GAPDH
75
MUT-Flag
60
45
45
GAPDH
35

## Slide 28
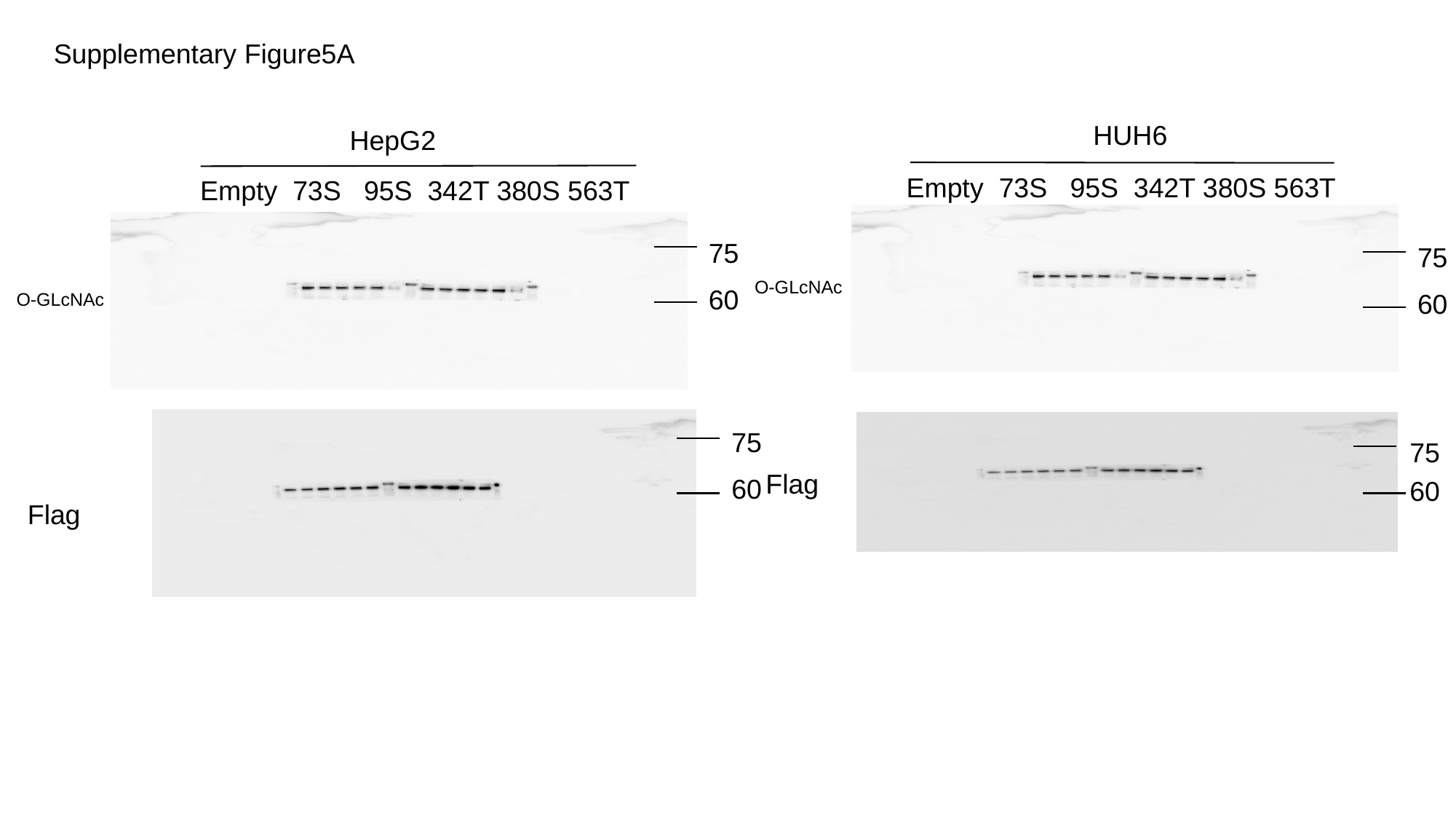

Supplementary Figure5A
HUH6
HepG2
Empty 73S 95S 342T 380S 563T
Empty 73S 95S 342T 380S 563T
75
75
O-GLcNAc
60
60
O-GLcNAc
75
75
Flag
60
60
Flag

## Slide 29
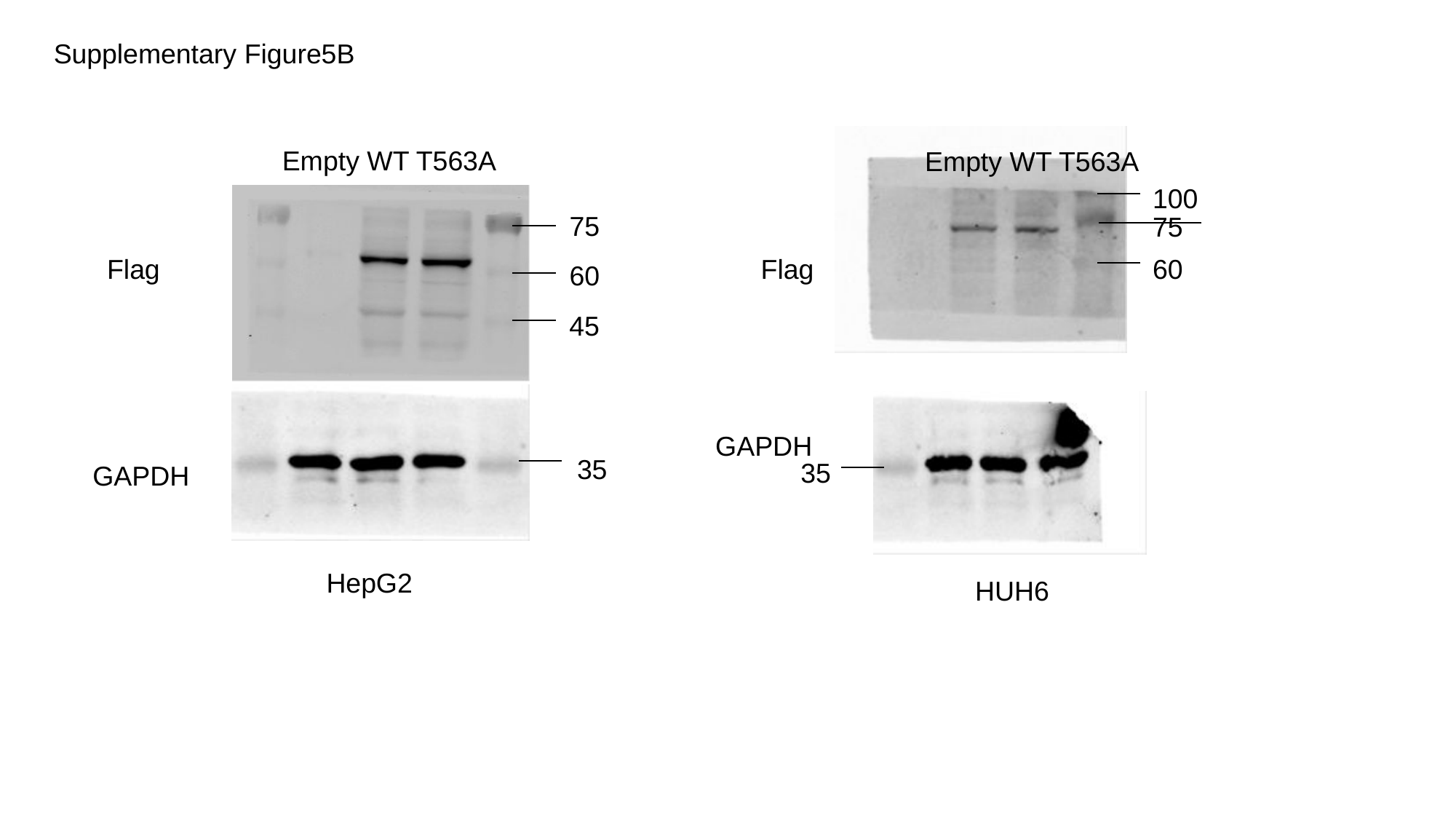

Supplementary Figure5B
Empty WT T563A
Empty WT T563A
100
75
75
Flag
Flag
60
60
45
GAPDH
35
35
GAPDH
HepG2
HUH6

## Slide 30
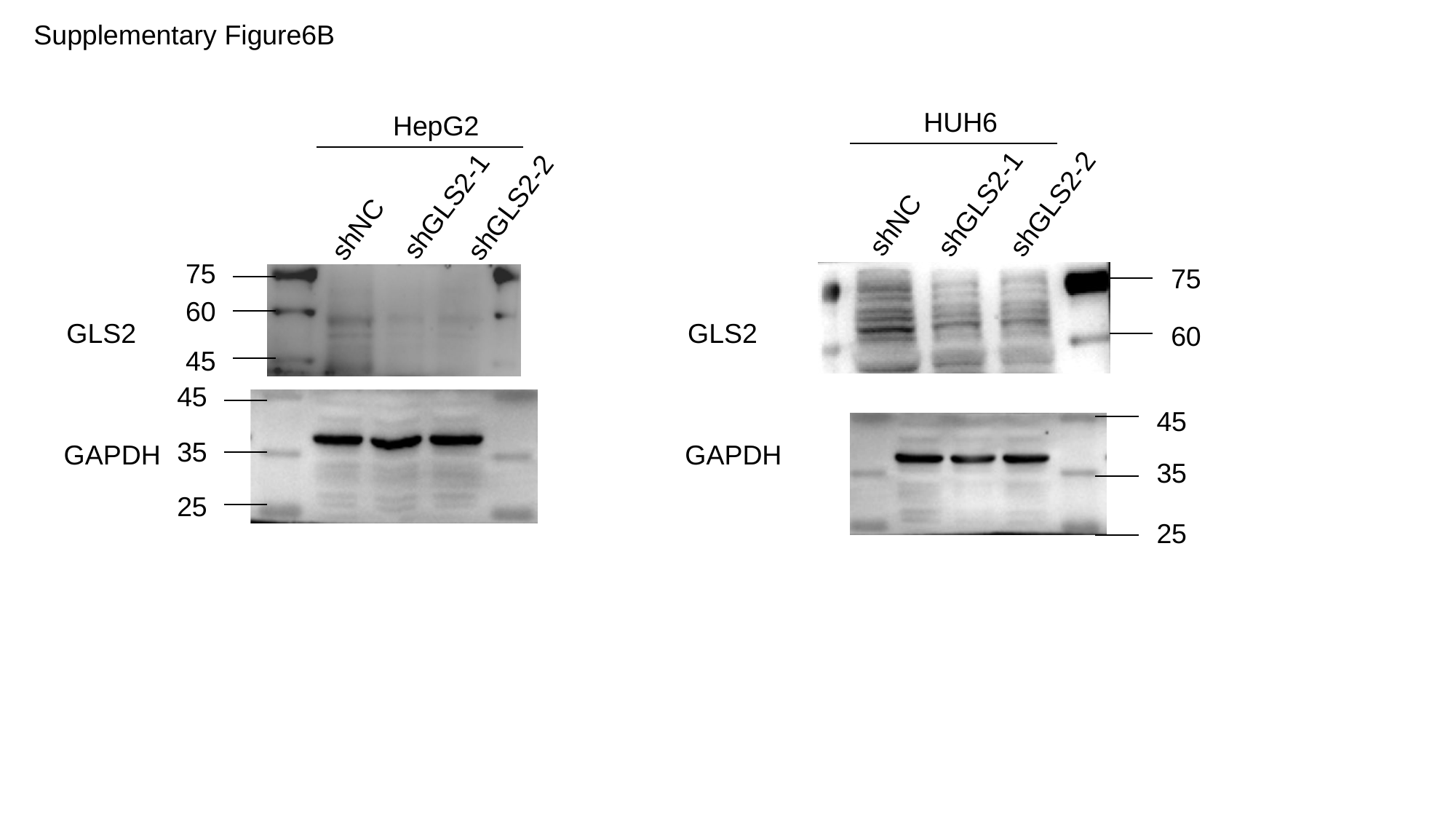

Supplementary Figure6B
HUH6
HepG2
shGLS2-2
shGLS2-1
shGLS2-1
shGLS2-2
shNC
shNC
75
75
60
GLS2
GLS2
60
45
45
45
35
GAPDH
GAPDH
35
25
25

## Slide 31
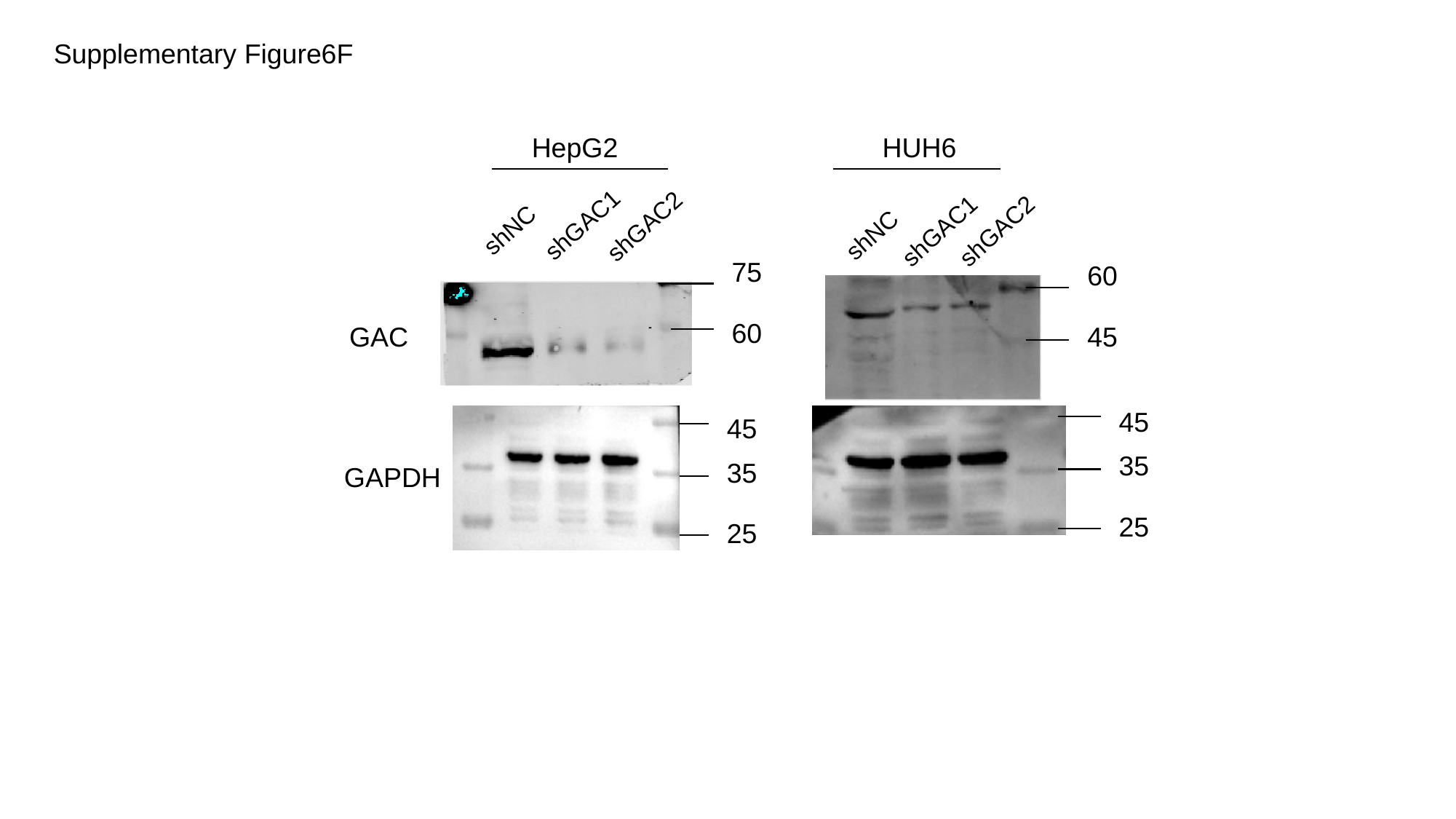

Supplementary Figure6F
HepG2
 HUH6
shGAC1
shGAC2
shGAC1
shGAC2
shNC
shNC
75
60
60
GAC
45
45
45
35
35
GAPDH
25
25

## Slide 32
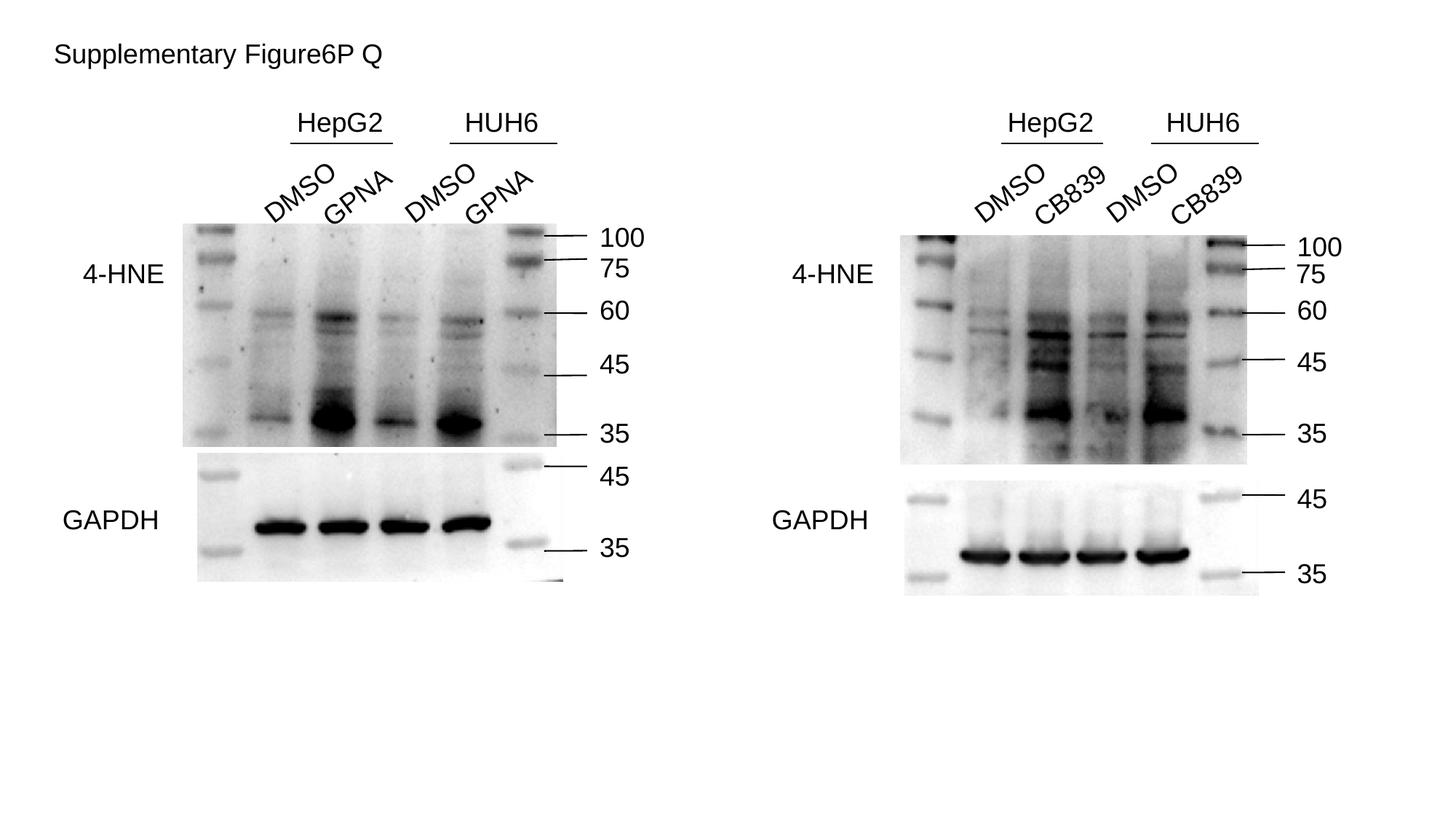

Supplementary Figure6P Q
HepG2
HUH6
HepG2
HUH6
DMSO
DMSO
DMSO
DMSO
GPNA
GPNA
CB839
CB839
100
100
75
4-HNE
4-HNE
75
60
60
45
45
35
35
45
45
GAPDH
GAPDH
35
35

## Slide 33
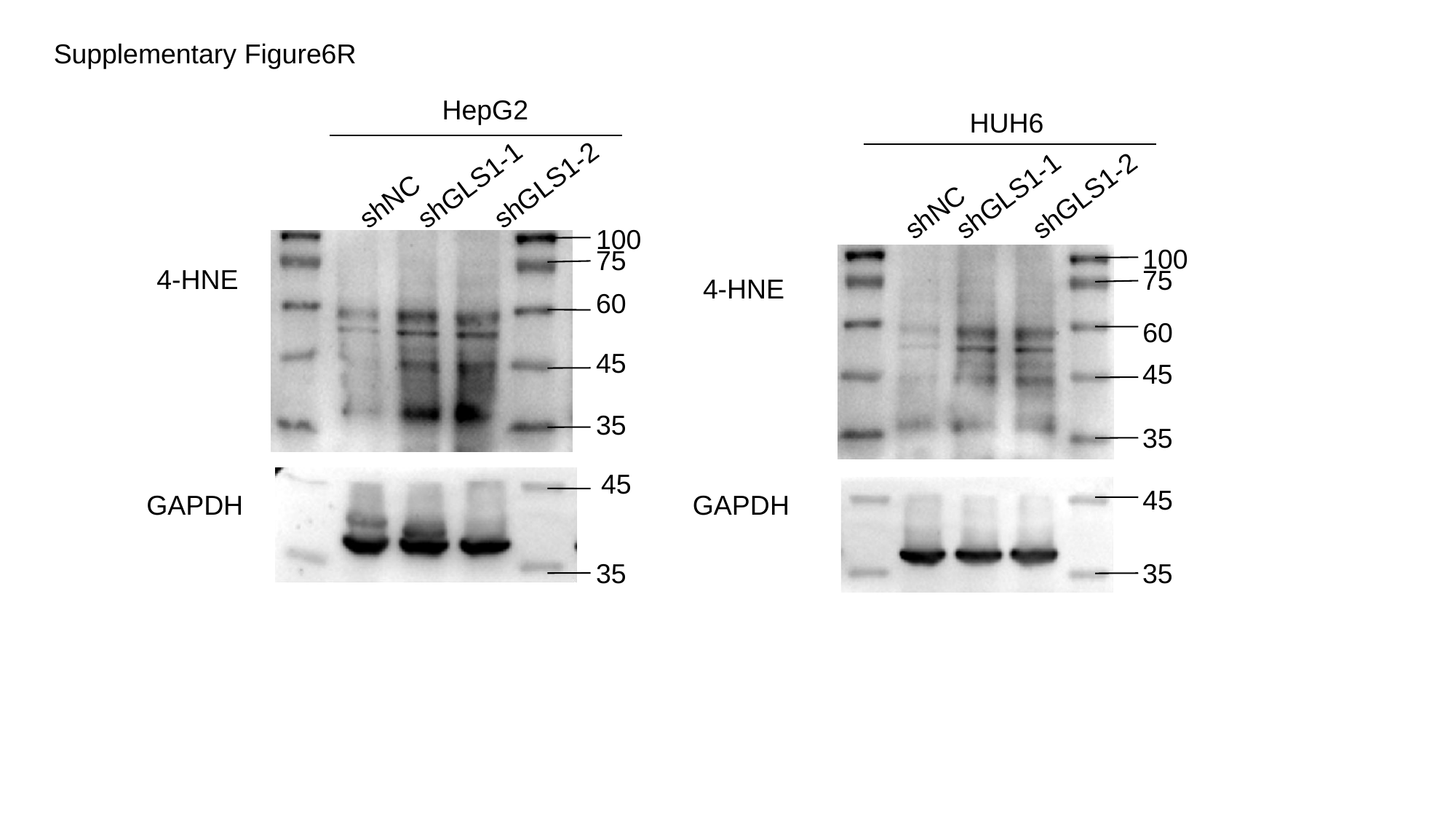

Supplementary Figure6R
HepG2
HUH6
shGLS1-2
shGLS1-1
shGLS1-2
shGLS1-1
shNC
shNC
100
100
75
4-HNE
75
4-HNE
60
60
45
45
35
35
45
45
GAPDH
GAPDH
35
35

## Slide 34
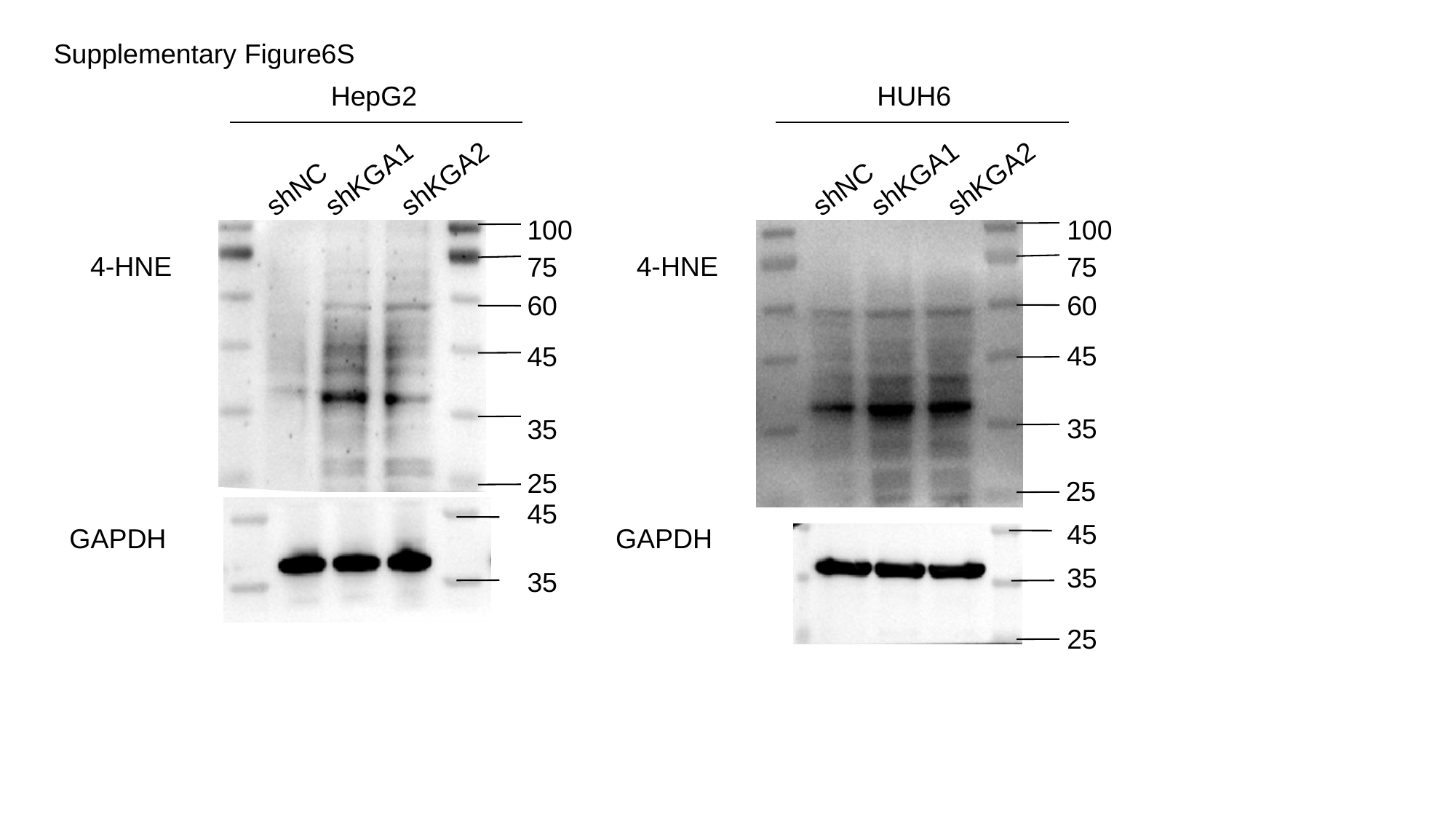

Supplementary Figure6S
HepG2
HUH6
shKGA1
shKGA1
shKGA2
shKGA2
shNC
shNC
100
100
4-HNE
4-HNE
75
75
60
60
45
45
35
35
25
25
45
45
GAPDH
GAPDH
35
35
25

## Slide 35
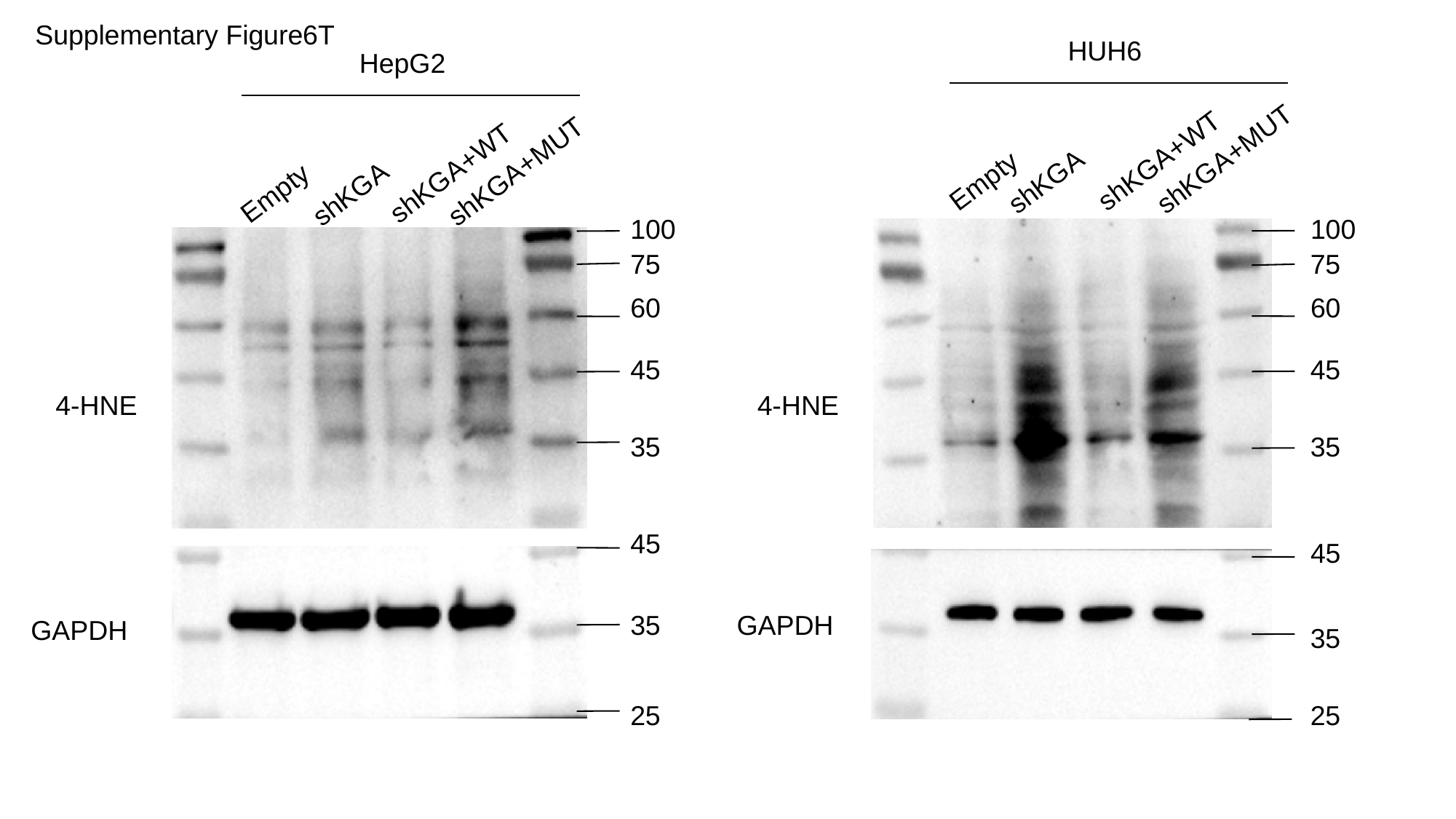

Supplementary Figure6T
HUH6
HepG2
shKGA+MUT
shKGA+WT
shKGA+MUT
shKGA+WT
Empty
shKGA
Empty
shKGA
100
100
75
75
60
60
45
45
4-HNE
4-HNE
35
35
45
45
35
GAPDH
GAPDH
35
25
25
